# Microtubule-Associated Protein EB3 Regulates IP_3_ Receptor Clustering and Ca^2+^ Signaling in Endothelial Cells

**DOI:** 10.1016/j.celrep.2015.06.001

**Published:** 2015-06-25

**Authors:** Melissa Geyer, Fei Huang, Ying Sun, Stephen M. Vogel, Asrar B. Malik, Colin W. Taylor, Yulia A. Komarova

**Affiliations:** 1 Department of Pharmacology and The Center for Lung and Vascular Biology, University of Illinois College of Medicine, Chicago, IL 60612, USA; 2 Department of Pharmacology, University of Cambridge, Cambridge CB2 1PD, UK

## Abstract

The mechanisms by which the microtubule cytoskeleton regulates the permeability of endothelial barrier are not well understood. Here, we demonstrate that microtubule-associated end-binding protein 3 (EB3), a core component of the microtubule plus-end protein complex, binds to inositol 1,4,5-trisphosphate receptors (IP_3_Rs) through an S/TxIP EB-binding motif. In endothelial cells, α-thrombin, a pro-inflammatory mediator that stimulates phospholipase Cβ, increases the cytosolic Ca^2+^ concentration and elicits clustering of IP_3_R3s. These responses, and the resulting Ca^2+^-dependent phosphorylation of myosin light chain, are prevented by depletion of either EB3 or mutation of the TxIP motif of IP_3_R3 responsible for mediating its binding to EB3. We also show that selective EB3 gene deletion in endothelial cells of mice abrogates α-thrombin-induced increase in endothelial permeability. We conclude that the EB3-mediated interaction of IP_3_Rs with microtubules controls the assembly of IP_3_Rs into effective Ca^2+^ signaling clusters, which thereby regulate microtubule-dependent endothelial permeability.

## Highlights

• IP_3_ receptors (IP_3_Rs) bind to microtubule end-binding protein EB3
• EB3, in turn, promotes IP_3_R clustering and Ca^2+^ signals in endothelial cells
• IP_3_R-EB3 interaction thereby contributes to endothelial barrier disruption
• In vivo EB3 deletion in endothelial cells protects from vascular hyperpermeability

## Graphical Abstract

**Figure:**
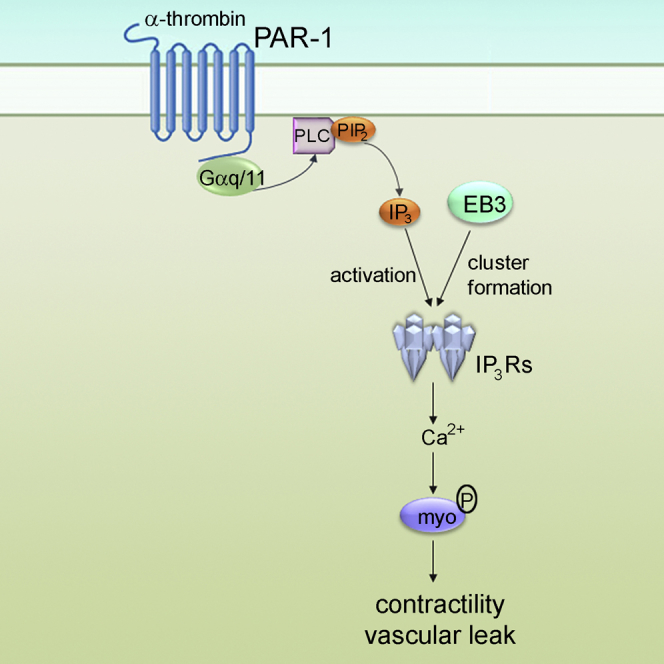

## Teaser

End binding proteins (EBs) mediate interactions between growing microtubules and intracellular structures. Geyer et al. demonstrate that interactions between EB3 and IP_3_ receptors control clustering of IP_3_ receptors in endothelial cells and Ca^2+^ signaling, and thus permeability of endothelial barrier in inflammatory diseases.

## Introduction

Adherens junctions (AJs) responsible for endothelial cell interactions (Dejana, 2004) and acto-myosin contraction (Wainwright et al., 2003) regulate the integrity of the vascular endothelial barrier. The ectodomain of vascular endothelial (VE)-cadherin, which is the primary adhesion molecule of AJs, undergoes homophilic *trans*-dimerization to form AJs, while its intracellular domain interacts with the actin cytoskeleton (Daneshjou et al., 2015, Giannotta et al., 2013). Activation of phospholipase C (PLC) via G protein-coupled receptors (GPCRs), such as the protease-activated receptor-1 (PAR-1), or by disruption of VE-cadherin *trans*-interactions (Komarova et al., 2012), causes increased endothelial permeability (Komarova and Malik, 2010). An increase in cytosolic Ca^2+^ concentration ([Ca^2+^]_c_), by promoting disassembly of AJs and acto-myosin-mediated contraction of endothelial cells, is a crucial signal mediating this increased endothelial permeability (Komarova and Malik, 2010).

The roles of actin polymerization and disassembly of AJs in increasing endothelial permeability have received considerable attention, but microtubules may also contribute to the response by poorly understood mechanisms (Vogel and Malik, 2012). Microtubules are polarized tubular filaments of heterodimers of α- and β-tubulin, with distinct plus and minus ends (Howard and Hyman, 2003). Plus ends, usually directed toward the cell periphery, undergo cycles of polymerization and depolymerization regulated by plus-end tracking proteins (+TIPs) (Akhmanova and Steinmetz, 2010). The end-binding proteins, EB1 and EB3, members of the RP/EB family, are core elements of the dynamic +TIP complex. EBs transiently bind to growing microtubules by recognizing the GTP-bound state of β-tubulin (Maurer et al., 2012). EB binding enhances lateral contacts between tubulin molecules and prevents the transition from microtubule growth to shrinkage (“catastrophe” events) (Komarova et al., 2009, Maurer et al., 2012). In addition, EBs provide an essential hub for assembly of other +TIPs that facilitate interactions of microtubules with various macromolecules and organelles (Akhmanova and Steinmetz, 2010). The latter include endoplasmic reticulum (ER), which is continuously remodeled through its interactions with microtubules (Pendin et al., 2011). These interactions involve both tethering of the ER protein, stromal interaction molecule 1 (STIM1), to the +TIP complex by EB1, and the association of ER tubules with the plus-end directed microtubule motor protein, kinesin 1 (Friedman and Voeltz, 2011, Grigoriev et al., 2008).

ER is the major intracellular Ca^2+^ store. Inositol 1,4,5-trisphosphate receptors (IP_3_Rs) within ER membranes allow rapid release of Ca^2+^ from the ER (Foskett et al., 2007, Taylor et al., 2014). Emptying of ER Ca^2+^ stores then causes clustering of STIM1, and this activates store-operated Ca^2+^ entry into the cell across the plasma membrane (Wu et al., 2014). The release of Ca^2+^ from the ER evoked by IP_3_ proceeds through recruitment of Ca^2+^ release events that depend on IP_3_ priming IP_3_Rs to respond to Ca^2+^. This form of regulation allows clustered IP_3_Rs to stimulate the activity of their neighbors by Ca^2+^-induced Ca^2+^ release (Smith et al., 2009). The lowest concentrations of IP_3_ stimulate openings of single IP_3_Rs, and as the IP_3_ concentration increases the Ca^2+^ released by active IP_3_Rs is thought to stimulate coordinated opening of IP_3_Rs within a cluster, generating a Ca^2+^ “puff.” Further increases in IP_3_ concentration ignite regenerative Ca^2+^ waves that spread across the cell (Smith et al., 2009). The recruitment of IP_3_R activity depends critically on the distribution of IP_3_Rs in ER membranes, wherein most IP_3_Rs appear to be mobile (Ferreri-Jacobia et al., 2005, Pantazaka and Taylor, 2011). Stimulation of PLC causes reversible clustering of IP_3_Rs in cells (Chalmers et al., 2006, Tateishi et al., 2005) and within nuclear envelope IP_3_ causes IP_3_Rs to cluster (Taufiq-Ur-Rahman et al., 2009). Importantly in the context of the present study, IP_3_Rs associate with microtubules (Takei et al., 1998) and the association contributes to the redistribution of IP_3_Rs during sustained stimulation (Vermassen et al., 2003), extension of neuronal growth cones (Zhang and Forscher, 2009), and cell division (Mitsuyama and Sawai, 2001). There is, however, also evidence that IP_3_R clustering can persist after disruption of microtubules (Wilson et al., 1998, Taufiq-Ur-Rahman et al., 2009).

Studies using drugs (colchicine, nocodazole, and taxol) suppressing microtubule dynamics suggest an important role of microtubule cytoskeleton in organizing IP_3_-evoked Ca^2+^ signals (Isshiki et al., 1998, Fogarty et al., 2000). Perturbing the microtubule cytoskeleton inhibits receptor-activated release of Ca^2+^ via IP_3_Rs (Tasaka et al., 1991), prevents initiation of Ca^2+^ waves (Béliveau and Guillemette, 2009, Isshiki et al., 1998), slows diffusion of IP_3_Rs within ER membranes (Ferreri-Jacobia et al., 2005), disrupts local delivery of IP_3_ to IP_3_Rs (Graier et al., 1998, Ribeiro et al., 1997), and abolishes IP_3_-activated Ca^2+^ spikes at the apical pole of secretory cells (Fogarty et al., 2000). Many of these perturbations might result from effects of microtubules in organizing the ER, but there is also the possibility of more direct interactions with IP_3_Rs. The latter would be significant for vascular endothelial cells, where we have shown that PLC-evoked Ca^2+^ signals cause dephosphorylation of EB3 leading to persistent growth of microtubules, disassembly of AJs, and increased endothelial permeability (Komarova et al., 2012).

Here, we demonstrate a direct interaction between EB3 and the S/TxIP motif within IP_3_R3 that allows IP_3_Rs to associate with growing microtubule tips. Depletion of EB3 or disruption of the interaction with an IP_3_R3 point mutation prevents both clustering of IP_3_R3s and Ca^2+^ signals elicited by activation of PAR-1. Further, selective deletion of the EB3 gene in endothelium in mice inhibits the increase in endothelial permeability elicited by activation of PAR-1. Thus, microtubule-associated EB3 plays an obligatory role in organizing IP_3_-induced Ca^2+^ signaling, and, in turn, regulating endothelial permeability.

## Results

### Loss of EB3 Impairs Ca^2+^ Signaling in Endothelial Cells

**Figure 1:**
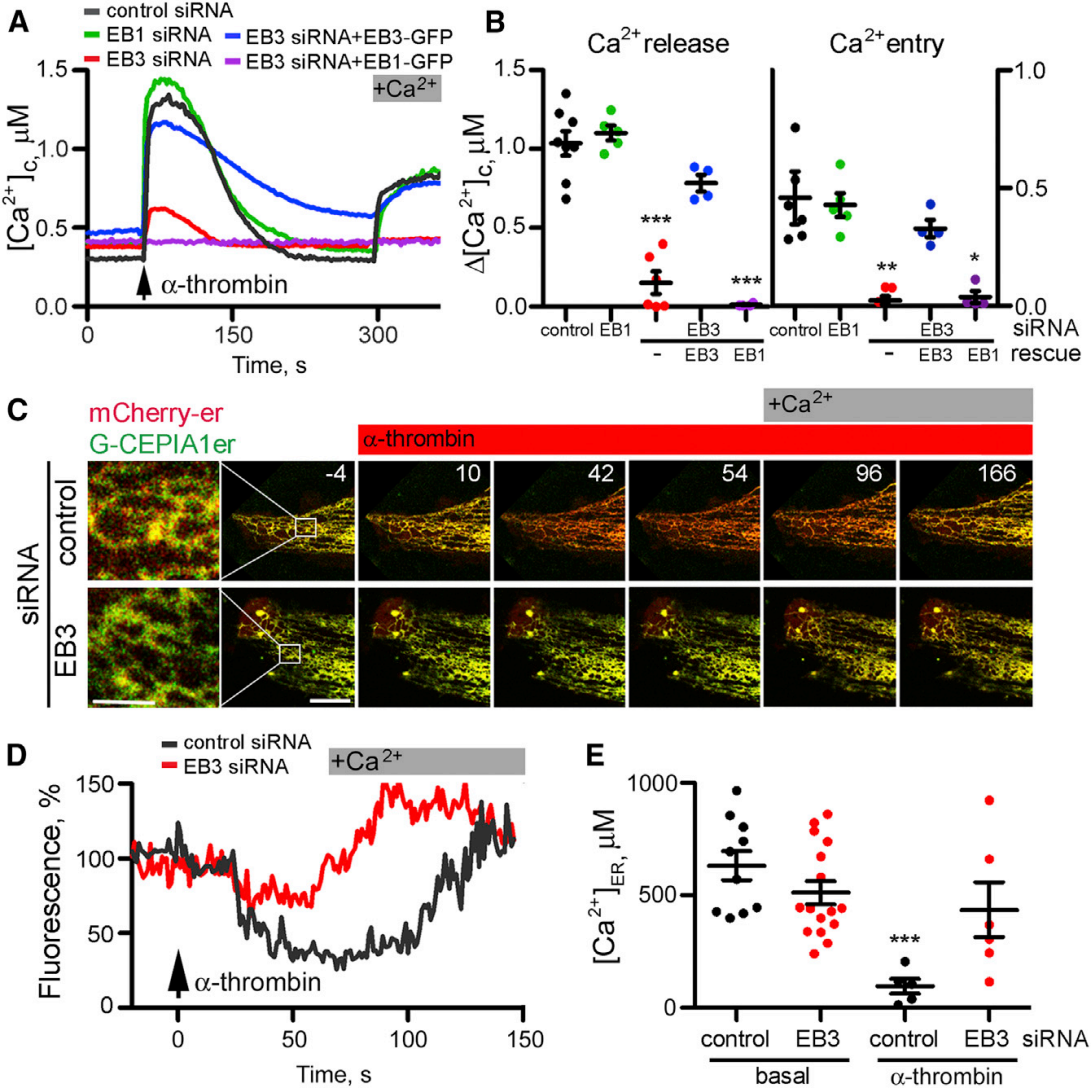
Depletion of EB3 Attenuates Receptor-Induced Ca^2+^ Release from ER (A) Cytosolic Ca^2+^ signals evoked by addition of α-thrombin (50 nM) recorded from ∼5–10 fura-2-loaded HLMVECs in Ca^2+^-free medium, followed by restoration of extracellular Ca^2+^ (2 mM). Cells were treated with siRNA for EB1 or EB3, control siRNA, or siRNA for EB3 together with expression of EB1-GFP or a siRNA-resistant EB3-GFP. (B) Summary results show peak changes in [Ca^2+^]_c_ (Δ[Ca^2+^]_c_) evoked by α-thrombin (Ca^2+^ release) and the subsequent restoration of extracellular Ca^2+^ (Ca^2+^ entry). Results show data points color coded as in (A), and mean ± SEM from four to eight experiments with five to ten cells analyzed in each group. ^∗,∗∗,∗∗∗^p values are relative to control siRNA-treated cells using one-way ANOVA. (C) Time-lapse images (times shown in seconds) show overlaid red (mCherry-ER) and green (G-CEPIA1er) fluorescence for cells treated with control or EB3 siRNA. Cells were stimulated with α-thrombin (50 nM at t = 0) in Ca^2+^-free medium, before restoration of extracellular Ca^2+^. Loss of luminal Ca^2+^ causes the green fluorescence of the luminal Ca^2+^ indicator to decrease, shifting images from yellow to red. The first panel shows an enlargement of the boxed area. Scale bars, 1 (enlargement) and 10 μm (other panels). (D) Experiments similar to those in (C) show the time course of the changes in [Ca^2+^]_ER_ presented as fluorescence ratios (G-CEPIA/mCherry-ER) normalized to the ratio recorded 4 s before addition of α-thrombin (100%) for cells treated with control or EB3 siRNA. (E) Summary results (mean ± SEM from 6 to 16 cells) show [Ca^2+^]_ER_ measured before (basal) and after stimulation with α-thrombin (50 nM) using GEM-CEPIA1er in cells treated with control or EB3 siRNA. Results are from two independent experiments; ^∗∗∗^relative to matched siRNA treatment without α-thrombin stimulation, using one-way ANOVA.

In primary cultures of human lung microvascular endothelial cells (HLMVECs), α-thrombin stimulates PAR-1, a GPCR that causes activation of PLCβ, formation of IP_3_ and release of Ca^2+^ from intracellular stores. Depletion of EB3, using small interfering RNA (siRNA) (Figure S1A), significantly reduced the amplitude of the Ca^2+^ signals evoked by addition of α-thrombin in Ca^2+^-free medium and the subsequent response to restoration of extracellular Ca^2+^ (Figures 1A and 1B). The inhibition was substantially reversed by expression of a siRNA-resistant EB3-GFP, but not by EB1-GFP. Loss of EB1 had no significant effect on α-thrombin-activated Ca^2+^ signals. A C-terminal fragment of EB3 (EB3-Ct-mRFP), which prevents binding of endogenous EBs to microtubule tips by forming non-functional dimers with endogenous proteins (Komarova et al., 2009), also inhibited α-thrombin-induced Ca^2+^ signals (Figures S1B and S1C).

We used low-affinity, genetically encoded Ca^2+^ indicators expressed in the lumen of the ER, G-CEPIA1er and the ratiometric indicator GEM-CEPIA1er (Suzuki et al., 2014), to establish whether loss of EB3 affected the Ca^2+^ content of the intracellular stores. Depletion of EB3 affected neither the organization of the ER nor the free Ca^2+^ concentration within the ER ([Ca^2+^]_ER_) (Figures 1C–1E). [Ca^2+^]_ER_ was 583 ± 77 μM and 489 ± 53 μM (n = 6–16 cells) in control and EB3 siRNA-treated cells, respectively (Figure 1E). Stimulation of control cells with α-thrombin caused [Ca^2+^]_ER_ to fall to 96 ± 33 μM, whereas α-thrombin had no significant effect on cells treated with siRNA for EB3 (436 ± 122 μM) (Figure 1E). Furthermore, refilling of ER after restoration of extracellular Ca^2+^ to cells stimulated with α-thrombin in Ca^2+^-free medium was faster in cells lacking EB3 (Figure 1D), consistent with reduced activation of IP_3_Rs after knockdown of EB3. These results demonstrate that loss of EB3 attenuates the release of Ca^2+^ from intracellular stores elicited by α-thrombin without affecting the initial Ca^2+^ content of the ER.

### EB3 Binds to IP_3_Rs

To assess whether the effects of EB3 on Ca^2+^ release involved reorganization of the microtubule network, we analyzed changes in microtubule dynamics after addition of α-thrombin using time-lapse confocal imaging of EB1-GFP to mark growing microtubule tips. We chose EB1-GFP for this analysis because it does not rescue the inhibition of α-thrombin-evoked Ca^2+^ signals after EB3 depletion, and nor does loss of endogenous EB1 affect Ca^2+^ signals (Figures 1A and 1B). In confluent monolayers of HLMVECs treated with control siRNA, microtubules grew at 13.7 ± 3.1 μm/min, and they displayed frequent catastrophe events. Depletion of EB3 had no effect on the growth rate or catastrophe frequency (Table 1), suggesting that EB3 did not affect microtubule dynamics under basal conditions. This finding is consistent with previous work demonstrating that most EB3 is phosphorylated in unstimulated HLMVECs, and therefore unable to promote persistent growth of microtubules (Komarova et al., 2012). In the 2–3 min after stimulation of PAR-1 with α-thrombin, there was no effect on the catastrophe frequency, but the stimulation unexpectedly reduced microtubule growth rate in both control and EB3-siRNA-treated cells (Table 1; Figure S2). These results demonstrate that loss of EB3 has no discernible effect on microtubule dynamics under conditions where it attenuates α-thrombin-evoked Ca^2+^ release. We therefore considered whether EB3 might regulate PLC or IP_3_Rs.

We used the IP_3_ biosensor, LIBRAvIII, in which IP_3_ binding causes a decrease in intramolecular fluorescence resonance energy transfer (FRET) (Tanimura et al., 2009), to measure cytosolic IP_3_ concentrations in confluent monolayers of HLMVECs. α-Thrombin caused a decrease in the FRET signal consistent with an increase in cytosolic IP_3_ concentration. The response was similar in cells treated with control siRNA or siRNA to EB3 (Figure S3A). These results thus suggest that under conditions where loss of EB3 attenuates α-thrombin-evoked Ca^2+^ signals, EB3 has no effect on α-thrombin-induced IP_3_ formation.

**Figure 2:**
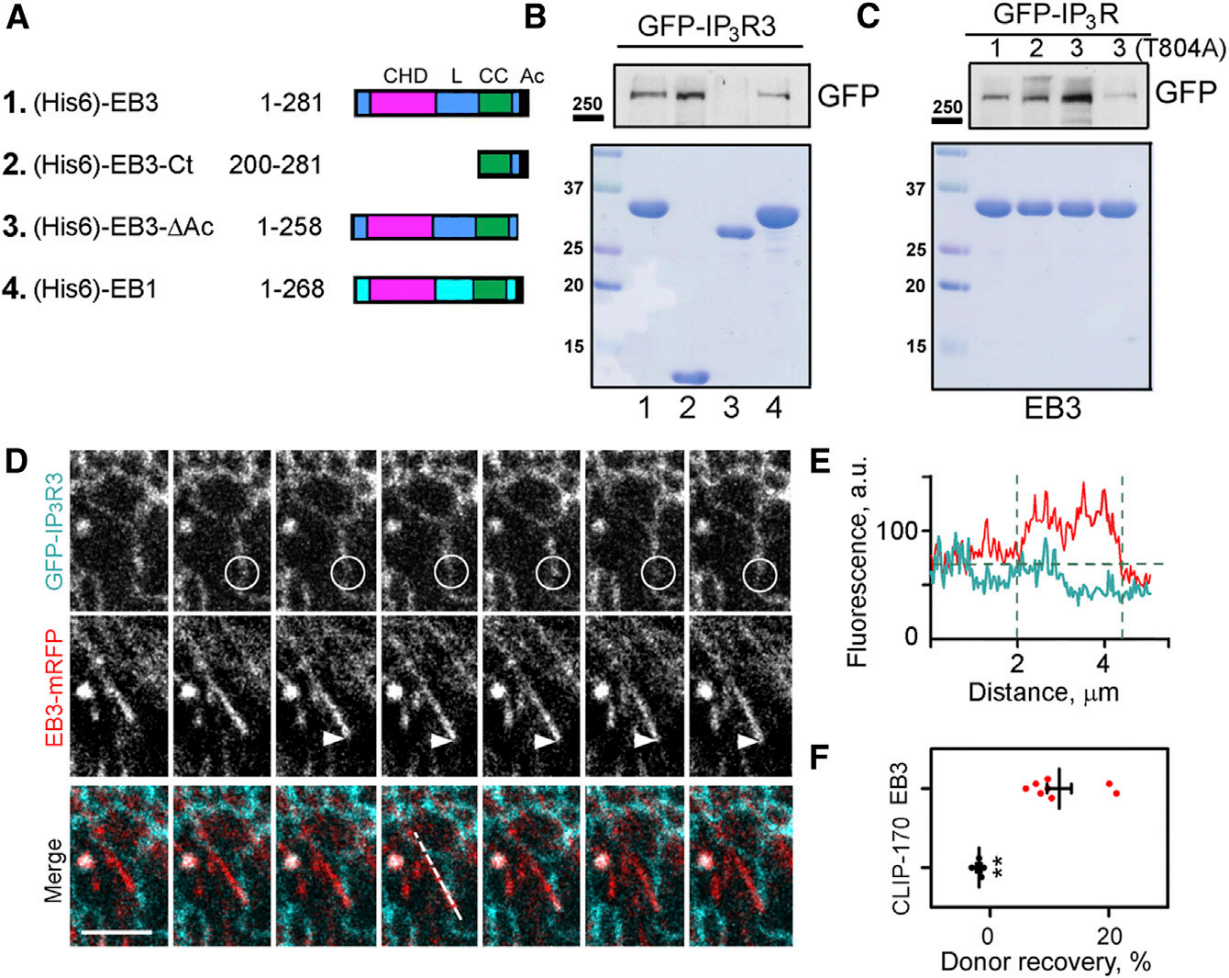
EB3 Interacts Directly with IP_3_Rs (A) Schematic representation of hexa-histidine (His6)-tagged EB constructs used for pull-down experiments. CHD, calponin homology domain; L, linker; CC, coiled coil; Ac, acidic tail. (B and C) Pull-down analyses of interactions between (His6)-EB proteins covalently bound to Ni-NTA resin and lysates from HEK cells expressing GFP-IP_3_R1-3 or GFP-IP_3_R3(T804A). Upper panels show western blots for GFP and lower panels show Coomassie brilliant blue-stained gels loaded with 5% of the EBs (numbered as in A) used for the pull-down. Results are typical of three independent experiments. Western blots of GFP-IP_3_Rs in the cell lysates used and additional controls are shown in Figure S3. (D) Confocal images collected at 850-ms intervals show simultaneous recordings of fluorescence from EB3-mRFP and GFP-IP_3_R3 in HLMVECs. The composite panels show overlaid GFP-IP_3_R3 (green) and EB3-mRFP (red). Note the loss of GFP-IP_3_R3 fluorescence (circle) as the EB3-mRFP-labeled microtubule tip approaches (arrow). Scale bar, 5 μm. (E) EB3-mRFP and GFP-IP_3_R3 fluorescence recorded along the dashed line shown in the merged images in (D) illustrates the decrease in GFP fluorescence at the point of interaction with the microtubule tip. (F) Focal photobleaching of the acceptor fluorophore (mRFP) at the microtubule tip while recording donor fluorescence from GFP-IP_3_R was used to assess the interaction between GFP-IP_3_R3 and EB3-mRFP or CLIP-170-mRFP at the microtubule tip. Individual data points with mean ± SEM from five to eight cells analyzed in each group show the recovery of the donor fluorescence after acceptor photobleaching (%). ^∗∗^Using Student’s t test.

Alignment of IP_3_R sequences identified a conserved SxIP motif within a short region of disordered protein structure in all three mammalian IP_3_R subtypes (Figure S3B). This motif (residues 804–807 in human IP_3_R3), a signature of EB-interacting partners (Honnappa et al., 2009), is located downstream of the IP_3_-binding site. The interaction between full-length EB3 or its C-terminal region (residues 200–281), and IP_3_R3 was demonstrated in pull-down assays using immobilized (His6)-EBs expressed in bacteria and GFP-IP_3_Rs from lysates of HEK cells (Figures 2A, 2B, and S3C). IP_3_R1 and IP_3_R2 also associated with full-length EB3 (Figure 2C). EB1 was less effective than EB3 in the pull-downs of GFP-IP_3_R3 (Figure 2B). For each IP_3_R subtype, the interaction with EB3 was weaker than that between EB3 and STIM1, another Ca^2+^ signaling protein to which EB1 and EB3 bind (Grigoriev et al., 2008) (Figure S3D). Interaction between endogenous EB3 and IP_3_R3 was confirmed by their co-immunoprecipitation (Figure S3E). Deletion of the acidic C-terminal tail of EB3 (EB3ΔAc), which contributes to the binding interface for the S/TxIP motif, abolished the interaction of IP_3_R3 with EB3 (Figure 2B). Mutation of the critical Thr within the TxIP motif of IP_3_R3 (T804A) also abolished the interaction with EB3 (Figure 2C). These results suggest a direct interaction between the C-terminal region of EB3 and the TxIP motif of IP_3_R3, an interaction that is probably shared with other IP_3_R subtypes.

In HLMVECs expressing EB3-mRFP and GFP-IP_3_R3, there were transient contacts between growing microtubule tips and GFP-IP_3_R3 in ER membranes (Figure 2D). However, GFP-IP_3_R3 did not form the “comet-like” structures described for STIM-1 associated with growing microtubule tips (Grigoriev et al., 2008, Pozo-Guisado et al., 2010). This finding is consistent with the lower affinity of EB3 relative to STIM1 for IP_3_R3, because comets reveal the density of EB proteins, which declines with distance from the microtubule tip. We also observed quenching of the GFP fluorescence when EB3-mRFP-labeled microtubule tips made contact with ER tubules, suggesting an intermolecular FRET between EB3-mRFP and GFP-IP_3_R3 (Figures 2D and 2E). Focal photobleaching of the EB3-mRFP acceptor caused a transient increase in closely apposed fluorescence of the GFP-IP_3_R3 donor (Figure 2F), confirming an interaction between EB3 and IP_3_R3 in intact cells. There was no detectable FRET between GFP-IP_3_R3 and another +TIP, mRFP-CLIP-170 (Figure 2F). The results thus indicate a specific interaction between EB3 and IP_3_R3 in intact human lung endothelial cells.

### Interactions between EB3 and IP_3_R3 Regulate IP_3_R3 Dynamic and Activity

**Figure 3:**
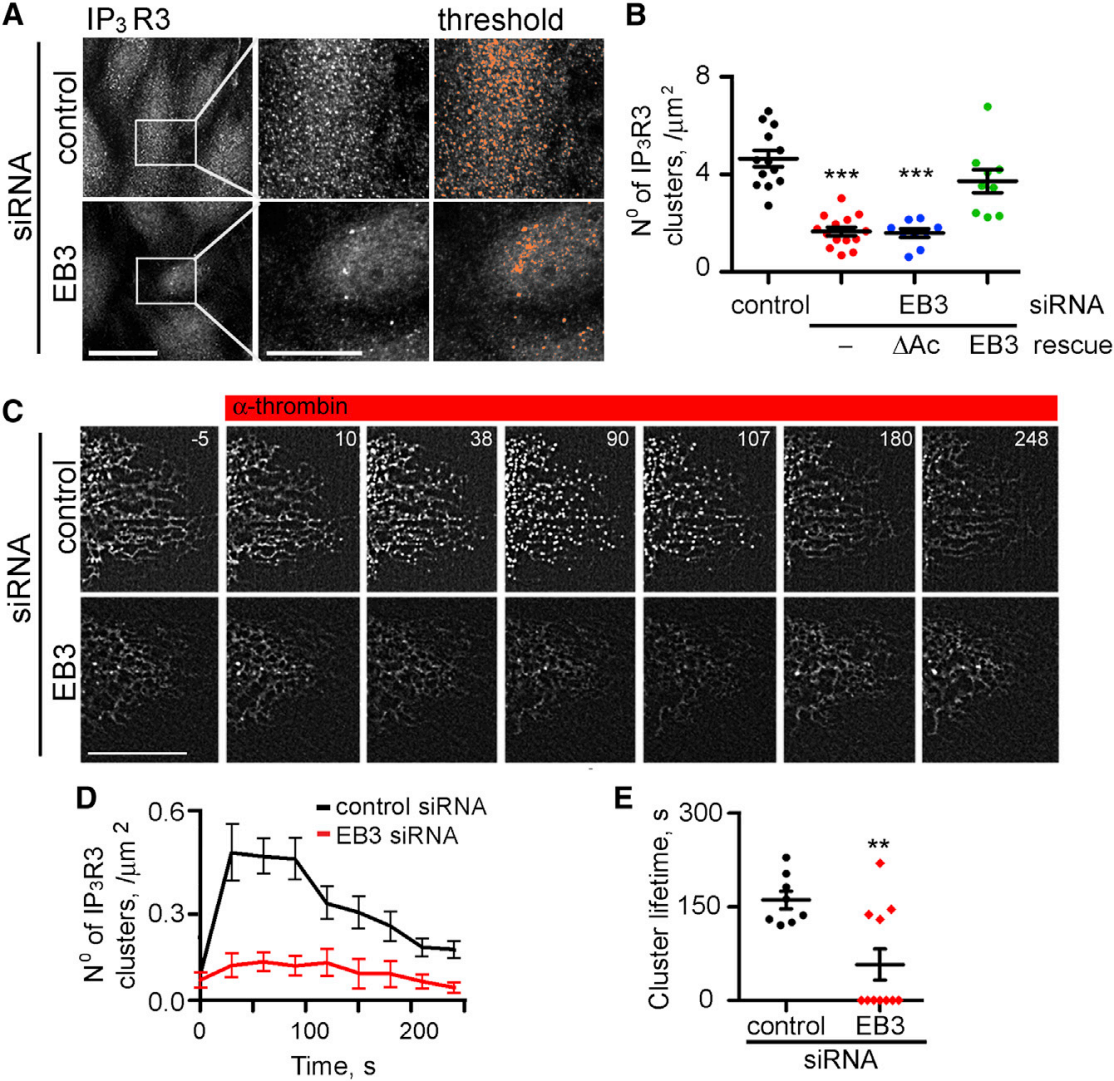
EB3 Facilitates Clustering of IP_3_R3 (A) Intracellular distribution of endogenous IP_3_R3 (immunostaining) in HLMVECs treated with control or EB3 siRNA. Central panels show enlargements of the boxed area. The thresholded images used to measure cluster densities (see Experimental Procedures) are shown in the right panels. Scale bar, 10 μm. (B) Summary results from nine to 14 cells show numbers of IP_3_R3 clusters in cells treated with control or EB3 siRNA alone or after rescue with EB3-GFP or EB3ΔAc-GFP. Individual data points and mean ± SEM are shown. ^∗∗∗^Compared to control siRNA-treated cells using one-way ANOVA. (C) Time-lapse images (collected at 0.5-s intervals) of GFP-IP_3_R3 expressed in HLMVECs treated with control or EB3 siRNA and stimulated with α-thrombin (50 nM, as indicated). Times (s) are shown in each panel. Scale bar, 10 μm. (D and E) Summary results (mean ± SEM from 11 cells in each group) show time course of GFP-IP_3_R3 clustering after addition of α-thrombin (at t = 0) and (E) the lifetime of the clusters (time taken for 90% of clusters to disappear). These analyses were performed using processed images in which clusters present before addition of α-thrombin were subtracted (see Experimental Procedures). ^∗∗^Using Student’s t test. Depletion of EB3 reduced both the number and lifetime of the IP_3_R3 clusters. Figure S4 shows additional control experiments and related analyses of GFP-IP_3_R2.

Analysis of mRNA and protein expression demonstrated that in various human pulmonary endothelial cells, including HLMVECs, IP_3_R2 and IP_3_R3 were the major subtypes (Figures S4A–S4C). Immunostaining of HLMVECs revealed that IP_3_R3 formed puncta (Figure 3A), consistent with their assembly into clusters, as reported for IP_3_Rs in endothelial cells (Tran et al., 2014). Depletion of EB3 had no effect on the expression of IP_3_R3 (Figure S4D), but it significantly reduced the number of IP_3_R3 clusters (Figures 3A and 3B). EB3 depletion had no effect on IP_3_R2 clusters, which were observed in the perinuclear region in both control and EB3 siRNA-treated cells (Figures S4E and S4F). The inhibition of IP_3_R3 clustering by depletion of EB3 was reversed by expression of a siRNA-resistant EB3, but not by expression of the EB3ΔAc mutant (Figure 3B) that does not bind to IP_3_R3 (Figure 2B).

GFP-IP_3_R3 also formed clusters in unstimulated HLMVECs, with fewer clusters in cells lacking EB3 (Figure 3C). This allowed dynamic imaging of GFP-IP_3_R3 distribution in response to receptor activation. Stimulation of HLMVECs expressing GFP-IP_3_R3 with α-thrombin evoked a rapid transient increase in IP_3_R3 clustering that peaked after ∼30 s and persisted for 190 ± 15 s (time for 90% of clusters to disappear) (Figures 3C–3E). Similar clustering of IP_3_Rs in response to stimuli that caused IP_3_R activation has been reported in other cells (Taufiq-Ur-Rahman et al., 2009, Tateishi et al., 2005). Both the number of IP_3_R3 clusters after stimulation with α-thrombin and their lifespan (64 ± 28 s) were reduced in EB3-depleted HLMVECs (Figures 3D and 3E).

**Figure 4:**
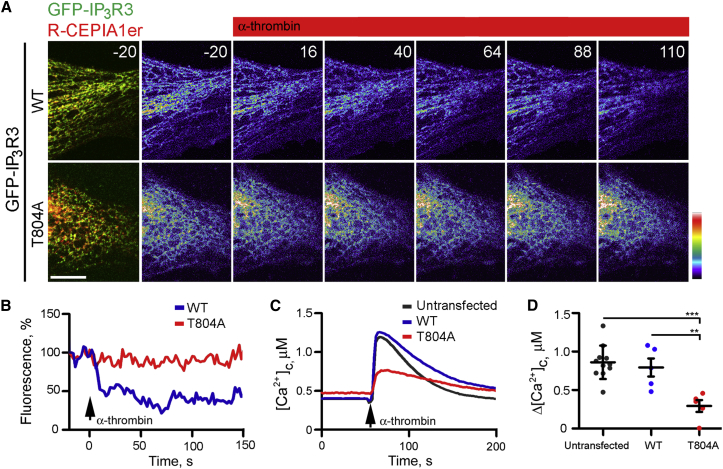
Interaction between IP_3_R3 and EB3 Is Required for α-Thrombin-Induced Ca^2+^ Signaling (A) Time-lapse images (times, in s, shown in panels) of HLMVEC expressing GFP-IP_3_R3 (WT) or GFP-IP_3_R3(T804A) mutant (green) with the ER luminal Ca^2+^ indicator, R-CEPIA1er (red) in overlaid images. R-CEPIA1er fluorescence is color coded with warm colors denoting high [Ca^2+^]_ER_. Stimulation with α-thrombin (50 nM at t = 0) in Ca^2+^-free medium caused a decrease in [Ca^2+^]_ER_ in cells expressing wild-type (WT) GFP-IP_3_R3, but not in cells expressing GFP-IP_3_R3(T804A). Scale bar, 10 μm. (B) Representative traces (normalized to 100% at 20 s before addition of α-thrombin) show [Ca^2+^]_ER_ monitored with R-CEPIA1er in individual cells expressing GFP-IP_3_R3 or GFP-IP_3_R3(T804A) and stimulated with α-thrombin (50 nM) in Ca^2+^-free medium. (C) Effects of α-thrombin (50 nM) on [Ca^2+^]_c_ recorded from ∼5–10 fura-2-loaded HLMVECs expressing GFP-IP_3_R3 (WT) or GFP-IP_3_R3(T804A), or adjacent untransfected cells. (D) Summary results show peak changes in [Ca^2+^]_c_ (Δ[Ca^2+^]_c_) evoked by α-thrombin. Individual data points (four to ten cells) and means ± SEM are plotted. ^∗∗∗^, ^∗∗^Using one-way ANOVA.

**Figure 5:**
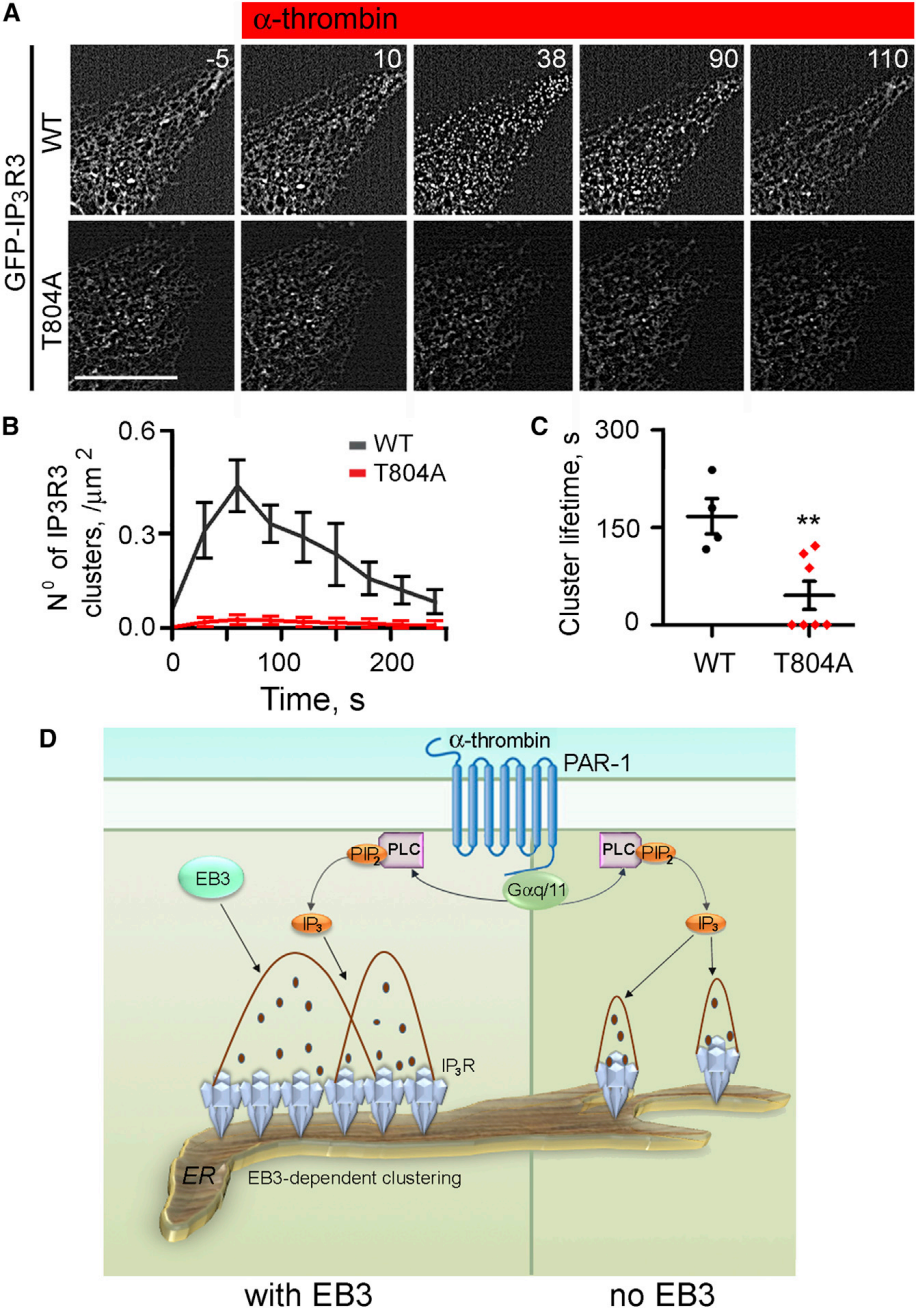
Interaction between IP_3_R3 and EB3 Is Required for Effective IP_3_R3 Clustering (A) Time-lapse images (times, in s, shown in panels) of GFP fluorescence in HLMVECs expressing GFP-IP_3_R3(WT) or GFP-IP_3_R3(T804A) after stimulation with α-thrombin (50 nM, at t = 0). Scale bar, 10 μm. (B and C) Summary results (mean ± SEM from five to seven cells) show the number of clusters for GFP-IP_3_R3 and GFP-IP_3_R3(T804A) (B) and cluster lifetime (C) after stimulation with α-thrombin (50 nM at t = 0). Data were analyzed using processed images as in Figures 3D and 3E. ^∗∗^Using Student’s t test. (D) Model for EB3-dependent IP_3_R3 clustering and amplification of Ca^2+^ signals. EB3 facilitates clustering of IP_3_Rs within ER membranes (left). α-Thrombin, via PAR-1, activates PLC and synthesis of IP_3_. We propose that IP_3_Rs more effectively release Ca^2+^ in response to this IP_3_ when they have clustered, possibly because amplification of the signals by Ca^2+^-induced Ca^2+^ release is more effective.

Residue T804 within the TEIP motif of IP_3_R3 was required for IP_3_R3 binding to EB3 (Figure 2C). Expression of GFP-IP_3_R3(T804A) in HLMVECs attenuated α-thrombin-evoked Ca^2+^ signals. This was evident from the smaller effects of α-thrombin on both the decrease in [Ca^2+^]_ER_ and increase in [Ca^2+^]_c_ in cells expressing GFP-IP_3_R3(T804A) relative to those expressing GFP-IP_3_R3 (Figure 4). GFP-IP_3_R3(T804A) also formed fewer clusters than GFP-IP_3_R3, they barely responded to α-thrombin, and the few clusters that formed were short lived (Figures 5A–5C). We conclude that EB3, via its interaction with IP_3_R3 in endothelial cells, both dynamically regulates basal and agonist-evoked clustering of IP_3_R3 and the IP_3_-mediated Ca^2+^ release induced by α-thrombin (Figure 5D).

### Loss of EB3 Suppresses Vascular Leakage In Vivo

**Figure 6:**
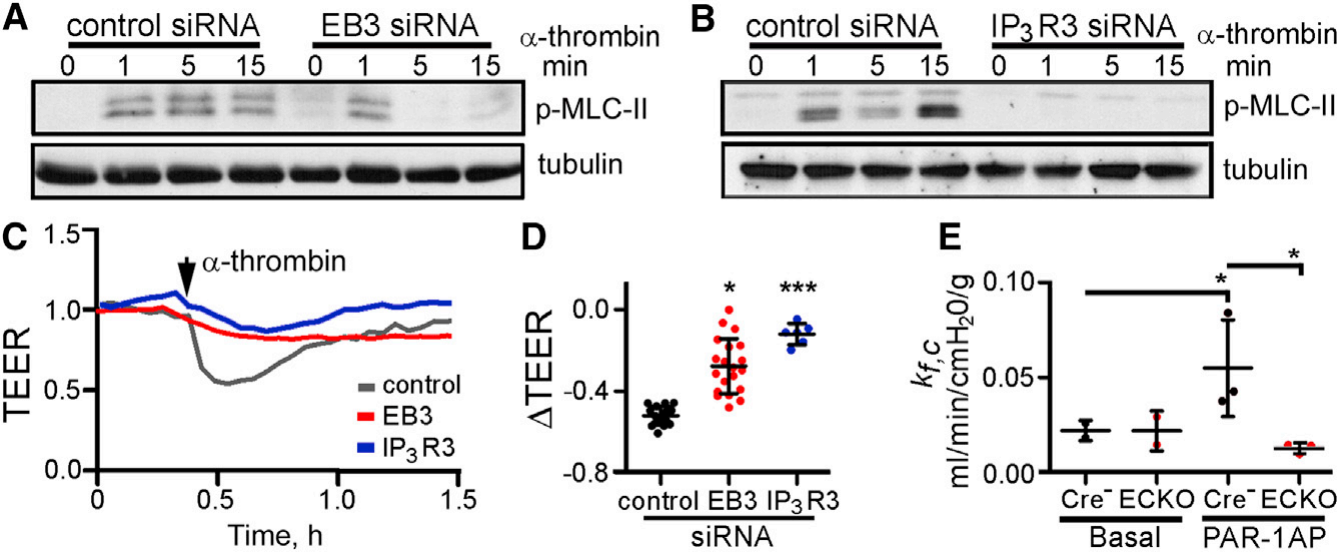
Depletion of EB3 Attenuates α-Thrombin-Induced Phosphorylation of MLC-II and Endothelial Hyper-permeability (A and B) Time course of MLC-II phosphorylation in HPAECs treated with the indicated siRNAs and stimulated with α-thrombin (50 nM at t = 0). The western blots are typical of two to three experiments. (C) Typical effects of α-thrombin (50 nM) on TEER in HPAECs treated with the indicated siRNAs. Basal resistance was normalized to 1. (D) Summary results show the maximum change in TEER (ΔTEER) evoked by α-thrombin. Individual data points with mean ± SEM are shown, n = 6–20 per group, ^∗^, ^∗∗∗^Compared to control siRNA-treated cells using one-way ANOVA. (E) Permeability of endothelial vessel wall in lungs from EB3-*iECKO* and Cre-negative mice assessed by measuring microvascular filtration coefficient, *k*_*f,c*_ (see Experimental Procedures). Isolated lungs were infused with 30 μM PAR-1 agonist peptide (PAR1-AP), and *k*_*f,c*_ was calculated from the slope of the weight-gain curve between 15 and 20 min after the infusion. Individual data points are shown with mean ± SD, n = 3 mice per group; ^∗^From ANOVA. See also Figure S5.

Ca^2+^ signals, via both activation of protein kinase C α and myosin light chain (MLC) kinase, facilitate cell contraction and destabilization of AJs (Komarova and Malik, 2010, Vandenbroucke St Amant et al., 2012). We therefore examined the effects of EB3 and IP_3_R3 on phosphorylation of MLC-II. We used human pulmonary artery endothelial cells (HPAECs) for these analyses because they are amenable to measurements of trans-endothelial electrical resistance (TEER), which directly report, in real time, changes in the integrity of AJs (Vandenbroucke St Amant et al., 2012, Szulcek et al., 2013). Depletion of EB3 or IP_3_R3 (Figure S4C) suppressed α-thrombin-induced phosphorylation of MLC-II in HPAECs (Figures 6A and 6B). In confluent monolayers of HPAECs, α-thrombin induced a decrease in TEER, reflecting changes in cell shape and disruption of AJs between endothelial cells. The decrease in TEER was attenuated in cells lacking IP_3_R3 or EB3 (Figures 6C and 6D), consistent with the lesser α-thrombin-evoked phosphorylation of MLC-II in these cells. These findings suggest that the interactions between EB3 and IP_3_R3, through their effects on α-thrombin-induced Ca^2+^ signals, contribute to MLC-II activation and the increased permeability of the endothelial barrier.

Ca^2+^ signals in the endothelium play a critical role in regulating vascular permeability, a hallmark of acute lung injury and inflammation (Gandhirajan et al., 2013, Tauseef et al., 2012). To determine the role of EB3 in vascular endothelium, we generated EB3-*iECKO* mice in which high-fidelity inducible deletion of the EB3 gene (*Mapre3*) was restricted to endothelial cells (Figure S5). We then used lungs to determine the microvessel filtration coefficient (*k*_*f,c*_), a measure of endothelial vascular permeability to liquid, in naive lungs and after activation of PAR-1 (Figure 6E). Lungs from control *Tie2*-*Cre*ER^T2^-negative and EB3-*iECKO* mice had similar basal permeability, suggesting that EB3 is not essential in the adult microvasculature (Figure 6E). Infusion of a peptide agonist of PAR-1 (PAR1-AP) caused lung vascular hyper-permeability in wild-type, but not in EB3-*iECKO* mice (Figure 6E), indicating a pivotal role of EB3 in mediating the lung vascular permeability response.

## Discussion

Here, we show that stimulation of PLCβ as induced by the inflammatory mediator α-thrombin causes IP_3_R3s to assemble into clusters in the ER membrane of endothelial cells and that this event requires the association of the TxIP motif of IP_3_R3 with EB3 located at the tip of growing microtubules. Disrupting this interaction inhibits Ca^2+^ signaling, the phosphorylation of MLC-II, and the subsequent increase in endothelial permeability in response to α-thrombin.

The local density of IP_3_Rs in the ER membrane induced by IP_3_R clustering is thought to be responsible for the spatially organized nature of Ca^2+^ signaling in distinct cellular domains (Isshiki et al., 1998, Low et al., 2010). The spacing of IP_3_Rs is key in determining whether the Ca^2+^ released stimulates the activity of neighboring IP_3_Rs, and hence propagates Ca^2+^ signaling in a regenerative manner. Optimal clustering of IP_3_Rs is therefore believed to be a critical factor in Ca^2+^ signaling (Shuai and Jung, 2003). A major factor determining IP_3_R clustering may be IP_3_-evoked conformational changes in IP_3_Rs (Taufiq-Ur-Rahman et al., 2009, Tateishi et al., 2005). Studies also showed that microtubules facilitated movement of IP_3_Rs in cells (Ferreri-Jacobia et al., 2005), and further that disruption of microtubules inhibited IP_3_-evoked Ca^2+^ spiking (Béliveau and Guillemette, 2009, Fogarty et al., 2000), suggesting a role of microtubules in the mechanism of IP_3_R clustering. However, Taufiq-Ur-Rahman et al. (2009) showed that clustering of IP_3_Rs within isolated nuclei did not require microtubules. Thus, it has not been resolved whether microtubules regulate ER dynamics, and, if so, by what mechanisms and whether microtubule regulation of IP_3_R clustering has functional relevance in an important Ca^2+^-regulated biological response. Our results show the direct interaction of IP_3_Rs with microtubules via the microtubule tip protein EB3 is required for the assembly of IP_3_Rs into signaling clusters at the ER membrane, and these clusters mediate Ca^2+^ release from ER stores in endothelial cells. Our previous work established that IP_3_-induced Ca^2+^ release in endothelial cells activated the phosphatase calcineurin, which dephosphorylated EB3, enabling EB3 dimerization. EB3 dimers, in turn, bound microtubules to promote persistent growth and disassembly of AJs (Komarova et al., 2012). The present work demonstrates that the microtubules functioning via EB3 are required for IP_3_R clustering at the ER membrane and the genesis of intracellular Ca^2+^ signaling in endothelial cells.

The S/TxIP motif, located immediately downstream of the IP_3_-binding site and conserved in all mammalian IP_3_Rs, was required for IP_3_Rs binding specifically to the C-terminal tail of EB3 (as opposed to EB1) in endothelial cells. The enhanced binding of EB3 to IP_3_R3 correlated with the requirement for EB3, rather than EB1, for α-thrombin-induced Ca^2+^ signaling. The requirement for EB3 may also result from its binding to additional, as-yet-unidentified proteins mediating clustering of IP_3_R3s. Indeed, residues surrounding the S/TxIP motif have been recently shown to recognize EB3 (Leśniewska et al., 2014). Inhibition of α-thrombin-induced Ca^2+^ signals by a fragment of EB3 (Ct-mRFP) that dimerizes with native EB3 and prevents binding to microtubules confirmed that the requirement for EB3 is associated with its ability to bind to the growing microtubule tips. The concept of EB3 binding to IP_3_R3 is further supported by our FRET analysis showing a close and specific apposition of IP_3_R3 and EB3 at microtubule tips.

We found that IP_3_R3 and IP_3_R2 are the major IP_3_R subtypes in endothelial cells. IP_3_R2s are concentrated in perinuclear regions and clustered in unstimulated endothelial cells. The perinuclear distribution of IP_3_R2 (Pantazaka and Taylor, 2011) and their propensity to cluster in unstimulated cells are also observed in other cells (Iwai et al., 2005, Sheppard et al., 1997). The latter finding suggests that basal levels of IP_3_ are sufficient to stimulate IP_3_R2 clustering, consistent with the greater affinity of IP_3_R2 for IP_3_ relative to other subtypes (Iwai et al., 2007). In unstimulated endothelial cells, however, IP_3_R3s are more widely distributed and less clustered than IP_3_R2s. The key role of IP_3_R3 in mediating α-thrombin-induced Ca^2+^ signals is therefore probably due to their proximity to IP_3_ produced by PLC at the plasma membrane. Our observation that α-thrombin augments the clustering of IP_3_R3s is consistent with their lower sensitivity to IP_3_ (Iwai et al., 2007). IP_3_-induced clustering of IP_3_R3s in the nuclear envelope did not require microtubules (Taufiq-Ur-Rahman et al., 2009), and yet in endothelial cells clustering requires EB3-mediated interaction of IP_3_R3 with microtubules. The discrepancy may reflect a need for microtubules to facilitate movement of IP_3_Rs within the crowded cytoplasm of intact cells and stabilize IP_3_R clusters once they have formed.

We conclude that EB3-mediated tethering of IP_3_R3s to microtubule tips in endothelial cells is required to assemble the Ca^2+^ signaling machinery. Moreover, α-thrombin stimulates formation of IP_3_ at the plasma membrane, where it facilitates the clustering of IP_3_R3 via EB3-mediated interaction with microtubules. We propose that IP_3_R3 clustering enables Ca^2+^-mediated amplification of IP_3_-induced Ca^2+^ release through activation of neighboring IP_3_Rs. The IP_3_-evoked Ca^2+^ signaling can therefore be attenuated in the absence of this amplification step. The physiological importance of the EB3-IP_3_R3 interaction is evident from results in lung microvessels showing that endothelial cell-specific knockout of EB3 prevents the increase in endothelial permeability in response to inflammatory signal. Thus, microtubule-associated EB3 interacts directly with IP_3_Rs to assemble the Ca^2+^ signaling complex at the ER membrane, and disruption of EB3-IP_3_R interaction may be an attractive therapeutic target for vascular inflammation.

## Experimental Procedures

### Materials

Sources of the expression constructs, antibodies, and siRNAs are provided in Supplemental Experimental Procedures. mRFP-(rat)CLIP-170 was generated from EGFP-CLIP-170 (Komarova et al., 2005) by substituting EGFP with monomeric RFP. For EB3-Ct-mRFP, the C terminus (residues 200–281) of EB3 was amplified by PCR and sub-cloned into the pmRFP-N1 vector (a gift from Dr. R. Tsien) at Sal1 and BamH1 sites. Expression constructs for the siRNA-insensitive form of EB3 and for EGFP-IP_3_R3(T804A) were generated using the QuikChange Site-Directed Mutagenesis Kit (Agilent Technologies).

DAPI was from Sigma. Human α-thrombin was from Fisher Scientific. The PAR-1-activating peptide (PAR1-AP, TFLLRN-NH_2_, ∼90% purity) was synthesized by the Research Resources Core at UIC. Sources of other materials are provided in the relevant sections in Experimental Procedures.

### Pull-Down Assays with EB Proteins

Preparation of bacterially expressed (His6)-tagged EB proteins and their covalent immobilization on Ni-NTA columns for pull-down experiments using lysates from HEK cells expressing GFP-tagged proteins are described in Supplemental Experimental Procedures.

### Cell Culture and Transfections

HPAECs and HLMVECs (Lonza) were grown in EGM-2 medium supplemented with 10% fetal bovine serum (FBS) and EGM-2 MV Bulletkit or EGM-2 Bulletkit (Lonza), respectively. Cells were used between passages 2 and 6. CHO-K1 and HEK293 cells (ATCC) were grown in DMEM with 10% FBS (Gibco). Cells were transfected at ∼80% confluence using X-tremeGENE HP according to the manufacturer’s protocol (Roche) and used after 24–48 hr. For siRNA-mediated inhibition of protein expression, cells were treated with 70 nM siRNA (Supplemental Experimental Procedures) using GeneSilencer transfection reagent according to the manufacturer’s protocol (Genlantis) and used after 72–96 hr. For experiments in which cells were stimulated with α-thrombin, the FBS concentration of the culture medium was reduced to 0.5% for 1 hr before the experiment.

### Measurements of [Ca^2+^]_c_

HLMVECs grown on glass-bottomed dishes (Becton Dickinson) were loaded with fura-2 AM (3 μM, Life Technologies) for 20 min at 37°C in culture medium without supplements. The medium was then replaced with medium comprising: 150 mM NaCl, 4 mM KCl, 1 mM MgCl_2_, 5.6 mM glucose, and 25 mM HEPES (pH 7.4), and, after ∼10 min, cells were used for experiments at 25°C. Fura-2 fluorescence was excited at 340 and 380 nm and collected at 510 ± 80 nm using an Axiovert 100 inverted microscope (Carl Zeiss) equipped with Plan-Apo 60× with the numerical aperture (NA) 1.4 oil immersion objective, Lambda DG-4 switcher illumination system (Sutter Instruments), AxioCom Hsm camera (Zeiss), fura-2 filter set (Chroma), and AxioVision Physiology Acquisition module. Images were collected at 2-s intervals. Fluorescence ratios (F_340_/F_380_) were calculated within a circular region of interest (radius 3 μm) for each cell after subtraction of intracellular background fluorescence, determined by quenching fura-2 fluorescence by addition of 3 μM ionomycin with 5 mM MnCl_2_. [Ca^2+^]_c_ was calculated from F_340_/F_380_ ratios by reference to Ca^2+^ standard solutions (Life Technologies). Measurements of cytosolic IP_3_ concentrations are described in Supplemental Experimental Procedures.

### Measurements of [Ca^2+^]_ER_

The free [Ca^2+^] within the ER ([Ca^2+^]_ER_) was measured in HLMVECs transfected with the GEM-CEPIA1er ratiometric indicator (Suzuki et al., 2014). Analyses were performed at 37°C in the Ca^2+^-free medium described above using a confocal microscope (Zeiss LSM 710, Axio Observer Z1) equipped with a 63× 1.4 NA Plan-Apochromat oil immersion objective and a diode-pumped solid-state laser. Cells were excited at 405 nm, and emission was collected by two photomultiplier tubes (456–476 and 510–530 nm). Images (5–10/cell) were collected at 1-s intervals, averaged over four frames to optimize signal-to-noise ratios, and fluorescence ratios (R = F_466_/F_510_) were calculated. For calibration, the plasma membrane was permeabilized (150 μM β-escin, 4 min), R_min_ and R_max_ were then determined in the absence of β-escin in medium with (R_max_) or without (R_min_) Ca^2+^. The medium comprised: 140 mM KCl, 10 mM NaCl, 1 mM MgCl_2_, 20 mM HEPES, 3 μM ionomycin, and 3 μM thapsigargin (pH 7.4), and either 0.3 mM EGTA (R_min_) or 10 mM CaCl_2_ (R_max_). [Ca^2+^]_ER_ was then calculated from the observed fluorescence ratio (R):

$${\left[Ca^{2+}\right]}_{ER}=\;K_{D}{\left(\frac{R\;-\;R_{min}}{R_{max}\;-\;R}\right)}^{\frac{1}{n}},$$

where n, Hill coefficient (1.37) and K_D_ for Ca^2+^ = 558 μM (Suzuki et al., 2014).

A confocal microscope (Zeiss, LSM 710, Axio Observer Z1) with BIG detector was used to capture dual-color images of m-Cherry-er with G-CEPIAer. For comparisons of relative changes in [Ca^2+^]_ER_ (Figure 1D), a cross-sectional view of the intensity values of G-CEPIAer fluorescence over time was generated, and the relative changes in [Ca^2+^]_ER_ were calculated.

### Immunofluorescence and Live-Cell Imaging

HLMVECs were fixed with 4% formaldehyde, permeabilized with 0.2% Triton X-100, and immunostained for IP_3_R2 or IP_3_R3 (Supplemental Experimental Procedures). All z stack confocal images (Zeiss LSM 510 META) were acquired with the same settings to allow comparisons of IP_3_R distributions between treatment groups. A projected image was generated by collecting a maximum voxel value through each z stack.

The methods used to quantify microtubule dynamics using EB1-GFP, assess the interactions between GFP-IP_3_R3 and EB3-mRFP or mRFP-CLIP-170 using acceptor photobleaching, and to quantify clustering of GFP-IP_3_R3 or immunostained IP_3_R2 and IP_3_R3 are described in Supplemental Experimental Procedures.

### Trans-endothelial Electric Resistance Measurements

HPAECs were plated onto gelatin-coated 8W1E gold electrodes (Applied Biophysics), transfected with siRNA, and used after 72 hr (Garcia et al., 2011). Changes in trans-endothelial electric resistance (TEER) in response to α-thrombin were monitored using an Electric Cell Substrate Impedance Sensing system (Applied Biophysics) and normalized to the basal resistance.

### Measurement of Vessel Filtration Coefficient (*k*_*fc*_)

Animal care and handling were performed according to an approved protocol of the University of Illinois at Chicago Animal Care Committee. The transgenic mice are described in Supplemental Experimental Procedures. For measurements of *k*_*f,c*_, isolated lungs were perfused with RPMI medium at constant flow (2 ml/min), temperature (37°C), and venous pressure (4 cm H_2_O) as previously described (Garcia et al., 2011). The preparation was ventilated at 120 breaths/min with constant peak inspiratory (∼10 cm H_2_O) and end expiratory pressures (2 cmH_2_O). Lung weight change was recorded with a force-displacement transducer (Model FT03C, Grass Technologies). A 20-min equilibration perfusion established isogravimetric conditions, before measuring the gravimetric filtration coefficient (*k*_*f,c*_) by comparing the rate of lung weight-gain during a baseline isogravimetric period with the rate after a change in hydrostatic pressure of at least 6 cm H_2_O for 20 min. The filtration rate was determined from the slope of the weight-gain curve between 15 and 20 min after PAR1-AP infusion. *k*_*f,c*_ was calculated from:

$$k_{fc}=\;\frac{\Delta W/\Delta t}{\Delta P},$$

where W, weight; t, time; P, pulmonary microvascular pressure.

Endothelial lysates were collected after each measurement of *k*_*f,c*_ via a left atrial cannula perfused with buffer (50 mM Tris-Cl (pH 7.8), 0.2% Triton X-100, and protease and phosphatase inhibitor cocktails; 0.4 ml/min). Fractions were collected at 1-min intervals. Fractions 2 and 3, which were positive for VE-cadherin (endothelial marker) and negative for smooth muscle actin, were used for assessment of EB3 expression by western blot.

### Statistical Analyses

Comparisons between groups were made using ANOVA with the Tukey post-test method or Student’s t test. Significance values are shown by ^∗^p < 0.05, ^∗∗^p < 0.01, and ^∗∗∗^p < 0.001.

## Author Contributions

Y.A.K. conceived the project; M.G., F.H., Y.S., S.M.V., and Y.A.K. conducted experiments; M.G., C.W.T., and Y.A.K. analyzed and interpreted data; M.G., C.W.T., A.B.M., and Y.A.K. wrote the paper.

## Figures and Tables

**Table 1:** Effects of α-Thrombin and Loss of EB3 on Microtubule Dynamics

	Growth Rate (μm/min)	Catastrophe Frequency (min^–1^)
**Basal**		

Control siRNA	13.7 ± 3.1	6.2 ± 4.2
EB3 siRNA	14.1 ± 2.9	5.5 ± 2.2

**α-thrombin**		

Control siRNA	11.0 ± 3.0^a^	7.4 ± 4.6
EB3 siRNA	12.1 ± 3.0^a^	6.6 ± 6.5

Growth rates of microtubules were calculated from the histogram of instantaneous displacement rates of microtubule tips between frames collected every 3 s (Figure S2). Catastrophe frequency was calculated from the number of shortening events per min (Supplemental Experimental Procedures). Results are means ± SD (n = 7–8 cells).

a Paired two-tailed Student’s t test compared to unstimulated cells.

## Supplemental Information

Document S1. Supplemental Experimental Procedures, Figures S1–S5, and Tables S1–S7

Document S2. Article plus Supplemental Information
