# Supplementary material for: Microtubule-Associated Protein EB3 Regulates IP3 Receptor Clustering and Ca2+ Signaling in Endothelial Cells
Source: Cell Rep. 2015 Jun 25;12(1):79–89. doi: 10.1016/j.celrep.2015.06.001 (PMC4487770; doi:10.1016/j.celrep.2015.06.001)
Supplement: Document S1. Supplemental Experimental Procedures, Figures S1–S5, and Tables S1–S7 [file mmc1.pdf]

Cell Reports

Supplemental Information

Microtubule-Associated Protein EB3 Regulates IP3 Receptor Clustering and Ca2+ Signaling in Endothelial Cells

Melissa Geyer, Fei Huang, Ying Sun, Stephen M. Vogel, Asrar B. Malik, Colin W. Taylor, and Yulia A. Komarova

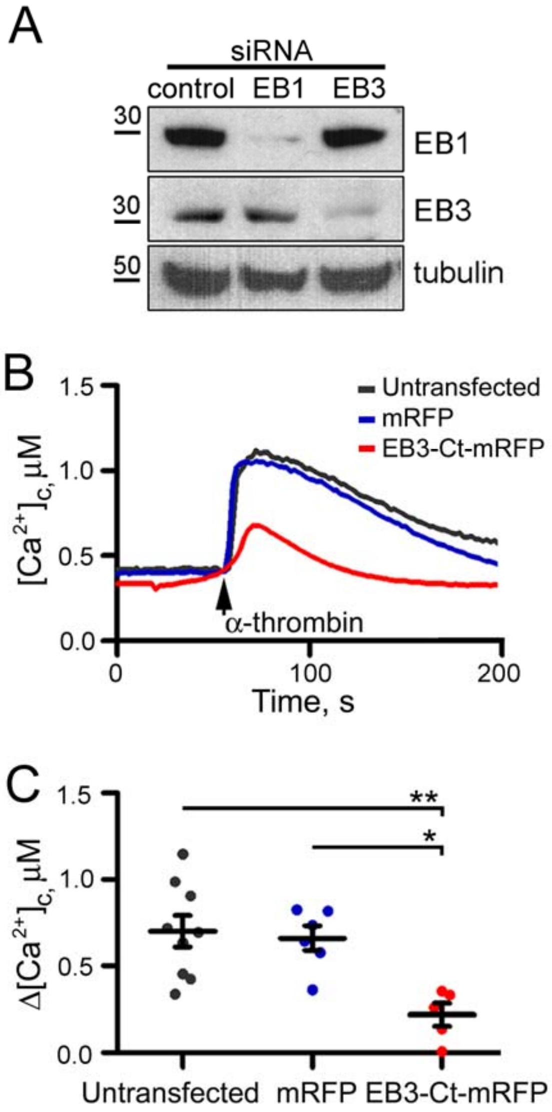

Figure S1. Attenuation of α-Thrombin-Evoked Ca^{2+} Signals by Loss of EB3. Related to Figure 1.

(A) Western blot using antibodies to EB1, EB3 and tubulin shows effects of the indicated siRNA treatments. M_r markers (kDa) are shown.

(B) Cytosolic Ca^{2+} signals evoked by addition of α-thrombin (50 nM) in Ca^{2+}-free medium recorded from HLMVECs expressing mRFP, a C-terminal fragment of EB3 tagged with mRFP (which has a dominant-negative effect by dimerizing with endogenous EBs and displacing them from microtubule plus ends) or from neighboring untransfected cells.

(C) Summary results show peak changes in $[Ca^{2+}]_c$ evoked by α-thrombin ($\Delta[Ca^{2+}]_c$). Individual data points are shown with mean ± SEM (n = 5-9 experiments). *, **, compared to untransfected cells using one-way ANOVA.

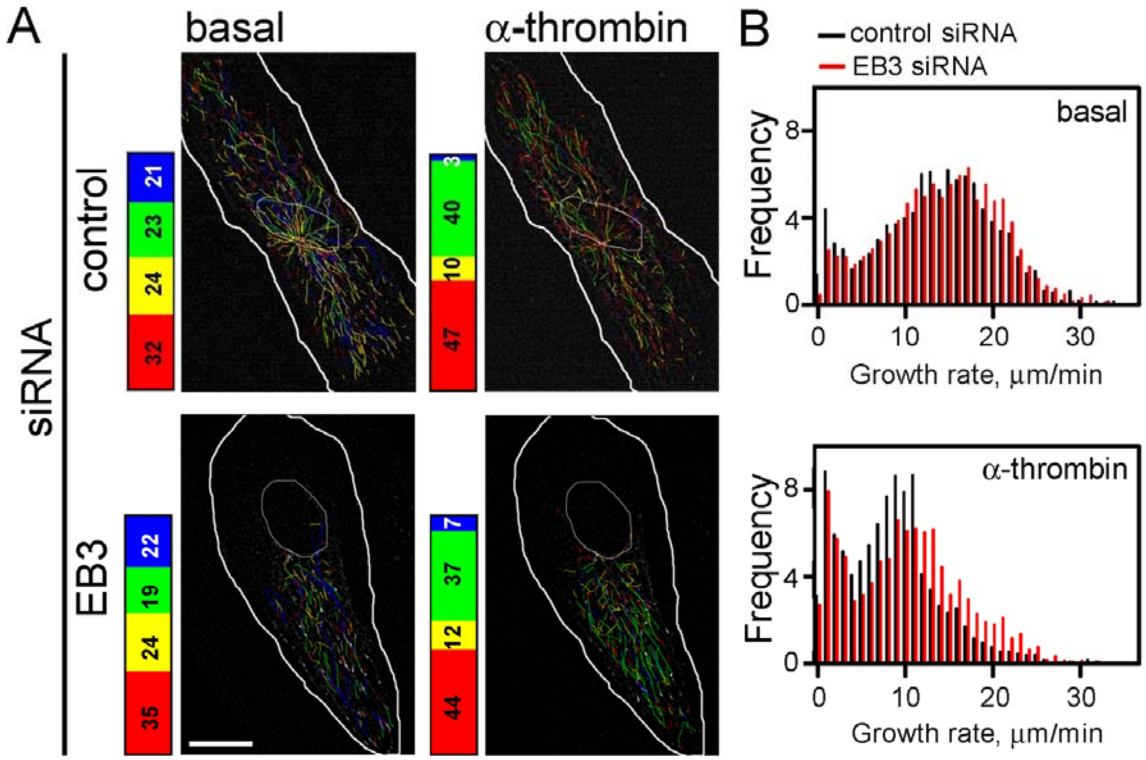

Figure S2. Loss of EB3 Does Not Affect Microtubule Dynamics in Unstimulated Cells or Within 5 min of Stimulation With α-Thrombin. Related to Table 1.

(A) Microtubule growth tracks classified by growth speed and lifetime in control and EB3 siRNA-treated cells before and then 5 min after addition of α-thrombin (50 nM). Blue, fast long-lived growth; green, slow long-lived growth; yellow, fast short-lived growth; red, slow short-lived growth (see Supplemental Experimental Procedures). Typical distributions of classified tracks and the percentage of tracks within each of the four categories (bars alongside each cell) are shown. Scale bar, 10 μm.

(B) Summary results (1907-2534 tracks, with 7-8 cells/group) show instantaneous microtubule growth rates for cells treated with control or EB3 siRNA before and within 5 min of stimulation with α-thrombin (50 nM). The percentage of tracks displaying each growth rate is shown.

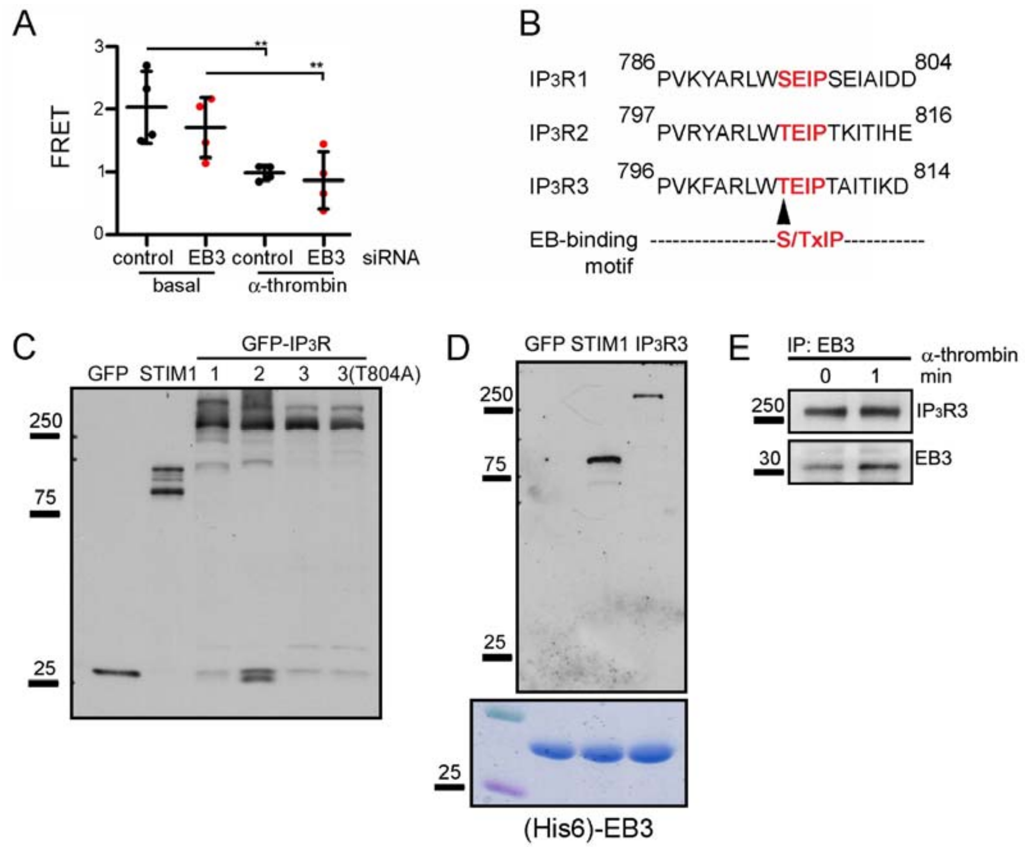

Figure S3. Association of IP_3 Receptors With EB3. Related to Figure 2.
(A) Cytosolic concentrations of IP_3 were measured in HLMVEC monolayers stimulated with α-thrombin (50 nM) using the FRET-based sensor LIBRAvIII (see Supplemental Experimental Procedures), where a decrease in FRET denotes an increase in IP_3 concentration. Individual data points and mean ± SEM (n = 4 cells) show the fluorescence emission ratio (FRET) (see Supplemental Experimental Procedures) measured 30 s after addition of α-thrombin to cells treated with control or EB3 siRNA. The results demonstrate that basal and α-thrombin-stimulated IP_3 levels were unaffected by loss of EB3. **, compared to basal level in the matched siRNA group, using one-way ANOVA.
(B) Sequence alignment of human IP_3Rs and EB-binding motif. Arrow designates T804 in IP_3R3.
(C) Western blot (using antibody to GFP) of GFP, STIM1-GFP and GFP-IP_3Rs in the lysates from HEK cells used for the pull-down experiments shown in Figures 2B and 2C.
(D) Pull-down of GFP, STIM1-GFP and GFP-IP_3R3 from lysates of HEK cells with (His6)-EB3, probed with an antibody to GFP. Lower panel shows Coomassie Brilliant Blue-stained gels loaded with 5% of the (His6)-EB3 used for the pull-down. Results (C and D) are typical of 2 experiments. M_r markers (kDa) are shown alongside each gel.
(E) An anti-EB3 antibody was used for immunoprecipitation (IP) of endogenous EB3 in lysates prepared from HPAECs before and 1 min after addition of α-thrombin. The western blot of the IP shows endogenous EB3 and IP_3R3. Results are typical of 2 experiments. M_r markers (kDa) are shown alongside the blot.

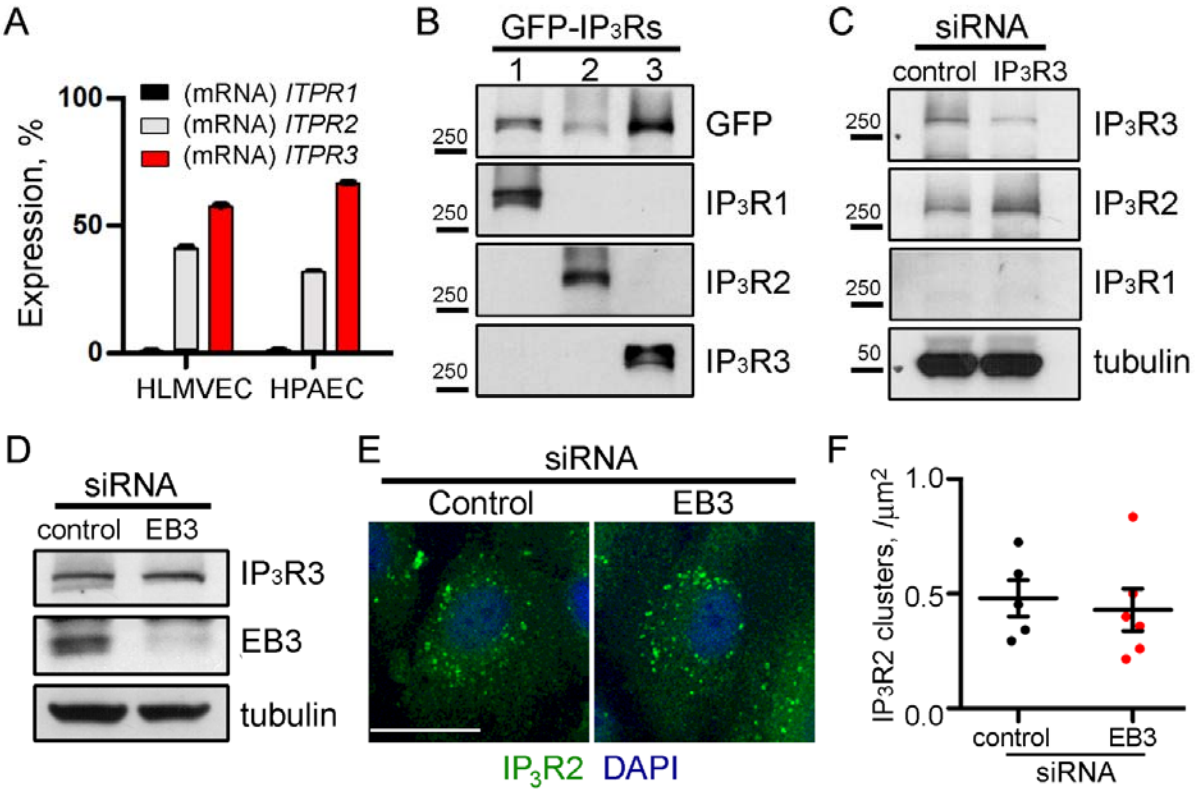

Figure S4. Loss of EB3 Does Not Affect Expression of IP₃R3 or Clustering of IP₃R2s. Related to Figure 3.

(A) Expression of IP3R subtypes in human pulmonary artery endothelial cells (HPAECs) and HLMVECs determined by QPCR. Results (means ± SD, n = 2) show the percentage of mRNA for each IP3R subtype.

(B) Characterization of antibodies to IP3R1 and IP3R2. Lysates of HEK cells overexpressing GFP-IP3R1, GFP-IP3R2 or GFP-IP3R3 were used for western blots with rabbit polyclonal antibodies for IP3R1 and IP3R2, and mouse monoclonal antibody for IP3R3 (see tables in Supplemental Experimental Procedures). The results (because the over-expressed GFP-IP3Rs are much more abundant than endogenous IP3Rs) confirm the subtype-specificity of the antibodies.

(C) Expression of IP3R subtypes in HLMVECs assessed by western blot analysis. Only IP3R2 and IP3R3 were detected in HLMVECs. Depletion of IP3R3 with siRNA did not cause upregulation of IP3R1. Results are typical of 2 independent experiments. M_r markers (kDa) are shown alongside the blots.

(D) Loss of EB3 does not affect IP3R3 expression. Western blot analysis of HPAECs treated with control or EB3 siRNA and probed for EB3 and IP3R3; tubulin is a loading control. Typical results of 4 independent experiments.

(E) Distribution of endogenous IP3R2 in HLMVECs treated with control and EB3 siRNA. Nuclei are stained with DAPI. Scale bar, 10 μm.

(F) Quantification of the numbers of IP3R2 clusters demonstrates that loss of EB3 does not affect IP3R2 clustering. Individual points with mean ± SEM are shown, n = 5-6 images per group.

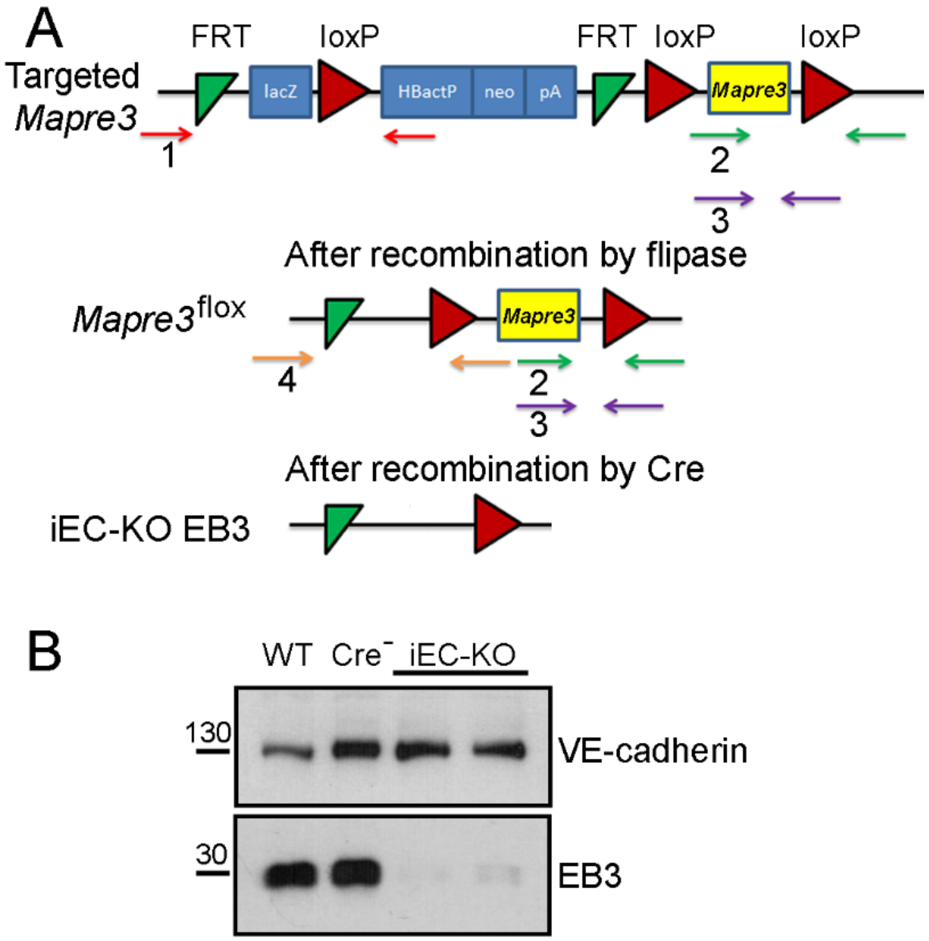

Figure S5. Generation of Mouse Model for Tamoxifen-Inducible Deletion of *mapre3* Gene in Endothelium. Related to Figure 6.

(A) Schematic representation of inserted transgenes and two-step protocol for generating inducible endothelium-specific (iEC*)* knockout (KO) of EB3. *Mapre3*-loxP-targeted transgenic mice were generated by flanking the *Mapre3* gene with loxP sites; a promoter-driving selection cassette (L1L2-Bact-P) was flanked by FRT sites and inserted upstream of the *Mapre3* critical exon. The L1L2-Bact-P cassette was removed by crossing mice with flipase-mediated recombination (Gt(ROSA)-26Sortm2(FLP*)Sor mice) generating *Mapre3*$^{flox/flox}$ mice. Ubiquitous deletion of EB3 in endothelium was achieved by crossing *Mapre3*$^{flox/flox}$ to *Tie2-Cre*ER^{T2} and upon stimulation of *Cre* recombinase with tamoxifen.

(B) Deletion of EB3 in endothelium was confirmed by probing lung endothelium-specific fractions (collected from lung after each $k_{f,c}$ trial, see Experimental Procedures) for VE-cadherin and EB3 on western blots. Wild-type (WT), C57/B6 control untreated mouse; *Cre*$^{-}$, *Mapre3*$^{flox/flox}$ mouse treated with tamoxifen; iECKO, *Mapre3*$^{flox/flox}$ -*Tie2-Cre*ER^{T2} mice treated with tamoxifen.

SUPPLEMENTAL EXPERIMENTAL PROCEDURES

Expression of EB Proteins and Pull-Down Assays

Preparation of (His6)-tagged EB1 and EB3-Ct was described previously (Komarova et al, 2009). (His6)-TEV-EB3 and (His6)-TEV-EB3ΔAc were generated by PCR and sub-cloned into the modified pRSF-Duet-1 vector (EMD Millipore) at BamH1 and HindIII sites. Details of these, and the other constructs used, are provided in the supplemental table S1 and S2, *Expression Constructs Used*.

(His6)-tagged proteins (Figure 2A) were expressed in *Escherichia coli* strain BL21 (DE3) (Stratagene). Bacteria were grown at 37ºC in LB medium containing 50 µg/ml kanamycin. When the OD_{600} reached 0.6-0.7, protein synthesis was induced by addition of isopropyl 1-thio-β-D-galactopyranoside (IPTG) to a final concentration of 250 µM. After 4 h at 30°C, bacterial pellets were isolated and sonicated (4 x 1 min) in medium comprising 150 mM NaCl, 5 mM 2-mercaptoethanol, 2 mM $CaCl_2$, 10 mM imidazole, 2 mM PMSF, 25 mM Tris, pH 7.4.

(His6)-EB proteins were purified using Ni-NTA beads (Thermo Scientific) and then covalently attached to them. Ni-NTA beads (1 ml) in a 20 ml column (Bio-Rad) were equilibrated with 50 bed-volumes of binding buffer (25 mM Tris, pH 7.4, 300 mM NaCl, 5 mM 2-mercaptoethanol, 2 mM PMSF). Bacterial lysate (50 ml) was then added to the column, followed by washing (150 bed-volumes of wash buffer, ~75 ml). The protein-bound beads were washed with phosphate-buffered saline (PBS) supplemented with 2 mM $CaCl_2$ and protease inhibitor cocktail (Sigma) and stored in the same medium. A sample of the beads was heated in sample buffer at 95°C for 5 min and SDS-PAGE was used to determine the purity of the (His6)-EB proteins (typically 90%). The concentration of EB protein was determined from densitometric analysis of Coomassie Brilliant Blue staining with BSA as a standard. For cross-linking of (His6)-EB to the Ni-NTA beads, 40-50 µl of (His6)-tagged proteins bound to beads were incubated with N-hydroxysuccinimide (75 mM) and 1-ethyl-3-carbodiimide (50 mM) in 20 mM Hepes buffer, pH ~7.0 (1 ml for 1 h at 4°C). The beads were washed with PBS (2 x 1 ml) and incubated with PBS containing 5% BSA for 1 h to block non-specific binding before use for pull-downs.

For pull-downs analyses, HEK 293 cells expressing GFP-tagged proteins were lysed in medium comprising 150 mM NaCl, 50 mM Tris, pH 8.0, 1% NP40 (Sigma), and 1% protease inhibitor cocktail (Sigma). The lysates (~300 µl/sample) containing equal amounts of GFP-tagged proteins (Figure S3) were incubated with the (His6)-EB/Ni-NTA beads (50 µl) in the presence of 10 mM imidazole at 4°C for 2 h. Bound proteins were eluted by heating in sample buffer at 95°C for 5 min before SDS-PAGE using 4-20% tris-glycine gels (Life Technologies). After transfer to a nitrocellulose membrane (Bio-Rad), the blots were probed with anti-GFP antibodies. Details of the antibodies used for these, and all other experiments with antibodies, are provided in the supplemental tables S3 and S4, *Primary Antibodies Used*, and *Secondary Antibodies Used*.

QPCR Analysis of mRNA Expression

Total RNA was isolated using RNeasy mini Kit (Qiagen), quantified (absorbance at 260 nm) and reverse transcribed using iScript according to the manufacturer's instructions (BioRad). The cDNA was amplified by real-time PCR using SYBR® Green Master Mix gene expression assay (Integrated DNA Technologies) and the primers listed in the supplemental table S6, *Primers Used*.

For each QPCR, amplification efficiency (E) was calculated as 10^m, where m is the mean increase in fluorescence after the cycle threshold (C_T) as previously described (Govindan et al., 2010). Expression levels of *ITPR*s were calculated relative to glucose-6-phosphate dehydrogenase (G6PD), a housekeeping product:

$$\frac{E^{C_T(ITPR)}}{E^{C_T(G6PD)}}$$

Each QPCR reaction was performed in duplicate with samples from three different wells.

FRET Analysis of EB3 Interactions with IP_3Rs

FRET between GFP-IP_3R3 and EB3-mRFP or mRFP-CLIP-170 in CHO-K1 cells was assessed by measuring the recovery of donor (GFP) fluorescence after acceptor (mRFP) photo-bleaching. A selected region (~0.5 µm across) across the anticipated path of microtubule growth (predicted from the linear trajectory of the growing microtubule) was repeatedly photo-bleached (543 nm, 7 cycles of 500 ms at 100% laser power) resulting in 70-80% bleaching of the acceptor. The intensity of the GFP fluorescence before (I_D) and immediately after (I_{DA}) acceptor bleaching was used to calculate the fluorescence recovery: $(I_{DA}-I_D)/I_{DA}$ (Yamamura et al., 2012).

Live-Cell Imaging of GFP-IP_3R3 and Analyses of IP_3R Clustering

Time-lapse images of GFP-IP_3R3 and GFP-IP_3R3(T804A) in HLMVECs were acquired at 37ºC using a Nikon Eclipse TE-2000S microscope with an UltraView confocal head (PerkinElmer Life Sciences), ORCA-ER-1394 camera (Hamamatsu), Ar-ion 488-nm laser, a Plan Apo 100x 1.4 NA objective, and Volocity 5 software (Improvision). Images were acquired every 0.5 s before and for 5 min after stimulation with α-thrombin. To calculate the number of α-thrombin-evoked GFP-IP_3R3 clusters, uneven backgrounds were corrected in all images using the flatten background command (Metamorph). Fluorescence from basal IP_3R3 clusters was subtracted from each post-stimulation image using a projected image from 10 frames collected before stimulation with α-thrombin. Photo-bleaching during image acquisition was disregarded because it was negligible: the bleaching constant of GFP-IP_3R3 fluorescence, determined from exponential curve-fitting, was $0.0005 \pm 0.0003\ s^{-1}$. All images of GFP- IP_3R3 (or immunostained endogenous IP_3Rs) were thresholded and the number of clusters was counted using the Intregated Morphometry Analysis tool (Metamorph).

Quantification of Microtubule Dynamics

Microtubule dynamics were determined from analysis of EB1-GFP tracks in HLMVECs treated with control or EB3 siRNA before and after challenge with α-thrombin. Images were acquired at 37°C at 3-s intervals using the 488-nm Ar-ion laser line of the Nikon confocal microscope described above. Detection, tracking and analysis were performed on the 50-60 frames before and after challenge with α-thrombin using PlustTipTracker software as described previously (Komarova et al., 2012). Microtubule growth rates were determined for microtubules at least 3 µm from the cell cortex. Rates were calculated from the histogram of instantaneous growth rates, that

is the displacement of GFP-EB1-positive microtubule tips between sequential frames. The catastrophe frequency was calculated from the number of shortening (catastrophe) events per min. Because backward gaps can represent pause or shrinkage, the PlustTipTracker software algorithm distinguished between these events based on the gap speed distributions (Applegate et al., 2011). If a microtubule plus end displacement yields a speed slower than the 95th percentile of the speed, the backward gap was assumed to be a pause with 95% confidence. The remaining backward gaps were classified as shrinkage events. The 'Quadrant Scatter Plot' tool, a component of the PlusTipTracker package, was used to determine the relationship between growth rate and growth lifetime (persistence) and to divide microtubules into four sub-populations based on deviation from mean growth speed and growth lifetime (Applegate et al., 2011) (see Figure S2A).

Measurement of Intracellular IP_3 Concentration

The cytosolic IP_3 concentration was measured in single cells with the FRET-based sensor LIBRAvIII (Tanimura et al., 2009). HLMVECs expressing LIBRAvIII were fixed in 4% paraformaldehyde before or after stimulation with α-thrombin (50 nM for 30 s). Using the Zeiss confocal microscope described above, ECFP (donor) fluorescence was excited (458 nm) while recording emission from ECFP with a band-pass BP500/20 nm filter and from the FRET acceptor EYFP with a long-pass LP530 nm filter. An EYFP image was also acquired (excitation at 514-nm; emission collected with the long-pass LP530 nm filter) and used to create an EYFP binary mask (i.e. an image in which each pixel has a value of 0 or 1). By multiplying the FRET signal in each pixel by this binary mask, the FRET analysis included only pixels with detectable fluorescence from the sensor. This modified FRET image was used to compute the relative IP_3 concentration in the cytoplasm for each cell from the ratio of the emission intensities for the acceptor/donor fluorophores when the donor was excited at 458 nm.

Dual-Channel Live-Cell Imaging of Fluorescent Proteins

Dual-channel simultaneous images of GFP-IP_3R3 and R-CEPIA1er, or EGFP-IP_3R3 and EB3-mRFP were acquired using the Zeiss confocal microscope described above. Cells were simultaneously excited with 488-nm and 561-nm lasers, and emitted light was collected by two gallium arsenide phosphide photomultiplier tubes after passing through band-pass filters (500-550 nm and 575-610 nm, respectively).

Generation of *Mapre3*$^{flox/flox}$- *Tie2-Cre*ER^{T2} Mice

All mice were maintained on a C57BL/6 genetic background. *Mapre3*-loxP-targeted transgenic mice, *Mapre3*$^{tm1a(EUCOMM)Wtsi}$, were purchased from the European Mutant Mouse Archive (EMMA). These mice were generated by inserting the promoter-driving selection L1L2-Bact-P cassette composed of an FRT site followed by lacZ sequence and a loxP site at position 30862104 of chromosome 5 upstream of the *Mapre3* critical exon. This first loxP site was followed by neomycin under the control of the human β-actin promoter, SV40 polyA, a second FRT site and a second loxP site. A third loxP site was inserted downstream of the targeted exon(s) at position 30862837 (Figure S5A).

To generate *Mapre3*$^{flox/flox}$, the Neo cassette was removed in all tissue types with flipase-mediated recombination. This was achieved by crossing *Mapre3*$^{tm1a(EUCOMM)Wtsi}$ with (Gt(ROSA)-26Sortm2(FLP*)Sor mice (Jackson Laboratory), in which widespread expression of the mouse codon-optimized FLP recombinase (FLPo) variant was driven by the *GT(ROSA)26Sor* promoter in a constitutive fashion. Subsequent crossing of *Mapre3*$^{flox/flox}$ to the *Tie2-Cre*ER^{T2} (Korhonen et al., 2009), in which high-fidelity endothelial expression (Forde et al., 2002) of the bacteriophage Cre/LoxP system was driven by regulatory elements from the *Tie2* promoter (Schlaeger et al., 1997) fused to a mutated estrogen receptor ligand-binding domain ERT2 (Feil et al., 1997). We confirmed that treatment with tamoxifen resulted in deletion of *Mapre3* gene in the pulmonary vascular endothelium (Figure S5B) although it is anticipated that Cre-mediated recombination and hence deletion of *Mapre3* gene should occur in the majority of endothelial cell populations (Forde et al., 2002). Genotyping was performed according to a protocol provided by EMMA using primers listed in the supplemental table S7, *DNA Primers Used (for Genotyping)*. No sex ratio disturbances were noted in the construction of strains or experimental progeny.

Four weeks old *Mapre3*$^{flox/flox}$ (used as a control for the experiments herein) and *Mapre3*$^{flox/flox}$-*Tie2-Cre*ER^{T2} mice were treated for 5 days with 75 mg/kg tamoxifen (Sigma-Aldrich) in corn oil administrated intraperitoneally. Mice were used for experiments on day 14 or later after *Cre*-induction.

Fractionation of Pulmonary Vascular Endothelium

Deletion of the EB3 gene in pulmonary endothelium was confirmed by western blot analyses of endothelium-specific fractions (Figure S5B). Fractions were collected after each $k_{f,c}$ trial from *ex vivo* lung preparations. The endothelial lysates were collected via a left atrial cannula by perfusing fractionation buffer (50 mM Tris-Cl, pH 7.8, 0.2% Triton X-100 and protease and phosphatase inhibitor cocktails). Fractions were collected every minute. Fractions 2 and 3, which were positive for VE-cadherin (endothelial marker) and negative for smooth muscle actin (marker of pericytes and smooth muscle cells), were used for assessment of EB3 expression.

Table S1. Expression Constructs Used (Mammalian) in This Study, Related to Figures 1-5.

Name	Residues	Description	Reference
EB3-GFP	FL, 1-281	EB3 with C-terminal EGFP tag	Stepanova et al., 2003
EB3-mRFP	FL, 1-281	mRFP-tagged EB3 in mRFP-N1 plasmid (R. Tsien, UCSD)	Komarova et al., 2009
GFP-EB3ΔAc	1-258	N-terminal EGFP linked (AQAGGSGGAGSGGEGAVDG) to EB3 lacking C-terminal	Komarova et al., 2009
EB3-Ct-mRFP	200-218	C-terminal residues of EB3 tagged with mRFP in mRFP-N1 plasmid (R. Tsien, UCSD)	
EB1-GFP	FL, 1-268	EB1 with C-terminal EGFP tag	Stepanova et al., 2003
mRFP-CLIP-170	FL, 1-1320	CLIP-170 N-terminally tagged with mRFP	Komarova et al., 2005
G-CEPIA1er		Green non-ratiometric Ca^{2+} sensor targeted to ER lumen.	Suzuki et al., 2014
R-CEPIA1er		Red non-ratiometric Ca^{2+} sensor targeted to ER lumen.	Suzuki et al., 2014
GEM-CEPIA1er		Ratiometric Ca^{2+} sensor targeted to ER lumen.	Suzuki et al., 2014
mCherry-er		mCherry targeted to ER lumen	Suzuki et al., 2014
LIBRAvIII		FRET-based IP_3 sensor	Tanimura et al., 2009
GFP-IP_3R1	FL	IP_3R1 with N-terminal EGFP tag	Pantazaka et al., 2011
GFP-IP_3R2	FL	IP_3R2 with N-terminal EGFP tag	Pantazaka et al., 2011
GFP-IP_3R3	FL	IP_3R3 with N-terminal EGFP tag	Wu et al., 2014
GFP-IP_3R3(T804A)	FL	EGFP-tagged IP_3R3 with mutation (T804A) within proposed EB-binding domain	
YFP-STIM1	FL	STIM1 with C-terminal EYFP tag	Zeng et al., 2008

Preparation of the EB3-Ct-mRFP and GFP-IP_3R3(T804A) constructs is described in Experimental Procedures. FL, full-length.

Table S2. Expression Constructs Used (Bacterial) in This Study, Related to Figure 2.

Name	Residues	Description	Reference
(His6)-EB1	FL, 1-281	Full length EB1, N-terminally tagged with His6	Komarova et al., 2009
(His6)-EB3	FL, 1-268	Full length EB3, N-terminally tagged with His6 tag and TEV cleavage site	Komarova et al., 2009
(His6)-EB3-Ct	200-281	C-terminal of EB3 with an N-terminal His6 tag	
(His6)-EB3-ΔAc	1-258	EB3 lacking C-terminal 23 residues, and with an N-terminal His6 tag and TEV cleavage site	

Preparation of the (His6)-EB3-Ct and (His6)-EB3-ΔAc constructs is described in Experimental Procedures.

Table S3. Primary Antibodies Used in This Study, Related to Figures 2, 3 and 6.

**Antibody**	**Species**	**Source**	**Application**
EB1	Rat	Absea (010811B11)	WB (1:3,000)<br>Figure S1A
EB3	Rat	Absea (010320D02)	WB (1:3,000)<br>Figures S1A, S3E, S4D, S5B
GFP	Mouse	Life Technologies (A11120, A11121)	WB (1:1,000)<br>Figures S3C, S3D
GFP	Rabbit	Life Technologies (A11122)	WB (1:1000)<br>Figures 2B, 2C, S4B
α-tubulin	Mouse	Sigma (T5168)	WB (1:1,000)<br>Figures 6A, 6B, S1A, S4C, S4D
IP_3R1	Rabbit	In house against CLLGHPPHMNVNPQQPA	WB (1:1000)<br>Figures S4B, S4C
IP_3R2	Rabbit	In house against CPDYRDAQNEGKTVRDGELP	WB (1:1,000)<br>Figures S4B, S4C
IP_3R2	Rabbit	Millipore (AB3000)	IC (1:100)<br>Figures S4E, S4F
IP_3R3	Rabbit	AbCam (ab78556)	IC (1:100)<br>Figures 3A, 3B
IP_3R3	Mouse	BD Biosciences (610312)	WB (1:1,000)<br>Figures S3E, S4B, S4C, S4F
VE-cadherin	Goat	Santa Cruz (sc-6458)	WB (1:1,000)<br>Figure S5B
p-MLC-II (Thr18/Ser19)	Rabbit	Cell Signaling (3674P)	WB (1:1,000)<br>Figures 6A, 6B

WB, western blot; IC, immunocytochemistry. The dilutions used for each application are shown.

Table S4. Secondary Antibodies Used in This Study, Related to Figures 2, 3 and 6.

Antibody	Species	Catalogue #	Application
HRP-conjugated anti-mouse	Donkey	715-035-150	WB (1:10,000)<br>Figures 6A, 6B, S1A, S3C, S3D<br>S3E, S4B, S4C, S4D
HRP-conjugated anti-rat	Mouse	212-055-082	WB (1:10,000)<br>Figures S1A, S4D, S5B
HRP-conjugated anti-rabbit	Donkey	711-035-152	WB (1:10,000)<br>Figures 2B, 2C, 6A, 6B, S4B, S4C
HRP-conjugated anti-goat	Donkey	705-055-003	WB (1:10,000)<br>Figure S5B
HRP-conjugated anti-rat IgG, Fcγ fragment-specific	Goat	112-035-071	WB (1:10,000)<br>Figure S3E
Cy5-conjugated anti-rabbit	Donkey	711-495-152	IC (1:200)<br>Figures 3A, S4E, S4F

WB, western blot; IC, immunocytochemistry. HRP, horseradish peroxidase; FITC, fluorescein isothiocyanate; Cy5, Cyanine 5 dye
All secondary antibodies were from Jackson ImmunoResearch Laboratories.

Table S5. The siRNAs Used in This Study, Related to Figures 1, 3 and 6.

Name	Target	Sense sequence	Cat. #
MAPRE1 siRNA	Human EB1	AAACGACCCUGUAUUGCAG	4392420
MAPRE3 siRNA	Human EB3	GAGCAUGAAUACAUCCACAUU	4392420
ITPR3 siRNA	Human IP_3R3	GCAUGGAGCAGAUCGUGUU	4392420
Silencer Select negative control #1			4390843

All siRNAs were from Life Technologies.

Table S6. DNA Primers Used (for QPCR) in This Study, Related to Supplemental Data, Figure S4.

Target	Forward	Reverse
G6PD	TGCCCCCGACCGTCTAC	ATGCGGTTCCAGCCTATCTG
ITPR1 (IP_3R1)	CTGATTCACCCACGAAGGTT	TGCAAATCAGGTGCTTTCTG
ITPR2 (IP_3R2)	GCTCTTGTCCCTGACATTG	CCCATGTCTCCATTCTCATAGC
ITPR3 (IP_3R3)	AGTGAGAAGCAGAAGAAGG	CATCCGGGGGAACCAGTC

All primers were from IDT DNA Technologies. *G6PD*, glucose-6-phosphate dehydrogenase.

Table S7. DNA Primers Used (for Genotyping) in This Study, Related to Figure 6.

Reaction	Forward	Reverse
1	AGGCTGGACCAGGGATTGGCCC	CCAACAGCTTCCCCACAACGG
2	CCACCAGCCAGGGTGACAAGGC	CCCCACCAGGGACTCAGGGACA
3	AGGAACCTAGCCCACCAGCCGC	ACTGATGGCGAGCTCAGACCATCCA
4	AGGAACCTAGCCCACCAGCCGC	GGGCTACAGCAGAGAACAGGGAG

The table provides the list of forward and reverse primers used to validate the genotype of *Mapre3*-loxP-targeted transgenic mice. All primers were from IDT DNA Technologies.

SUPPLEMENTAL REFERENCES

Applegate, K.T., Besson, S., Matov, A., Bagonis, M.H., Jaqaman, K., and Danuser, G. (2011). plusTipTracker: Quantitative image analysis software for the measurement of microtubule dynamics. J. Struct. Biol. *176*, 168-184.

Feil, R., Wagner, J., Metzger, D., and Chambon, P. (1997). Regulation of Cre recombinase activity by mutated estrogen receptor ligand-binding domains. Biochem. Biophys. Res. Commun. *237*, 752-757.

Forde, A., Constien, R., Grone, H.J., Hammerling, G., and Arnold, B. (2002). Temporal Cre-mediated recombination exclusively in endothelial cells using Tie2 regulatory elements. Genesis *33*, 191-197.

Govindan, S., Taylor, E.J., and Taylor, C.W. (2010). Ca^{2+} signalling by P2Y receptors in cultured rat aortic smooth muscle cells. Br. J. Pharmacol. *160*, 1953-1962.

Korhonen, H., Fisslthaler, B., Moers, A., Wirth, A., Habermehl, D., Wieland, T., Schutz, G., Wettschureck, N., Fleming, I., and Offermanns, S. (2009). Anaphylactic shock depends on endothelial G_q/G_{11}. J. Exp. Med. *206*, 411-420.

Morita, T., Tanimura, A., Nezu, A., and Tojyo, Y. (2002). Visualization of inositol 1,4,5-trisphosphate receptor type III with green fluorescent protein in living cells. Cell Calcium *31*, 59-64.

Schlaeger, T.M., Bartunkova, S., Lawitts, J.A., Teichmann, G., Risau, W., Deutsch, U., and Sato, T.N. (1997). Uniform vascular-endothelial-cell-specific gene expression in both embryonic and adult transgenic mice. Proc. Natl. Acad. Sci. USA *94*, 3058-3063.

Stepanova, T., Slemmer, J., Hoogenraad, C.C., Lansbergen, G., Dortland, B., De Zeeuw, C.I., Grosveld, F., van Cappellen, G., Akhmanova, A., and Galjart, N. (2003). Visualization of microtubule growth in cultured neurons via the use of EB3-GFP (end-binding protein 3-green fluorescent protein). J. Neurosci. *23*, 2655-2664.

Yamamura, H., Ikeda, C., Suzuki, Y., Ohya, S., and Imaizumi, Y. (2012). Molecular assembly and dynamics of fluorescent protein-tagged single KCa1.1 channel in expression system and vascular smooth muscle cells. Am. J. Physiol. *302*, C1257-1268.

Zeng, W., Yuan, J.P., Kim, M.S., Choi, Y.J., Huang, G.N., Worley, P.F., and Muallem, S. (2008). STIM1 gates TRPC channels, but not Orai1, by electrostatic interaction. Mol. Cell *32*, 439-448.
