# Supplementary material for: Microtubule-Associated Protein EB3 Regulates IP3 Receptor Clustering and Ca2+ Signaling in Endothelial Cells
Source: Cell Rep. 2015 Jun 25;12(1):79–89. doi: 10.1016/j.celrep.2015.06.001 (PMC4487770; doi:10.1016/j.celrep.2015.06.001)
Supplement: Document S2. Article plus Supplemental Information [file mmc2.pdf]

Cell Reports

Article

Microtubule-Associated Protein EB3 Regulates IP_3 Receptor Clustering and Ca^{2+} Signaling in Endothelial Cells

Graphical Abstract

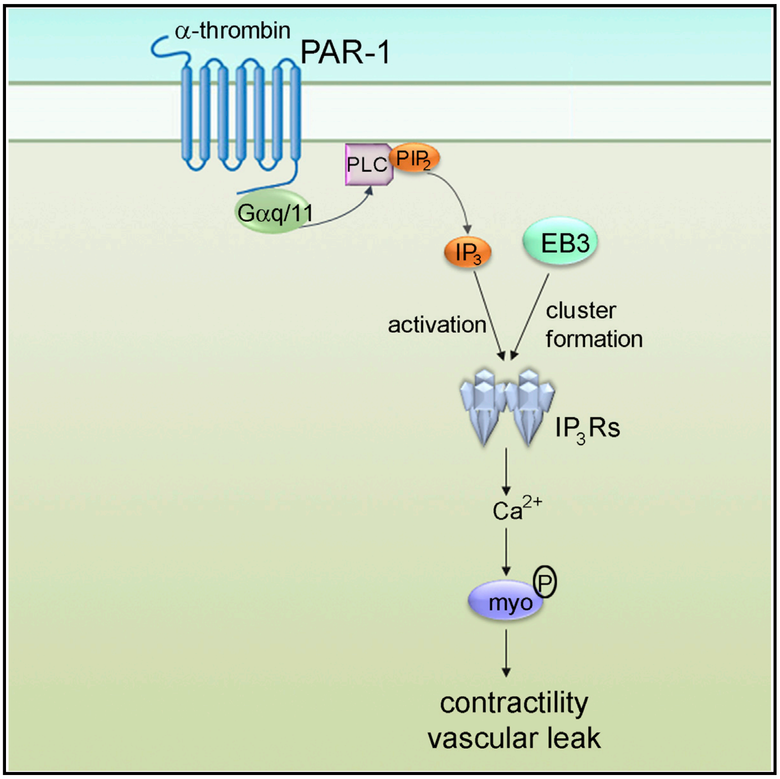

Highlights

- IP_3 receptors (IP_3Rs) bind to microtubule end-binding protein EB3
- EB3, in turn, promotes IP_3R clustering and Ca^{2+} signals in endothelial cells
- IP_3R-EB3 interaction thereby contributes to endothelial barrier disruption
- In vivo EB3 deletion in endothelial cells protects from vascular hyperpermeability

Authors

Melissa Geyer, Fei Huang, Ying Sun, ..., Asrar B. Malik, Colin W. Taylor, Yulia A. Komarova

Correspondence

ykomarov@uic.edu

In Brief

End binding proteins (EBs) mediate interactions between growing microtubules and intracellular structures. Geyer et al. demonstrate that interactions between EB3 and IP_3 receptors control clustering of IP_3 receptors in endothelial cells and Ca^{2+} signaling, and thus permeability of endothelial barrier in inflammatory diseases.

Geyer et al., 2015, Cell Reports *12*, 79–89
July 7, 2015 ©2015 The Authors
http://dx.doi.org/10.1016/j.celrep.2015.06.001

Cell Reports
Article

OPEN ACCESS
CellPress

Microtubule-Associated Protein EB3 Regulates IP_3 Receptor Clustering and Ca^{2+} Signaling in Endothelial Cells

Melissa Geyer,[1] Fei Huang,[1] Ying Sun,[1] Stephen M. Vogel,[1] Asrar B. Malik,[1] Colin W. Taylor,[2] and Yulia A. Komarova[1,*]
[1]Department of Pharmacology and The Center for Lung and Vascular Biology, University of Illinois College of Medicine, Chicago, IL 60612, USA
[2]Department of Pharmacology, University of Cambridge, Cambridge CB2 1PD, UK
*Correspondence: ykomarov@uic.edu
http://dx.doi.org/10.1016/j.celrep.2015.06.001
This is an open access article under the CC BY license (http://creativecommons.org/licenses/by/4.0/).

SUMMARY

The mechanisms by which the microtubule cytoskeleton regulates the permeability of endothelial barrier are not well understood. Here, we demonstrate that microtubule-associated end-binding protein 3 (EB3), a core component of the microtubule plus-end protein complex, binds to inositol 1,4,5-trisphosphate receptors (IP_3Rs) through an S/TxIP EB-binding motif. In endothelial cells, α-thrombin, a pro-inflammatory mediator that stimulates phospholipase Cβ, increases the cytosolic Ca^{2+} concentration and elicits clustering of IP_3R3s. These responses, and the resulting Ca^{2+}-dependent phosphorylation of myosin light chain, are prevented by depletion of either EB3 or mutation of the TxIP motif of IP_3R3 responsible for mediating its binding to EB3. We also show that selective EB3 gene deletion in endothelial cells of mice abrogates α-thrombin-induced increase in endothelial permeability. We conclude that the EB3-mediated interaction of IP_3Rs with microtubules controls the assembly of IP_3Rs into effective Ca^{2+} signaling clusters, which thereby regulate microtubule-dependent endothelial permeability.

INTRODUCTION

Adherens junctions (AJs) responsible for endothelial cell interactions (Dejana, 2004) and acto-myosin contraction (Wainwright et al., 2003) regulate the integrity of the vascular endothelial barrier. The ectodomain of vascular endothelial (VE)-cadherin, which is the primary adhesion molecule of AJs, undergoes homophilic *trans*-dimerization to form AJs, while its intracellular domain interacts with the actin cytoskeleton (Daneshjou et al., 2015; Giannotta et al., 2013). Activation of phospholipase C (PLC) via G protein-coupled receptors (GPCRs), such as the protease-activated receptor-1 (PAR-1), or by disruption of VE-cadherin *trans*-interactions (Komarova et al., 2012), causes increased endothelial permeability (Komarova and Malik, 2010). An increase in cytosolic Ca^{2+} concentration ($[Ca^{2+}]_c$), by promoting disassembly of AJs and acto-myosin-mediated contraction of endothelial cells, is a crucial signal mediating this increased endothelial permeability (Komarova and Malik, 2010).

The roles of actin polymerization and disassembly of AJs in increasing endothelial permeability have received considerable attention, but microtubules may also contribute to the response by poorly understood mechanisms (Vogel and Malik, 2012). Microtubules are polarized tubular filaments of heterodimers of α- and β-tubulin, with distinct plus and minus ends (Howard and Hyman, 2003). Plus ends, usually directed toward the cell periphery, undergo cycles of polymerization and depolymerization regulated by plus-end tracking proteins (+TIPs) (Akhmanova and Steinmetz, 2010). The end-binding proteins, EB1 and EB3, members of the RP/EB family, are core elements of the dynamic +TIP complex. EBs transiently bind to growing microtubules by recognizing the GTP-bound state of β-tubulin (Maurer et al., 2012). EB binding enhances lateral contacts between tubulin molecules and prevents the transition from microtubule growth to shrinkage ("catastrophe" events) (Komarova et al., 2009; Maurer et al., 2012). In addition, EBs provide an essential hub for assembly of other +TIPs that facilitate interactions of microtubules with various macromolecules and organelles (Akhmanova and Steinmetz, 2010). The latter include endoplasmic reticulum (ER), which is continuously remodeled through its interactions with microtubules (Pendin et al., 2011). These interactions involve both tethering of the ER protein, stromal interaction molecule 1 (STIM1), to the +TIP complex by EB1, and the association of ER tubules with the plus-end directed microtubule motor protein, kinesin 1 (Friedman and Voeltz, 2011; Grigoriev et al., 2008).

ER is the major intracellular Ca^{2+} store. Inositol 1,4,5-trisphosphate receptors (IP_3Rs) within ER membranes allow rapid release of Ca^{2+} from the ER (Foskett et al., 2007; Taylor et al., 2014). Emptying of ER Ca^{2+} stores then causes clustering of STIM1, and this activates store-operated Ca^{2+} entry into the cell across the plasma membrane (Wu et al., 2014). The release of Ca^{2+} from the ER evoked by IP_3 proceeds through recruitment of Ca^{2+} release events that depend on IP_3 priming IP_3Rs to respond to Ca^{2+}. This form of regulation allows clustered IP_3Rs to stimulate the activity of their neighbors by Ca^{2+}-induced

CrossMark

Ca^{2+} release (Smith et al., 2009). The lowest concentrations of IP_3 stimulate openings of single IP_3Rs, and as the IP_3 concentration increases the Ca^{2+} released by active IP_3Rs is thought to stimulate coordinated opening of IP_3Rs within a cluster, generating a Ca^{2+} "puff." Further increases in IP_3 concentration ignite regenerative Ca^{2+} waves that spread across the cell (Smith et al., 2009). The recruitment of IP_3R activity depends critically on the distribution of IP_3Rs in ER membranes, wherein most IP_3Rs appear to be mobile (Ferreri-Jacobia et al., 2005; Pantazaka and Taylor, 2011). Stimulation of PLC causes reversible clustering of IP_3Rs in cells (Chalmers et al., 2006; Tateishi et al., 2005) and within nuclear envelope IP_3 causes IP_3Rs to cluster (Taufiq-Ur-Rahman et al., 2009). Importantly in the context of the present study, IP_3Rs associate with microtubules (Takei et al., 1998) and the association contributes to the redistribution of IP_3Rs during sustained stimulation (Vermassen et al., 2003), extension of neuronal growth cones (Zhang and Forscher, 2009), and cell division (Mitsuyama and Sawai, 2001). There is, however, also evidence that IP_3R clustering can persist after disruption of microtubules (Wilson et al., 1998; Taufiq-Ur-Rahman et al., 2009).

Studies using drugs (colchicine, nocodazole, and taxol) suppressing microtubule dynamics suggest an important role of microtubule cytoskeleton in organizing IP_3-evoked Ca^{2+} signals (Isshiki et al., 1998; Fogarty et al., 2000). Perturbing the microtubule cytoskeleton inhibits receptor-activated release of Ca^{2+} via IP_3Rs (Tasaka et al., 1991), prevents initiation of Ca^{2+} waves (Béliveau and Guillemette, 2009; Isshiki et al., 1998), slows diffusion of IP_3Rs within ER membranes (Ferreri-Jacobia et al., 2005), disrupts local delivery of IP_3 to IP_3Rs (Graier et al., 1998; Ribeiro et al., 1997), and abolishes IP_3-activated Ca^{2+} spikes at the apical pole of secretory cells (Fogarty et al., 2000). Many of these perturbations might result from effects of microtubules in organizing the ER, but there is also the possibility of more direct interactions with IP_3Rs. The latter would be significant for vascular endothelial cells, where we have shown that PLC-evoked Ca^{2+} signals cause dephosphorylation of EB3 leading to persistent growth of microtubules, disassembly of AJs, and increased endothelial permeability (Komarova et al., 2012).

Here, we demonstrate a direct interaction between EB3 and the S/TxIP motif within IP_3R3 that allows IP_3Rs to associate with growing microtubule tips. Depletion of EB3 or disruption of the interaction with an IP_3R3 point mutation prevents both clustering of IP_3R3s and Ca^{2+} signals elicited by activation of PAR-1. Further, selective deletion of the EB3 gene in endothelium in mice inhibits the increase in endothelial permeability elicited by activation of PAR-1. Thus, microtubule-associated EB3 plays an obligatory role in organizing IP_3-induced Ca^{2+} signaling, and, in turn, regulating endothelial permeability.

RESULTS

Loss of EB3 Impairs Ca^{2+} Signaling in Endothelial Cells

In primary cultures of human lung microvascular endothelial cells (HLMVECs), α-thrombin stimulates PAR-1, a GPCR that causes activation of PLCβ, formation of IP_3 and release of Ca^{2+} from intracellular stores. Depletion of EB3, using small interfering RNA (siRNA) (Figure S1A), significantly reduced the amplitude of the Ca^{2+} signals evoked by addition of α-thrombin in Ca^{2+}-free medium and the subsequent response to restoration of extracellular Ca^{2+} (Figures 1A and 1B). The inhibition was substantially reversed by expression of a siRNA-resistant EB3-GFP, but not by EB1-GFP. Loss of EB1 had no significant effect on α-thrombin-activated Ca^{2+} signals. A C-terminal fragment of EB3 (EB3-Ct-mRFP), which prevents binding of endogenous EBs to microtubule tips by forming non-functional dimers with endogenous proteins (Komarova et al., 2009), also inhibited α-thrombin-induced Ca^{2+} signals (Figures S1B and S1C).

We used low-affinity, genetically encoded Ca^{2+} indicators expressed in the lumen of the ER, G-CEPIA1er and the ratiometric indicator GEM-CEPIA1er (Suzuki et al., 2014), to establish whether loss of EB3 affected the Ca^{2+} content of the intracellular stores. Depletion of EB3 affected neither the organization of the ER nor the free Ca^{2+} concentration within the ER ($[Ca^{2+}]_{ER}$) (Figures 1C–1E). $[Ca^{2+}]_{ER}$ was 583 ± 77 μM and 489 ± 53 μM (n = 6–16 cells) in control and EB3 siRNA-treated cells, respectively (Figure 1E). Stimulation of control cells with α-thrombin caused $[Ca^{2+}]_{ER}$ to fall to 96 ± 33 μM, whereas α-thrombin had no significant effect on cells treated with siRNA for EB3 (436 ± 122 μM) (Figure 1E). Furthermore, refilling of ER after restoration of extracellular Ca^{2+} to cells stimulated with α-thrombin in Ca^{2+}-free medium was faster in cells lacking EB3 (Figure 1D), consistent with reduced activation of IP_3Rs after knockdown of EB3. These results demonstrate that loss of EB3 attenuates the release of Ca^{2+} from intracellular stores elicited by α-thrombin without affecting the initial Ca^{2+} content of the ER.

EB3 Binds to IP_3Rs

To assess whether the effects of EB3 on Ca^{2+} release involved reorganization of the microtubule network, we analyzed changes in microtubule dynamics after addition of α-thrombin using time-lapse confocal imaging of EB1-GFP to mark growing microtubule tips. We chose EB1-GFP for this analysis because it does not rescue the inhibition of α-thrombin-evoked Ca^{2+} signals after EB3 depletion, and nor does loss of endogenous EB1 affect Ca^{2+} signals (Figures 1A and 1B). In confluent monolayers of HLMVECs treated with control siRNA, microtubules grew at 13.7 ± 3.1 μm/min, and they displayed frequent catastrophe events. Depletion of EB3 had no effect on the growth rate or catastrophe frequency (Table 1), suggesting that EB3 did not affect microtubule dynamics under basal conditions. This finding is consistent with previous work demonstrating that most EB3 is phosphorylated in unstimulated HLMVECs, and therefore unable to promote persistent growth of microtubules (Komarova et al., 2012). In the 2–3 min after stimulation of PAR-1 with α-thrombin, there was no effect on the catastrophe frequency, but the stimulation unexpectedly reduced microtubule growth rate in both control and EB3-siRNA-treated cells (Table 1; Figure S2). These results demonstrate that loss of EB3 has no discernible effect on microtubule dynamics under conditions where it attenuates α-thrombin-evoked Ca^{2+} release. We therefore considered whether EB3 might regulate PLC or IP_3Rs.

We used the IP_3 biosensor, LIBRAvIII, in which IP_3 binding causes a decrease in intramolecular fluorescence resonance energy transfer (FRET) (Tanimura et al., 2009), to measure cytosolic IP_3 concentrations in confluent monolayers of HLMVECs.

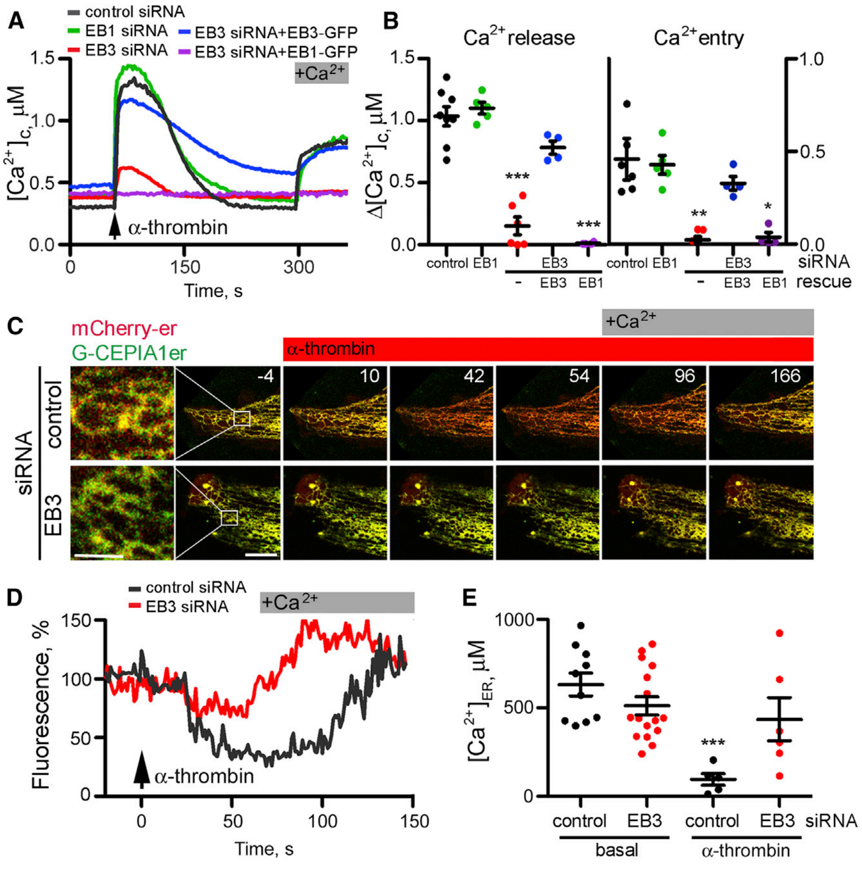

Figure 1. Depletion of EB3 Attenuates Receptor-Induced Ca^{2+} Release from ER

(A) Cytosolic Ca^{2+} signals evoked by addition of α-thrombin (50 nM) recorded from ~5–10 fura-2-loaded HLMVECs in Ca^{2+}-free medium, followed by restoration of extracellular Ca^{2+} (2 mM). Cells were treated with siRNA for EB1 or EB3, control siRNA, or siRNA for EB3 together with expression of EB1-GFP or a siRNA-resistant EB3-GFP.

(B) Summary results show peak changes in $[Ca^{2+}]_c$ ($\Delta[Ca^{2+}]_c$) evoked by α-thrombin (Ca^{2+} release) and the subsequent restoration of extracellular Ca^{2+} (Ca^{2+} entry). Results show data points color coded as in (A), and mean ± SEM from four to eight experiments with five to ten cells analyzed in each group. *,**,***p values are relative to control siRNA-treated cells using one-way ANOVA.

(C) Time-lapse images (times shown in seconds) show overlaid red (mCherry-ER) and green (G-CEPIA1er) fluorescence for cells treated with control or EB3 siRNA. Cells were stimulated with α-thrombin (50 nM at t = 0) in Ca^{2+}-free medium, before restoration of extracellular Ca^{2+}. Loss of luminal Ca^{2+} causes the green fluorescence of the luminal Ca^{2+} indicator to decrease, shifting images from yellow to red. The first panel shows an enlargement of the boxed area. Scale bars, 1 (enlargement) and 10 μm (other panels).

(D) Experiments similar to those in (C) show the time course of the changes in $[Ca^{2+}]_{ER}$ presented as fluorescence ratios (G-CEPIA/mCherry-ER) normalized to the ratio recorded 4 s before addition of α-thrombin (100%) for cells treated with control or EB3 siRNA.

(E) Summary results (mean ± SEM from 6 to 16 cells) show $[Ca^{2+}]_{ER}$ measured before (basal) and after stimulation with α-thrombin (50 nM) using GEM-CEPIA1er in cells treated with control or EB3 siRNA. Results are from two independent experiments; ***relative to matched siRNA treatment without α-thrombin stimulation, using one-way ANOVA.

α-Thrombin caused a decrease in the FRET signal consistent with an increase in cytosolic IP_3 concentration. The response was similar in cells treated with control siRNA or siRNA to EB3 (Figure S3A). These results thus suggest that under conditions where loss of EB3 attenuates α-thrombin-evoked Ca^{2+} signals, EB3 has no effect on α-thrombin-induced IP_3 formation.

Alignment of IP_3R sequences identified a conserved SxIP motif within a short region of disordered protein structure in all three mammalian IP_3R subtypes (Figure S3B). This motif (residues 804–807 in human IP_3R3), a signature of EB-interacting partners (Honnappa et al., 2009), is located downstream of the IP_3-binding site. The interaction between full-length EB3 or its C-terminal region (residues 200–281), and IP_3R3 was demonstrated in pull-down assays using immobilized (His6)-EBs expressed in bacteria and GFP-IP_3Rs from lysates of HEK cells (Figures 2A, 2B, and S3C). IP_3R1 and IP_3R2 also associated with full-length EB3 (Figure 2C). EB1 was less effective than EB3 in the pull-downs of GFP-IP_3R3 (Figure 2B). For each IP_3R subtype, the interaction with EB3 was weaker than that between EB3 and STIM1, another Ca^{2+} signaling protein to which EB1 and EB3 bind (Grigoriev et al., 2008) (Figure S3D). Interaction between endogenous EB3 and IP_3R3 was confirmed by their co-immunoprecipitation (Figure S3E). Deletion of the acidic C-terminal tail of EB3 (EB3ΔAc), which contributes to the binding interface for the S/TxIP motif, abolished the interaction of IP_3R3 with EB3 (Figure 2B). Mutation of the critical Thr within the TxIP motif of IP_3R3 (T804A) also abolished the interaction with EB3 (Figure 2C). These results suggest a direct interaction between the C-terminal region of EB3 and the TxIP motif of IP_3R3, an interaction that is probably shared with other IP_3R subtypes.

In HLMVECs expressing EB3-mRFP and GFP-IP_3R3, there were transient contacts between growing microtubule tips and GFP-IP_3R3 in ER membranes (Figure 2D). However, GFP-IP_3R3 did not form the "comet-like" structures described for STIM-1 associated with growing microtubule tips (Grigoriev et al., 2008; Pozo-Guisado et al., 2010). This finding is consistent with the lower affinity of EB3 relative to STIM1 for IP_3R3, because comets reveal the density of EB proteins, which declines with distance from the microtubule tip. We also observed quenching of the GFP fluorescence when EB3-mRFP-labeled microtubule tips made contact with ER tubules, suggesting an intermolecular FRET between EB3-mRFP and GFP-IP_3R3 (Figures 2D and 2E). Focal photobleaching of the EB3-mRFP acceptor caused a transient increase in closely apposed fluorescence of the GFP-IP_3R3 donor (Figure 2F), confirming an interaction between EB3 and IP_3R3 in intact cells. There was no detectable FRET between

Table 1. Effects of α-Thrombin and Loss of EB3 on Microtubule Dynamics

	Growth Rate (μm/min)	Catastrophe Frequency (min^{-1})
Basal		
Control siRNA	13.7 ± 3.1	6.2 ± 4.2
EB3 siRNA	14.1 ± 2.9	5.5 ± 2.2
α-thrombin		
Control siRNA	11.0 ± 3.0[a]	7.4 ± 4.6
EB3 siRNA	12.1 ± 3.0[a]	6.6 ± 6.5

Growth rates of microtubules were calculated from the histogram of instantaneous displacement rates of microtubule tips between frames collected every 3 s (Figure S2). Catastrophe frequency was calculated from the number of shortening events per min (Supplemental Experimental Procedures). Results are means ± SD (n = 7–8 cells).
[a]Paired two-tailed Student's t test compared to unstimulated cells.

GFP-IP_3R3 and another +TIP, mRFP-CLIP-170 (Figure 2F). The results thus indicate a specific interaction between EB3 and IP_3R3 in intact human lung endothelial cells.

Interactions between EB3 and IP_3R3 Regulate IP_3R3 Dynamic and Activity

Analysis of mRNA and protein expression demonstrated that in various human pulmonary endothelial cells, including HLMVECs, IP_3R2 and IP_3R3 were the major subtypes (Figures S4A–S4C). Immunostaining of HLMVECs revealed that IP_3R3 formed puncta (Figure 3A), consistent with their assembly into clusters, as reported for IP_3Rs in endothelial cells (Tran et al., 2014). Depletion of EB3 had no effect on the expression of IP_3R3 (Figure S4D), but it significantly reduced the number of IP_3R3 clusters (Figures 3A and 3B). EB3 depletion had no effect on IP_3R2 clusters, which were observed in the perinuclear region in both control and EB3 siRNA-treated cells (Figures S4E and S4F). The inhibition of IP_3R3 clustering by depletion of EB3 was reversed by expression of a siRNA-resistant EB3, but not by expression of the EB3ΔAc mutant (Figure 3B) that does not bind to IP_3R3 (Figure 2B).

GFP-IP_3R3 also formed clusters in unstimulated HLMVECs, with fewer clusters in cells lacking EB3 (Figure 3C). This allowed dynamic imaging of GFP-IP_3R3 distribution in response to receptor activation. Stimulation of HLMVECs expressing GFP-IP_3R3 with α-thrombin evoked a rapid transient increase in IP_3R3 clustering that peaked after ~30 s and persisted for 190 ± 15 s (time for 90% of clusters to disappear) (Figures 3C–3E). Similar clustering of IP_3Rs in response to stimuli that caused IP_3R activation has been reported in other cells (Taufiq-Ur-Rahman et al., 2009; Tateishi et al., 2005). Both the number of IP_3R3 clusters after stimulation with α-thrombin and their lifespan (64 ± 28 s) were reduced in EB3-depleted HLMVECs (Figures 3D and 3E).

Residue T804 within the TEIP motif of IP_3R3 was required for IP_3R3 binding to EB3 (Figure 2C). Expression of GFP-IP_3R3 (T804A) in HLMVECs attenuated α-thrombin-evoked Ca^{2+} signals. This was evident from the smaller effects of α-thrombin on both the decrease in $[Ca^{2+}]_{ER}$ and increase in $[Ca^{2+}]_c$ in cells

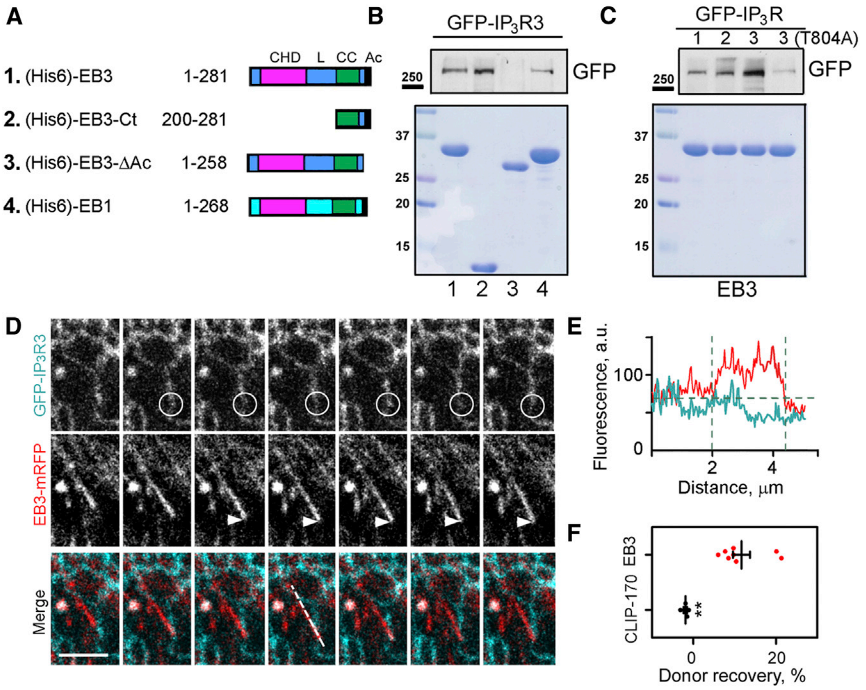

Figure 2. EB3 Interacts Directly with IP_3Rs

(A) Schematic representation of hexa-histidine (His6)-tagged EB constructs used for pull-down experiments. CHD, calponin homology domain; L, linker; CC, coiled coil; Ac, acidic tail.

(B and C) Pull-down analyses of interactions between (His6)-EB proteins covalently bound to Ni-NTA resin and lysates from HEK cells expressing GFP-IP_3R1-3 or GFP-IP_3R3(T804A). Upper panels show western blots for GFP and lower panels show Coomassie brilliant blue-stained gels loaded with 5% of the EBs (numbered as in A) used for the pull-down. Results are typical of three independent experiments. Western blots of GFP-IP_3Rs in the cell lysates used and additional controls are shown in Figure S3.

(D) Confocal images collected at 850-ms intervals show simultaneous recordings of fluorescence from EB3-mRFP and GFP-IP_3R3 in HLMVECs. The composite panels show overlaid GFP-IP_3R3 (green) and EB3-mRFP (red). Note the loss of GFP-IP_3R3 fluorescence (circle) as the EB3-mRFP-labeled microtubule tip approaches (arrow). Scale bar, 5 μm.

(E) EB3-mRFP and GFP-IP_3R3 fluorescence recorded along the dashed line shown in the merged images in (D) illustrates the decrease in GFP fluorescence at the point of interaction with the microtubule tip.

(F) Focal photobleaching of the acceptor fluorophore (mRFP) at the microtubule tip while recording donor fluorescence from GFP-IP_3R was used to assess the interaction between GFP-IP_3R3 and EB3-mRFP or CLIP-170-mRFP at the microtubule tip. Individual data points with mean ± SEM from five to eight cells analyzed in each group show the recovery of the donor fluorescence after acceptor photobleaching (%). **Using Student's t test.

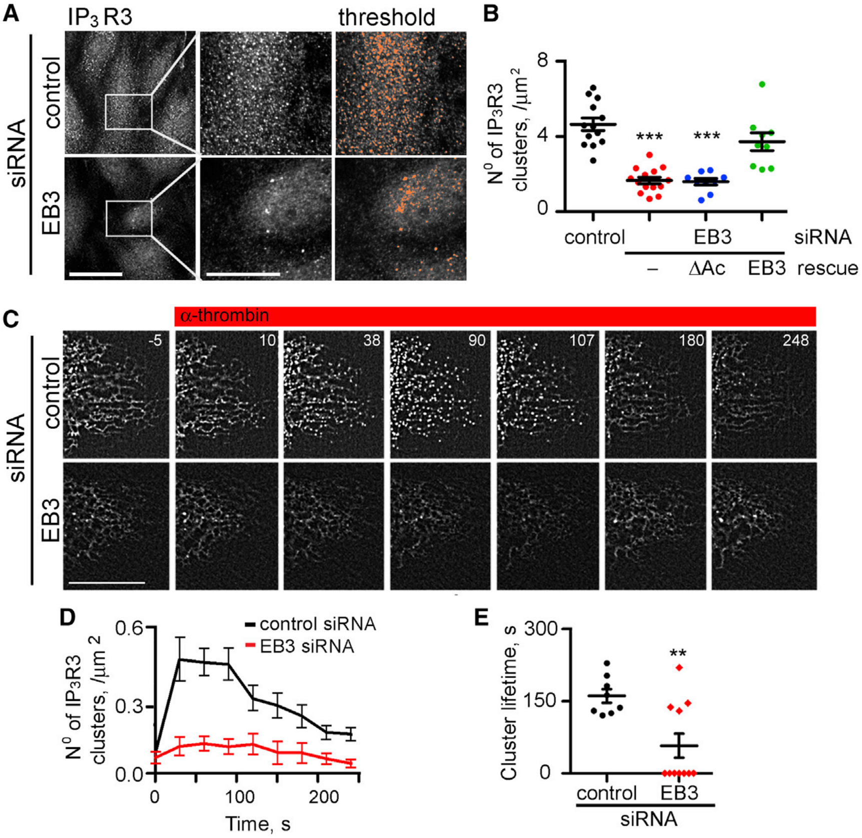

Figure 3. EB3 Facilitates Clustering of IP_3R3

(A) Intracellular distribution of endogenous IP_3R3 (immunostaining) in HLMVECs treated with control or EB3 siRNA. Central panels show enlargements of the boxed area. The thresholded images used to measure cluster densities (see Experimental Procedures) are shown in the right panels. Scale bar, 10 μm.

(B) Summary results from nine to 14 cells show numbers of IP_3R3 clusters in cells treated with control or EB3 siRNA alone or after rescue with EB3-GFP or EB3ΔAc-GFP. Individual data points and mean ± SEM are shown. ***Compared to control siRNA-treated cells using one-way ANOVA.

(C) Time-lapse images (collected at 0.5-s intervals) of GFP-IP_3R3 expressed in HLMVECs treated with control or EB3 siRNA and stimulated with α-thrombin (50 nM, as indicated). Times (s) are shown in each panel. Scale bar, 10 μm.

(D and E) Summary results (mean ± SEM from 11 cells in each group) show time course of GFP-IP_3R3 clustering after addition of α-thrombin (at t = 0) and (E) the lifetime of the clusters (time taken for 90% of clusters to disappear). These analyses were performed using processed images in which clusters present before addition of α-thrombin were subtracted (see Experimental Procedures). **Using Student's t test. Depletion of EB3 reduced both the number and lifetime of the IP_3R3 clusters. Figure S4 shows additional control experiments and related analyses of GFP-IP_3R2.

expressing GFP-IP_3R3(T804A) relative to those expressing GFP-IP_3R3 (Figure 4). GFP-IP_3R3(T804A) also formed fewer clusters than GFP-IP_3R3, they barely responded to α-thrombin, and the few clusters that formed were short lived (Figures 5A–5C). We conclude that EB3, via its interaction with IP_3R3 in endothelial cells, both dynamically regulates basal and agonist-evoked clustering of IP_3R3 and the IP_3-mediated Ca^{2+} release induced by α-thrombin (Figure 5D).

Loss of EB3 Suppresses Vascular Leakage In Vivo

Ca^{2+} signals, via both activation of protein kinase C α and myosin light chain (MLC) kinase, facilitate cell contraction and destabilization of AJs (Komarova and Malik, 2010; Vandenbroucke St Amant et al., 2012). We therefore examined the effects of EB3 and IP_3R3 on phosphorylation of MLC-II. We used human pulmonary artery endothelial cells (HPAECs) for these analyses because they are amenable to measurements of trans-endothelial electrical resistance (TEER), which directly report, in real time, changes in the integrity of AJs (Vandenbroucke St Amant et al., 2012; Szulcek et al., 2013). Depletion of EB3 or IP_3R3 (Figure S4C) suppressed α-thrombin-induced phosphorylation of MLC-II in HPAECs (Figures 6A and 6B). In confluent monolayers of HPAECs, α-thrombin induced a decrease in TEER, reflecting changes in cell shape and disruption of AJs between endothelial cells. The decrease in TEER was attenuated in cells lacking IP_3R3 or EB3 (Figures 6C and 6D), consistent with the lesser α-thrombin-evoked phosphorylation of MLC-II in these cells. These findings suggest that the interactions between EB3 and IP_3R3, through their effects on α-thrombin-induced Ca^{2+} signals, contribute to MLC-II activation and the increased permeability of the endothelial barrier.

Ca^{2+} signals in the endothelium play a critical role in regulating vascular permeability, a hallmark of acute lung injury and inflammation (Gandhirajan et al., 2013; Tauseef et al., 2012). To determine the role of EB3 in vascular endothelium, we generated EB3-*iECKO* mice in which high-fidelity inducible deletion of the EB3 gene (*Mapre3*) was restricted to endothelial cells (Figure S5). We then used lungs to determine the microvessel filtration coefficient ($k_{f,c}$), a measure of endothelial vascular permeability to liquid, in naive lungs and after activation of PAR-1 (Figure 6E). Lungs from control *Tie2-Cre*ER^{T2}-negative and EB3-*iECKO* mice had similar basal permeability, suggesting that EB3 is not essential in the adult microvasculature (Figure 6E). Infusion of a peptide agonist of PAR-1 (PAR1-AP) caused lung vascular hyper-permeability in wild-type, but not in EB3-*iECKO* mice (Figure 6E), indicating a pivotal role of EB3 in mediating the lung vascular permeability response.

DISCUSSION

Here, we show that stimulation of PLCβ as induced by the inflammatory mediator α-thrombin causes IP_3R3s to assemble into clusters in the ER membrane of endothelial cells and that this event requires the association of the TxIP motif of IP_3R3 with

OPEN
ACCESS
CellPress

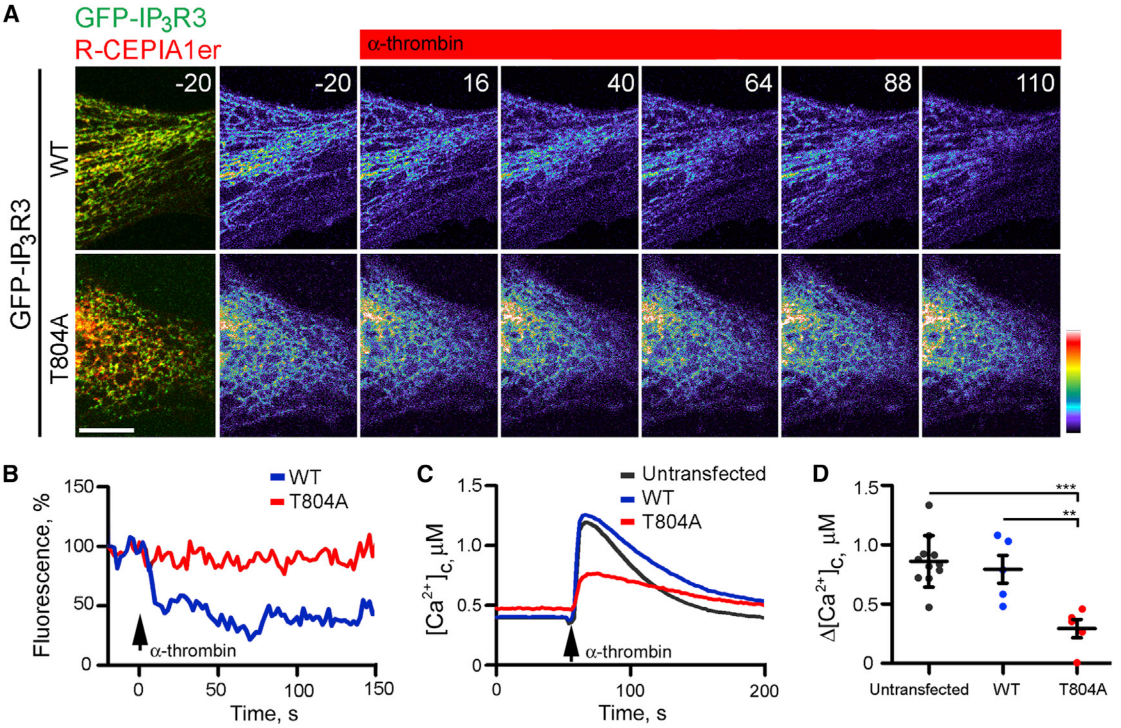

Figure 4. Interaction between IP_3R3 and EB3 Is Required for α-Thrombin-Induced Ca^{2+} Signaling

(A) Time-lapse images (times, in s, shown in panels) of HLMVEC expressing GFP-IP_3R3 (WT) or GFP-IP_3R3(T804A) mutant (green) with the ER luminal Ca^{2+} indicator, R-CEPIA1er (red) in overlaid images. R-CEPIA1er fluorescence is color coded with warm colors denoting high $[Ca^{2+}]_{ER}$. Stimulation with α-thrombin (50 nM at t = 0) in Ca^{2+}-free medium caused a decrease in $[Ca^{2+}]_{ER}$ in cells expressing wild-type (WT) GFP-IP_3R3, but not in cells expressing GFP-IP_3R3(T804A). Scale bar, 10 μm.

(B) Representative traces (normalized to 100% at 20 s before addition of α-thrombin) show $[Ca^{2+}]_{ER}$ monitored with R-CEPIA1er in individual cells expressing GFP-IP_3R3 or GFP-IP_3R3(T804A) and stimulated with α-thrombin (50 nM) in Ca^{2+}-free medium.

(C) Effects of α-thrombin (50 nM) on $[Ca^{2+}]_c$ recorded from ~5–10 fura-2-loaded HLMVECs expressing GFP-IP_3R3 (WT) or GFP-IP_3R3(T804A), or adjacent untransfected cells.

(D) Summary results show peak changes in $[Ca^{2+}]_c$ ($\Delta[Ca^{2+}]_c$) evoked by α-thrombin. Individual data points (four to ten cells) and means ± SEM are plotted. ***, **Using one-way ANOVA.

EB3 located at the tip of growing microtubules. Disrupting this interaction inhibits Ca^{2+} signaling, the phosphorylation of MLC-II, and the subsequent increase in endothelial permeability in response to α-thrombin.

The local density of IP_3Rs in the ER membrane induced by IP_3R clustering is thought to be responsible for the spatially organized nature of Ca^{2+} signaling in distinct cellular domains (Isshiki et al., 1998; Low et al., 2010). The spacing of IP_3Rs is key in determining whether the Ca^{2+} released stimulates the activity of neighboring IP_3Rs, and hence propagates Ca^{2+} signaling in a regenerative manner. Optimal clustering of IP_3Rs is therefore believed to be a critical factor in Ca^{2+} signaling (Shuai and Jung, 2003). A major factor determining IP_3R clustering may be IP_3-evoked conformational changes in IP_3Rs (Taufiq-Ur-Rahman et al., 2009; Tateishi et al., 2005). Studies also showed that microtubules facilitated movement of IP_3Rs in cells (Ferreri-Jacobia et al., 2005), and further that disruption of microtubules inhibited IP_3-evoked Ca^{2+} spiking (Béliveau and Guillemette, 2009; Fogarty et al., 2000), suggesting a role of microtubules in the mechanism of IP_3R clustering. However, Taufiq-Ur-Rahman et al. (2009) showed that clustering of IP_3Rs within isolated nuclei did not require microtubules. Thus, it has not been resolved whether microtubules regulate ER dynamics, and, if so, by what mechanisms and whether microtubule regulation of IP_3R clustering has functional relevance in an important Ca^{2+}-regulated biological response. Our results show the direct interaction of IP_3Rs with microtubules via the microtubule tip protein EB3 is required for the assembly of IP_3Rs into signaling clusters at the ER membrane, and these clusters mediate Ca^{2+} release from ER stores in endothelial cells. Our previous work established that IP_3-induced Ca^{2+} release in endothelial cells activated the phosphatase calcineurin, which dephosphorylated EB3, enabling EB3 dimerization. EB3 dimers, in turn, bound microtubules to promote persistent growth and disassembly of AJs (Komarova et al., 2012). The present work demonstrates that the microtubules functioning via EB3 are required for IP_3R clustering at the ER membrane and the genesis of intracellular Ca^{2+} signaling in endothelial cells.

The S/TxIP motif, located immediately downstream of the IP_3-binding site and conserved in all mammalian IP_3Rs, was required for IP_3Rs binding specifically to the C-terminal tail of EB3

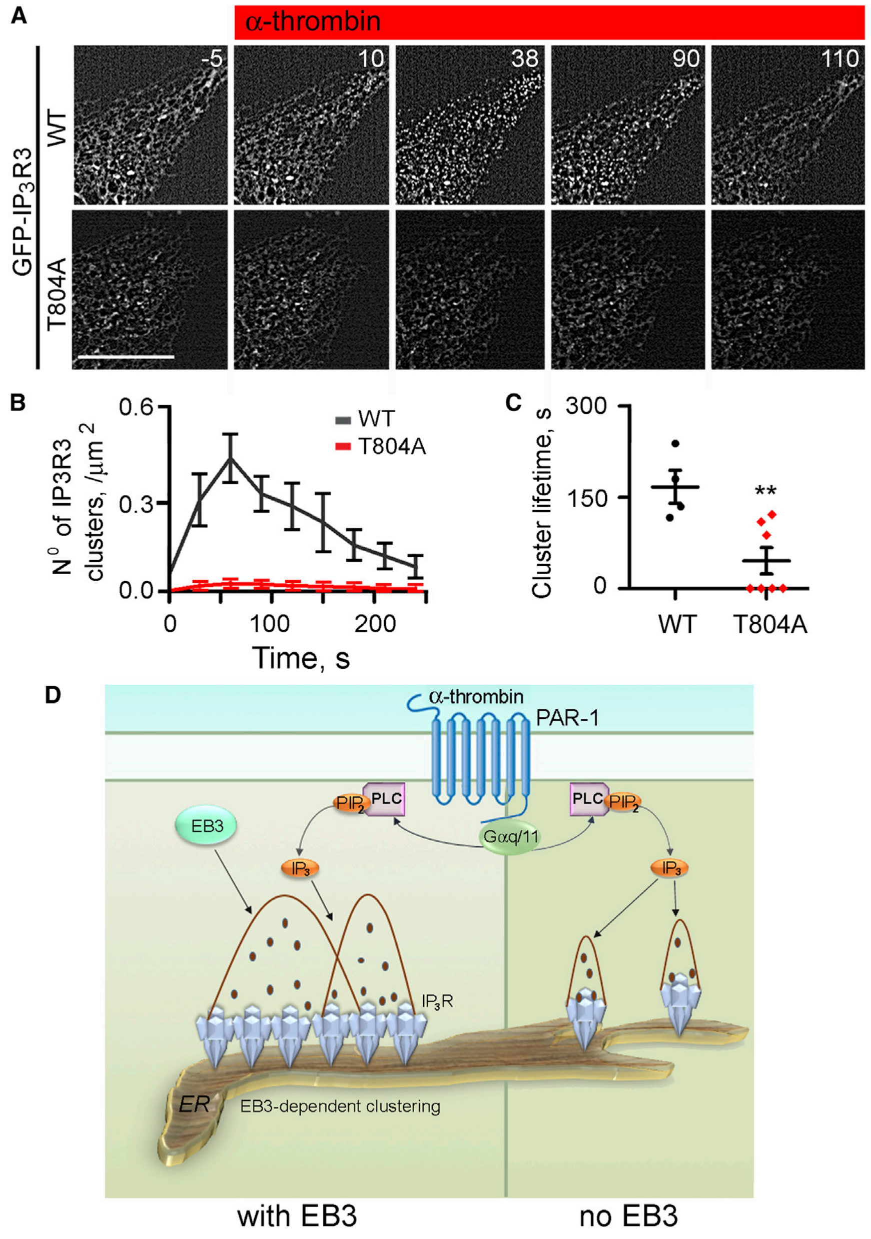

Figure 5. Interaction between IP_3R3 and EB3 Is Required for Effective IP_3R3 Clustering

(A) Time-lapse images (times, in s, shown in panels) of GFP fluorescence in HLMVECs expressing GFP-IP_3R3(WT) or GFP-IP_3R3(T804A) after stimulation with α-thrombin (50 nM, at t = 0). Scale bar, 10 μm.

(B and C) Summary results (mean ± SEM from five to seven cells) show the number of clusters for GFP-IP_3R3 and GFP-IP_3R3(T804A) (B) and cluster lifetime (C) after stimulation with α-thrombin (50 nM at t = 0). Data were analyzed using processed images as in Figures 3D and 3E. **Using Student's t test.

(D) Model for EB3-dependent IP_3R3 clustering and amplification of Ca^{2+} signals. EB3 facilitates clustering of IP_3Rs within ER membranes (left). α-Thrombin, via PAR-1, activates PLC and synthesis of IP_3. We propose that IP_3Rs more effectively release Ca^{2+} in response to this IP_3 when they have clustered, possibly because amplification of the signals by Ca^{2+}-induced Ca^{2+} release is more effective.

(as opposed to EB1) in endothelial cells. The enhanced binding of EB3 to IP_3R3 correlated with the requirement for EB3, rather than EB1, for α-thrombin-induced Ca^{2+} signaling. The requirement for EB3 may also result from its binding to additional, as-yet-unidentified proteins mediating clustering of IP_3R3s. Indeed, residues surrounding the S/TxIP motif have been recently shown to recognize EB3 (Leśniewska et al., 2014). Inhibition of α-thrombin-induced Ca^{2+} signals by a fragment of EB3 (Ct-mRFP) that dimerizes with native EB3 and prevents binding to microtubules confirmed that the requirement for EB3 is associated with its ability to bind to the growing microtubule tips. The concept of EB3 binding to IP_3R3 is further supported by our FRET analysis showing a close and specific apposition of IP_3R3 and EB3 at microtubule tips.

We found that IP_3R3 and IP_3R2 are the major IP_3R subtypes in endothelial cells. IP_3R2s are concentrated in perinuclear regions and clustered in unstimulated endothelial cells. The perinuclear distribution of IP_3R2 (Pantazaka and Taylor, 2011) and their propensity to cluster in unstimulated cells are also observed in other cells (Iwai et al., 2005; Sheppard et al., 1997). The latter finding suggests that basal levels of IP_3 are sufficient to stimulate IP_3R2 clustering, consistent with the greater affinity of IP_3R2 for IP_3 relative to other subtypes (Iwai et al., 2007). In unstimulated endothelial cells, however, IP_3R3s are more widely distributed and less clustered than IP_3R2s. The key role of IP_3R3 in mediating α-thrombin-induced Ca^{2+} signals is therefore probably due to their proximity to IP_3 produced by PLC at the plasma membrane. Our observation that α-thrombin augments the clustering of IP_3R3s is consistent with their lower sensitivity to IP_3 (Iwai et al., 2007). IP_3-induced clustering of IP_3R3s in the nuclear envelope did not require microtubules (Taufiq-Ur-Rahman et al., 2009), and yet in endothelial cells clustering requires EB3-mediated interaction of IP_3R3 with microtubules. The discrepancy may reflect a need for microtubules to facilitate movement of IP_3Rs within the crowded cytoplasm of intact cells and stabilize IP_3R clusters once they have formed.

We conclude that EB3-mediated tethering of IP_3R3s to microtubule tips in endothelial cells is required to assemble the Ca^{2+} signaling machinery. Moreover, α-thrombin stimulates formation of IP_3 at the plasma membrane, where it facilitates the clustering

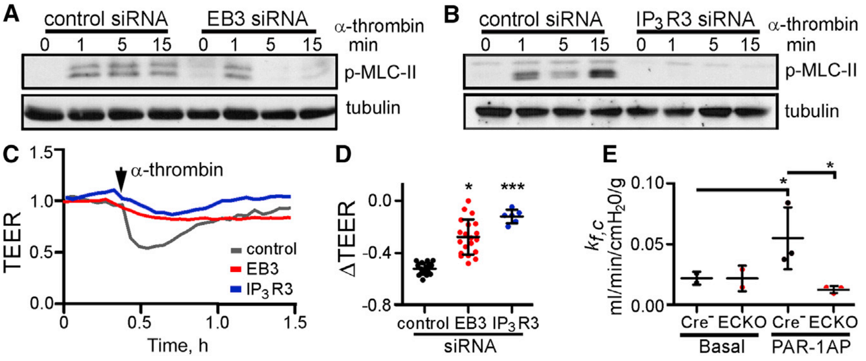

Figure 6. Depletion of EB3 Attenuates α-Thrombin-Induced Phosphorylation of MLC-II and Endothelial Hyper-permeability

(A and B) Time course of MLC-II phosphorylation in HPAECs treated with the indicated siRNAs and stimulated with α-thrombin (50 nM at t = 0). The western blots are typical of two to three experiments.

(C) Typical effects of α-thrombin (50 nM) on TEER in HPAECs treated with the indicated siRNAs. Basal resistance was normalized to 1.

(D) Summary results show the maximum change in TEER (ΔTEER) evoked by α-thrombin. Individual data points with mean ± SEM are shown, n = 6–20 per group, *, ***Compared to control siRNA-treated cells using one-way ANOVA.

(E) Permeability of endothelial vessel wall in lungs from EB3-*iECKO* and Cre-negative mice assessed by measuring microvascular filtration coefficient, $k_{f,c}$ (see Experimental Procedures). Isolated lungs were infused with 30 μM PAR-1 agonist peptide (PAR1-AP), and $k_{f,c}$ was calculated from the slope of the weight-gain curve between 15 and 20 min after the infusion. Individual data points are shown with mean ± SD, n = 3 mice per group; *From ANOVA. See also Figure S5.

of IP_3R3 via EB3-mediated interaction with microtubules. We propose that IP_3R3 clustering enables Ca^{2+}-mediated amplification of IP_3-induced Ca^{2+} release through activation of neighboring IP_3Rs. The IP_3-evoked Ca^{2+} signaling can therefore be attenuated in the absence of this amplification step. The physiological importance of the EB3-IP_3R3 interaction is evident from results in lung microvessels showing that endothelial cell-specific knockout of EB3 prevents the increase in endothelial permeability in response to inflammatory signal. Thus, microtubule-associated EB3 interacts directly with IP_3Rs to assemble the Ca^{2+} signaling complex at the ER membrane, and disruption of EB3-IP_3R interaction may be an attractive therapeutic target for vascular inflammation.

EXPERIMENTAL PROCEDURES

Materials

Sources of the expression constructs, antibodies, and siRNAs are provided in Supplemental Experimental Procedures. mRFP-(rat)CLIP-170 was generated from EGFP-CLIP-170 (Komarova et al., 2005) by substituting EGFP with monomeric RFP. For EB3-Ct-mRFP, the C terminus (residues 200–281) of EB3 was amplified by PCR and sub-cloned into the pmRFP-N1 vector (a gift from Dr. R. Tsien) at Sal1 and BamH1 sites. Expression constructs for the siRNA-insensitive form of EB3 and for EGFP-IP_3R3(T804A) were generated using the QuikChange Site-Directed Mutagenesis Kit (Agilent Technologies).

DAPI was from Sigma. Human α-thrombin was from Fisher Scientific. The PAR-1-activating peptide (PAR1-AP, TFLLRN-NH_2, ~90% purity) was synthesized by the Research Resources Core at UIC. Sources of other materials are provided in the relevant sections in Experimental Procedures.

Pull-Down Assays with EB Proteins

Preparation of bacterially expressed (His6)-tagged EB proteins and their covalent immobilization on Ni-NTA columns for pull-down experiments using lysates from HEK cells expressing GFP-tagged proteins are described in Supplemental Experimental Procedures.

Cell Culture and Transfections

HPAECs and HLMVECs (Lonza) were grown in EGM-2 medium supplemented with 10% fetal bovine serum (FBS) and EGM-2 MV Bulletkit or EGM-2 Bulletkit (Lonza), respectively. Cells were used between passages 2 and 6. CHO-K1 and HEK293 cells (ATCC) were grown in DMEM with 10% FBS (Gibco). Cells were transfected at ~80% confluence using X-tremeGENE HP according to the manufacturer's protocol (Roche) and used after 24–48 hr. For siRNA-mediated inhibition of protein expression, cells were treated with 70 nM siRNA (Supplemental Experimental Procedures) using GeneSilencer transfection reagent according to the manufacturer's protocol (Genlantis) and used after 72–96 hr. For experiments in which cells were stimulated with α-thrombin, the FBS concentration of the culture medium was reduced to 0.5% for 1 hr before the experiment.

Measurements of $[Ca^{2+}]_c$

HLMVECs grown on glass-bottomed dishes (Becton Dickinson) were loaded with fura-2 AM (3 μM, Life Technologies) for 20 min at 37°C in culture medium without supplements. The medium was then replaced with medium comprising: 150 mM NaCl, 4 mM KCl, 1 mM $MgCl_2$, 5.6 mM glucose, and 25 mM HEPES (pH 7.4), and, after ~10 min, cells were used for experiments at 25°C. Fura-2 fluorescence was excited at 340 and 380 nm and collected at 510 ± 80 nm using an Axiovert 100 inverted microscope (Carl Zeiss) equipped with Plan-Apo 60× with the numerical aperture (NA) 1.4 oil immersion objective, Lambda DG-4 switcher illumination system (Sutter Instruments), AxioCom Hsm camera (Zeiss), fura-2 filter set (Chroma), and AxioVision Physiology Acquisition module. Images were collected at 2-s intervals. Fluorescence ratios (F_{340}/F_{380}) were calculated within a circular region of interest (radius 3 μm) for each cell after subtraction of intracellular background fluorescence, determined by quenching fura-2 fluorescence by addition of 3 μM ionomycin with 5 mM $MnCl_2$. $[Ca^{2+}]_c$ was calculated from F_{340}/F_{380} ratios by reference to Ca^{2+} standard solutions (Life Technologies). Measurements of cytosolic IP_3 concentrations are described in Supplemental Experimental Procedures.

Measurements of $[Ca^{2+}]_{ER}$

The free $[Ca^{2+}]$ within the ER ($[Ca^{2+}]_{ER}$) was measured in HLMVECs transfected with the GEM-CEPIA1er ratiometric indicator (Suzuki et al., 2014). Analyses were performed at 37°C in the Ca^{2+}-free medium described above using a confocal microscope (Zeiss LSM 710, Axio Observer Z1) equipped with a 63× 1.4 NA Plan-Apochromat oil immersion objective and a diode-pumped solid-state laser. Cells were excited at 405 nm, and emission was collected by two photomultiplier tubes (456–476 and 510–530 nm). Images (5–10/cell) were collected at 1-s intervals, averaged over four frames to optimize signal-to-noise ratios, and fluorescence ratios ($R = F_{466}/F_{510}$) were calculated. For calibration, the plasma membrane was permeabilized (150 μM β-escin, 4 min), R_{min} and R_{max} were then determined in the absence of β-escin in medium with (R_{max}) or without (R_{min}) Ca^{2+}. The medium comprised: 140 mM KCl, 10 mM NaCl, 1 mM $MgCl_2$, 20 mM HEPES, 3 μM ionomycin, and 3 μM thapsigargin (pH 7.4), and either 0.3 mM EGTA (R_{min}) or 10 mM $CaCl_2$ (R_{max}). $[Ca^{2+}]_{ER}$ was then calculated from the observed fluorescence ratio (R):

$$[Ca^{2+}]_{ER} = K_D\left(\frac{R - R_{min}}{R_{max} - R}\right)^{\frac{1}{n}},$$

where n, Hill coefficient (1.37) and K_D for Ca^{2+} = 558 μM (Suzuki et al., 2014).

A confocal microscope (Zeiss, LSM 710, Axio Observer Z1) with BIG detector was used to capture dual-color images of m-Cherry-er with G-CEPIAer. For comparisons of relative changes in $[Ca^{2+}]_{ER}$ (Figure 1D), a cross-sectional view of the intensity values of G-CEPIAer fluorescence over time was generated, and the relative changes in $[Ca^{2+}]_{ER}$ were calculated.

Immunofluorescence and Live-Cell Imaging

HLMVECs were fixed with 4% formaldehyde, permeabilized with 0.2% Triton X-100, and immunostained for IP_3R2 or IP_3R3 (Supplemental Experimental Procedures). All z stack confocal images (Zeiss LSM 510 META) were acquired with the same settings to allow comparisons of IP_3R distributions between treatment groups. A projected image was generated by collecting a maximum voxel value through each z stack.

The methods used to quantify microtubule dynamics using EB1-GFP, assess the interactions between GFP-IP_3R3 and EB3-mRFP or mRFP-CLIP-170 using acceptor photobleaching, and to quantify clustering of GFP-IP_3R3 or immunostained IP_3R2 and IP_3R3 are described in Supplemental Experimental Procedures.

Trans-endothelial Electric Resistance Measurements

HPAECs were plated onto gelatin-coated 8W1E gold electrodes (Applied Biophysics), transfected with siRNA, and used after 72 hr (Garcia et al., 2011). Changes in trans-endothelial electric resistance (TEER) in response to α-thrombin were monitored using an Electric Cell Substrate Impedance Sensing system (Applied Biophysics) and normalized to the basal resistance.

Measurement of Vessel Filtration Coefficient (k_{fc})

Animal care and handling were performed according to an approved protocol of the University of Illinois at Chicago Animal Care Committee. The transgenic mice are described in Supplemental Experimental Procedures. For measurements of $k_{f,c}$, isolated lungs were perfused with RPMI medium at constant flow (2 ml/min), temperature (37°C), and venous pressure (4 cm H_2O) as previously described (Garcia et al., 2011). The preparation was ventilated at 120 breaths/min with constant peak inspiratory (~10 cm H_2O) and end expiratory pressures (2 cmH_2O). Lung weight change was recorded with a force-displacement transducer (Model FT03C, Grass Technologies). A 20-min equilibration perfusion established isogravimetric conditions, before measuring the gravimetric filtration coefficient ($k_{f,c}$) by comparing the rate of lung weight-gain during a baseline isogravimetric period with the rate after a change in hydrostatic pressure of at least 6 cm H_2O for 20 min. The filtration rate was determined from the slope of the weight-gain curve between 15 and 20 min after PAR1-AP infusion. $k_{f,c}$ was calculated from:

$$k_{fc} = \frac{\Delta W/\Delta t}{\Delta P},$$

where W, weight; t, time; P, pulmonary microvascular pressure.
Endothelial lysates were collected after each measurement of $k_{f,c}$ via a left atrial cannula perfused with buffer (50 mM Tris-Cl (pH 7.8), 0.2% Triton X-100, and protease and phosphatase inhibitor cocktails; 0.4 ml/min). Fractions were collected at 1-min intervals. Fractions 2 and 3, which were positive for VE-cadherin (endothelial marker) and negative for smooth muscle actin, were used for assessment of EB3 expression by western blot.

Statistical Analyses

Comparisons between groups were made using ANOVA with the Tukey post-test method or Student's t test. Significance values are shown by $^*p < 0.05$, $^{**}p < 0.01$, and $^{***}p < 0.001$.

SUPPLEMENTAL INFORMATION

Supplemental Information includes Supplemental Experimental Procedures, five figures, and seven tables and can be found with this article online at http://dx.doi.org/10.1016/j.celrep.2015.06.001.

AUTHOR CONTRIBUTIONS

Y.A.K. conceived the project; M.G., F.H., Y.S., S.M.V., and Y.A.K. conducted experiments; M.G., C.W.T., and Y.A.K. analyzed and interpreted data; M.G., C.W.T., A.B.M., and Y.A.K. wrote the paper.

ACKNOWLEDGMENTS

We thank the Core Imaging Facility of the Research Resources Center, University of Illinois at Chicago for use of microscopes. We thank Dr. A. Tanimura (Hokkaido, Japan) for LIBRAvIII and GFP-IP_3R3; Dr. M. Iino (Tokyo, Japan) for G-CEPIA1er, R-CEPIA1er, GEM-CEPIA1er, and mCherry-er; Dr. R. Tsien (UCSD, CA) for the pmRFP-N1 vector; Drs. P. Chambon (IGBMC, France) and S. Offermanns (Max Planck Institute, Germany) for the *tie2-Cre*ER^{T2} mouse line; Dr. S. Muallem (NIDCR, Bethesda) for YFP-STIM1; Dr. A. Akhmanova (Utrecht University, the Netherlands) for mRFP-CLIP-170. Supported by NIH grants R01 HL103922 and Giles F. Filley Memorial Award to Y.A.K.; PO1 HL60678 to A.B.M.; T32 HL07829-17 and AHA AWARD 13PRE17090090 to M.G., and a Wellcome Trust Senior Investigator Award (101844) to C.W.T. The work presented fulfills in part the PhD degree requirement for M.G. Y.A.K., S.M.V., and A.B.M. have a patent that is potentially related to this study.

Received: May 2, 2014
Revised: March 27, 2015
Accepted: May 31, 2015
Published: June 25, 2015

REFERENCES

Akhmanova, A., and Steinmetz, M.O. (2010). Microtubule +TIPs at a glance. J. Cell Sci. *123*, 3415–3419.

Béliveau, E., and Guillemette, G. (2009). Microfilament and microtubule assembly is required for the propagation of inositol trisphosphate receptor-induced Ca^{2+} waves in bovine aortic endothelial cells. J. Cell. Biochem. *106*, 344–352.

Chalmers, M., Schell, M.J., and Thorn, P. (2006). Agonist-evoked inositol trisphosphate receptor (IP_3R) clustering is not dependent on changes in the structure of the endoplasmic reticulum. Biochem. J. *394*, 57–66.

Daneshjou, N., Sieracki, N., van Nieuw Amerongen, G.P., Conway, D.E., Schwartz, M.A., Komarova, Y.A., and Malik, A.B. (2015). Rac1 functions as a reversible tension modulator to stabilize VE-cadherin trans-interaction. J. Cell Biol. *208*, 23–32.

Dejana, E. (2004). Endothelial cell-cell junctions: happy together. Nat. Rev. Mol. Cell Biol. *5*, 261–270.

Ferreri-Jacobia, M., Mak, D.O., and Foskett, J.K. (2005). Translational mobility of the type 3 inositol 1,4,5-trisphosphate receptor Ca^{2+} release channel in endoplasmic reticulum membrane. J. Biol. Chem. *280*, 3824–3831.

Fogarty, K.E., Kidd, J.F., Turner, A., Skepper, J.N., Carmichael, J., and Thorn, P. (2000). Microtubules regulate local Ca^{2+} spiking in secretory epithelial cells. J. Biol. Chem. *275*, 22487–22494.

Foskett, J.K., White, C., Cheung, K.H., and Mak, D.O. (2007). Inositol trisphosphate receptor Ca^{2+} release channels. Physiol. Rev. *87*, 593–658.

Friedman, J.R., and Voeltz, G.K. (2011). The ER in 3D: a multifunctional dynamic membrane network. Trends Cell Biol. *21*, 709–717.

Gandhirajan, R.K., Meng, S., Chandramoorthy, H.C., Mallilankaraman, K., Mancarella, S., Gao, H., Razmpour, R., Yang, X.F., Houser, S.R., Chen, J., et al. (2013). Blockade of NOX2 and STIM1 signaling limits lipopolysaccharide-induced vascular inflammation. J. Clin. Invest. *123*, 887–902.

Garcia, A.N., Vogel, S.M., Komarova, Y.A., and Malik, A.B. (2011). Permeability of endothelial barrier: cell culture and in vivo models. Methods Mol. Biol. *763*, 333–354.

Giannotta, M., Trani, M., and Dejana, E. (2013). VE-cadherin and endothelial adherens junctions: active guardians of vascular integrity. Dev. Cell *26*, 441–454.

Graier, W.F., Paltauf-Doburzynska, J., Hill, B.J., Fleischhacker, E., Hoebel, B.G., Kostner, G.M., and Sturek, M. (1998). Submaximal stimulation of porcine endothelial cells causes focal Ca^{2+} elevation beneath the cell membrane. J. Physiol. *506*, 109–125.

Grigoriev, I., Gouveia, S.M., van der Vaart, B., Demmers, J., Smyth, J.T., Honnappa, S., Splinter, D., Steinmetz, M.O., Putney, J.W., Jr., Hoogenraad, C.C., and Akhmanova, A. (2008). STIM1 is a MT-plus-end-tracking protein involved in remodeling of the ER. Curr. Biol. *18*, 177–182.

Honnappa, S., Gouveia, S.M., Weisbrich, A., Damberger, F.F., Bhavesh, N.S., Jawhari, H., Grigoriev, I., van Rijssel, F.J., Buey, R.M., Lawera, A., et al. (2009). An EB1-binding motif acts as a microtubule tip localization signal. Cell *138*, 366–376.

Howard, J., and Hyman, A.A. (2003). Dynamics and mechanics of the microtubule plus end. Nature *422*, 753–758.

Isshiki, M., Ando, J., Korenaga, R., Kogo, H., Fujimoto, T., Fujita, T., and Kamiya, A. (1998). Endothelial Ca^{2+} waves preferentially originate at specific loci in caveolin-rich cell edges. Proc. Natl. Acad. Sci. USA *95*, 5009–5014.

Iwai, M., Tateishi, Y., Hattori, M., Mizutani, A., Nakamura, T., Futatsugi, A., Inoue, T., Furuichi, T., Michikawa, T., and Mikoshiba, K. (2005). Molecular cloning of mouse type 2 and type 3 inositol 1,4,5-trisphosphate receptors and identification of a novel type 2 receptor splice variant. J. Biol. Chem. *280*, 10305–10317.

Iwai, M., Michikawa, T., Bosanac, I., Ikura, M., and Mikoshiba, K. (2007). Molecular basis of the isoform-specific ligand-binding affinity of inositol 1,4,5-trisphosphate receptors. J. Biol. Chem. *282*, 12755–12764.

Komarova, Y., and Malik, A.B. (2010). Regulation of endothelial permeability via paracellular and transcellular transport pathways. Annu. Rev. Physiol. *72*, 463–493.

Komarova, Y., Lansbergen, G., Galjart, N., Grosveld, F., Borisy, G.G., and Akhmanova, A. (2005). EB1 and EB3 control CLIP dissociation from the ends of growing microtubules. Mol. Biol. Cell *16*, 5334–5345.

Komarova, Y., De Groot, C.O., Grigoriev, I., Gouveia, S.M., Munteanu, E.L., Schober, J.M., Honnappa, S., Buey, R.M., Hoogenraad, C.C., Dogterom, M., et al. (2009). Mammalian end binding proteins control persistent microtubule growth. J. Cell Biol. *184*, 691–706.

Komarova, Y.A., Huang, F., Geyer, M., Daneshjou, N., Garcia, A., Idalino, L., Kreutz, B., Mehta, D., and Malik, A.B. (2012). VE-cadherin signaling induces EB3 phosphorylation to suppress microtubule growth and assemble adherens junctions. Mol. Cell *48*, 914–925.

Leśniewska, K., Warbrick, E., and Ohkura, H. (2014). Peptide aptamers define distinct EB1- and EB3-binding motifs and interfere with microtubule dynamics. Mol. Biol. Cell *25*, 1025–1036.

Low, J.T., Shukla, A., Behrendorff, N., and Thorn, P. (2010). Exocytosis, dependent on Ca^{2+} release from Ca^{2+} stores, is regulated by Ca^{2+} microdomains. J. Cell Sci. *123*, 3201–3208.

Maurer, S.P., Fourniol, F.J., Bohner, G., Moores, C.A., and Surrey, T. (2012). EBs recognize a nucleotide-dependent structural cap at growing microtubule ends. Cell *149*, 371–382.

Mitsuyama, F., and Sawai, T. (2001). The redistribution of Ca^{2+} stores with inositol 1,4,5-trisphosphate receptor to the cleavage furrow in a microtubule-dependent manner. Int. J. Dev. Biol. *45*, 861–868.

Pantazaka, E., and Taylor, C.W. (2011). Differential distribution, clustering, and lateral diffusion of subtypes of the inositol 1,4,5-trisphosphate receptor. J. Biol. Chem. *286*, 23378–23387.

Pendin, D., McNew, J.A., and Daga, A. (2011). Balancing ER dynamics: shaping, bending, severing, and mending membranes. Curr. Opin. Cell Biol. *23*, 435–442.

Pozo-Guisado, E., Campbell, D.G., Deak, M., Alvarez-Barrientos, A., Morrice, N.A., Alvarez, I.S., Alessi, D.R., and Martín-Romero, F.J. (2010). Phosphorylation of STIM1 at ERK1/2 target sites modulates store-operated calcium entry. J. Cell Sci. *123*, 3084–3093.

Ribeiro, C.M., Reece, J., and Putney, J.W., Jr. (1997). Role of the cytoskeleton in calcium signaling in NIH 3T3 cells. An intact cytoskeleton is required for agonist-induced $[Ca^{2+}]_i$ signaling, but not for capacitative calcium entry. J. Biol. Chem. *272*, 26555–26561.

Sheppard, C.A., Simpson, P.B., Sharp, A.H., Nucifora, F.C., Ross, C.A., Lange, G.D., and Russell, J.T. (1997). Comparison of type 2 inositol 1,4,5-trisphosphate receptor distribution and subcellular Ca^{2+} release sites that support Ca^{2+} waves in cultured astrocytes. J. Neurochem. *68*, 2317–2327.

Shuai, J.W., and Jung, P. (2003). Optimal ion channel clustering for intracellular calcium signaling. Proc. Natl. Acad. Sci. USA *100*, 506–510.

Smith, I.F., Wiltgen, S.M., and Parker, I. (2009). Localization of puff sites adjacent to the plasma membrane: functional and spatial characterization of Ca^{2+} signaling in SH-SY5Y cells utilizing membrane-permeant caged IP_3. Cell Calcium *45*, 65–76.

Suzuki, J., Kanemaru, K., Ishii, K., Ohkura, M., Okubo, Y., and Iino, M. (2014). Imaging intraorganellar Ca^{2+} at subcellular resolution using CEPIA. Nat. Commun. *5*, 4153.

Szulcek, R., Beckers, C.M., Hodzic, J., de Wit, J., Chen, Z., Grob, T., Musters, R.J., Minshall, R.D., van Hinsbergh, V.W., and van Nieuw Amerongen, G.P. (2013). Localized RhoA GTPase activity regulates dynamics of endothelial monolayer integrity. Cardiovasc. Res. *99*, 471–482.

Takei, K., Shin, R.M., Inoue, T., Kato, K., and Mikoshiba, K. (1998). Regulation of nerve growth mediated by inositol 1,4,5-trisphosphate receptors in growth cones. Science *282*, 1705–1708.

Tanimura, A., Morita, T., Nezu, A., Shitara, A., Hashimoto, N., and Tojyo, Y. (2009). Use of fluorescence resonance energy transfer-based biosensors for the quantitative Aanalysis of inositol 1,4,5-trisphosphate dynamics in calcium oscillations. J. Biol. Chem. *284*, 8910–8917.

Tasaka, K., Mio, M., Fujisawa, K., and Aoki, I. (1991). Role of microtubules on Ca^{2+} release from the endoplasmic reticulum and associated histamine release from rat peritoneal mast cells. Biochem. Pharmacol. *41*, 1031–1037.

Tateishi, Y., Hattori, M., Nakayama, T., Iwai, M., Bannai, H., Nakamura, T., Michikawa, T., Inoue, T., and Mikoshiba, K. (2005). Cluster formation of inositol 1,4,5-trisphosphate receptor requires its transition to open state. J. Biol. Chem. *280*, 6816–6822.

Taufiq-Ur-Rahman, Skupin, A., Falcke, M., and Taylor, C.W. (2009). Clustering of InsP3 receptors by InsP3 retunes their regulation by InsP3 and Ca2+. Nature *458*, 655–659.

Tauseef, M., Knezevic, N., Chava, K.R., Smith, M., Sukriti, S., Gianaris, N., Obukhov, A.G., Vogel, S.M., Schraufnagel, D.E., Dietrich, A., et al. (2012). TLR4 activation of TRPC6-dependent calcium signaling mediates endotoxin-induced lung vascular permeability and inflammation. J. Exp. Med. *209*, 1953–1968.

Taylor, C.W., Tovey, S.C., Rossi, A.M., Lopez Sanjurjo, C.I., Prole, D.L., and Rahman, T. (2014). Structural organization of signalling to and from IP_3 receptors. Biochem. Soc. Trans. *42*, 63–70.

Tran, C.H., Kurjiaka, D.T., and Welsh, D.G. (2014). Emerging trend in second messenger communication and myoendothelial feedback. Front Physiol *5*, 243.

Vandenbroucke St Amant, E., Tauseef, M., Vogel, S.M., Gao, X.P., Mehta, D., Komarova, Y.A., and Malik, A.B. (2012). PKCα activation of p120-catenin serine 879 phospho-switch disassembles VE-cadherin junctions and disrupts vascular integrity. Circ. Res. *111*, 739–749.

Vermassen, E., Van Acker, K., Annaert, W.G., Himpens, B., Callewaert, G., Missiaen, L., De Smedt, H., and Parys, J.B. (2003). Microtubule-dependent redistribution of the type-1 inositol 1,4,5-trisphosphate receptor in A7r5 smooth muscle cells. J. Cell Sci. *116*, 1269–1277.

Vogel, S.M., and Malik, A.B. (2012). Cytoskeletal dynamics and lung fluid balance. Compr Physiol *2*, 449–478.

Wainwright, M.S., Rossi, J., Schavocky, J., Crawford, S., Steinhorn, D., Velentza, A.V., Zasadzki, M., Shirinsky, V., Jia, Y., Haiech, J., et al. (2003). Protein kinase involved in lung injury susceptibility: evidence from enzyme isoform genetic knockout and in vivo inhibitor treatment. Proc. Natl. Acad. Sci. USA *100*, 6233–6238.

Wilson, B.S., Pfeiffer, J.R., Smith, A.J., Oliver, J.M., Oberdorf, J.A., and Wojcikiewicz, R.J. (1998). Calcium-dependent clustering of inositol 1,4,5-trisphosphate receptors. Mol. Biol. Cell *9*, 1465–1478.

Wu, M.M., Covington, E.D., and Lewis, R.S. (2014). Single-molecule analysis of diffusion and trapping of STIM1 and Orai1 at endoplasmic reticulum-plasma membrane junctions. Mol. Biol. Cell *25*, 3672–3685.

Zhang, X.F., and Forscher, P. (2009). Rac1 modulates stimulus-evoked Ca($^{2+}$) release in neuronal growth cones via parallel effects on microtubule/endoplasmic reticulum dynamics and reactive oxygen species production. Mol. Biol. Cell *20*, 3700–3712.

Cell Reports

Supplemental Information

Microtubule-Associated Protein EB3 Regulates IP3 Receptor Clustering and Ca2+ Signaling in Endothelial Cells

Melissa Geyer, Fei Huang, Ying Sun, Stephen M. Vogel, Asrar B. Malik, Colin W. Taylor, and Yulia A. Komarova

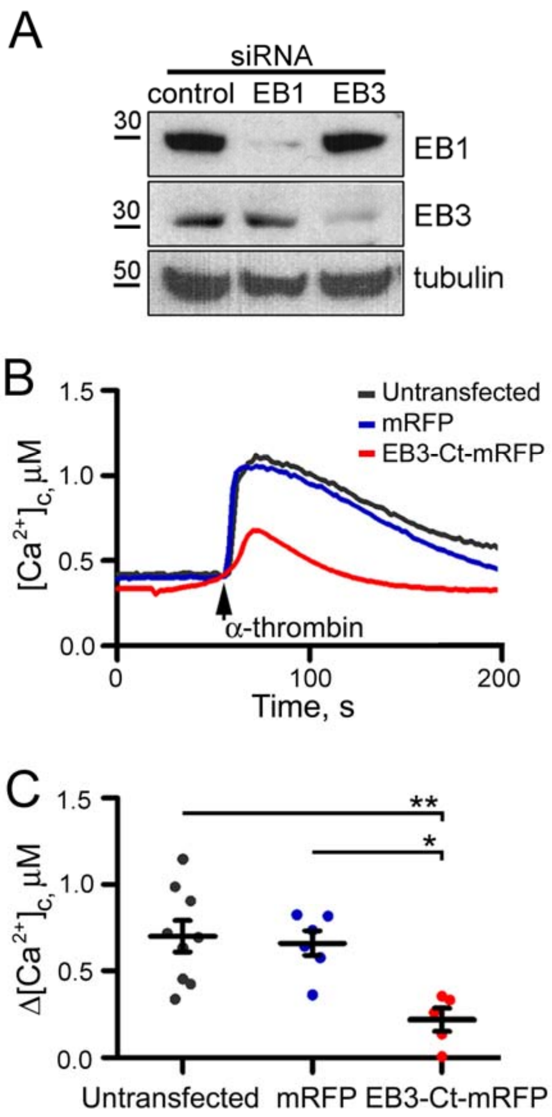

Figure S1. Attenuation of α-Thrombin-Evoked Ca^{2+} Signals by Loss of EB3. Related to Figure 1.

(A) Western blot using antibodies to EB1, EB3 and tubulin shows effects of the indicated siRNA treatments. M_r markers (kDa) are shown.

(B) Cytosolic Ca^{2+} signals evoked by addition of α-thrombin (50 nM) in Ca^{2+}-free medium recorded from HLMVECs expressing mRFP, a C-terminal fragment of EB3 tagged with mRFP (which has a dominant-negative effect by dimerizing with endogenous EBs and displacing them from microtubule plus ends) or from neighboring untransfected cells.

(C) Summary results show peak changes in $[Ca^{2+}]_c$ evoked by α-thrombin ($\Delta[Ca^{2+}]_c$). Individual data points are shown with mean ± SEM (n = 5-9 experiments). *, **, compared to untransfected cells using one-way ANOVA.

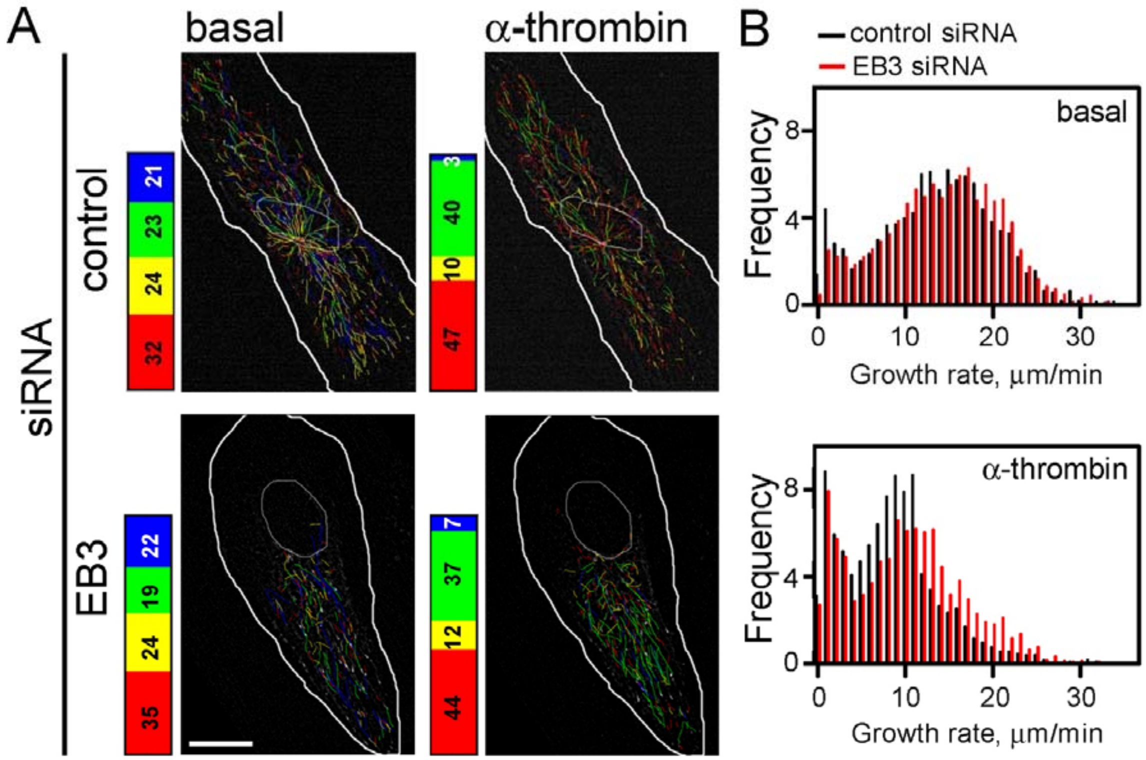

Figure S2. Loss of EB3 Does Not Affect Microtubule Dynamics in Unstimulated Cells or Within 5 min of Stimulation With α-Thrombin. Related to Table 1.

(A) Microtubule growth tracks classified by growth speed and lifetime in control and EB3 siRNA-treated cells before and then 5 min after addition of α-thrombin (50 nM). Blue, fast long-lived growth; green, slow long-lived growth; yellow, fast short-lived growth; red, slow short-lived growth (see Supplemental Experimental Procedures). Typical distributions of classified tracks and the percentage of tracks within each of the four categories (bars alongside each cell) are shown. Scale bar, 10 μm.

(B) Summary results (1907-2534 tracks, with 7-8 cells/group) show instantaneous microtubule growth rates for cells treated with control or EB3 siRNA before and within 5 min of stimulation with α-thrombin (50 nM). The percentage of tracks displaying each growth rate is shown.

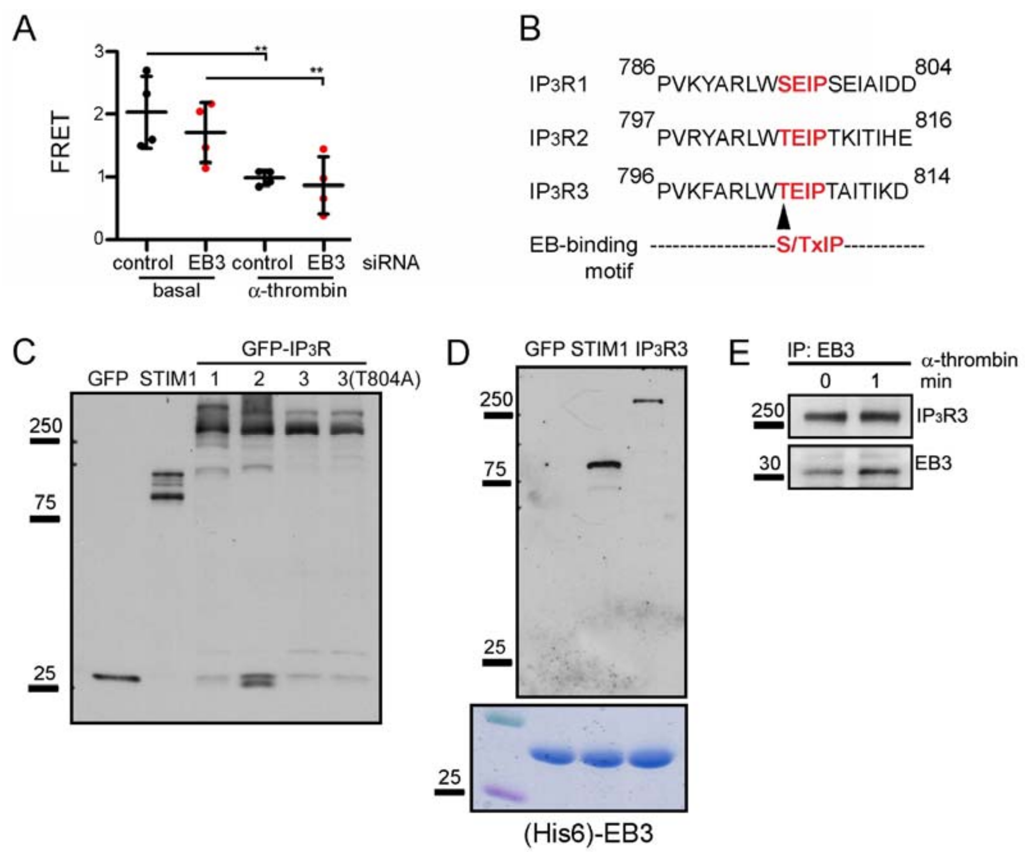

Figure S3. Association of IP$_3$ Receptors With EB3. Related to Figure 2.
(A) Cytosolic concentrations of IP$_3$ were measured in HLMVEC monolayers stimulated with α-thrombin (50 nM) using the FRET-based sensor LIBRAvIII (see Supplemental Experimental Procedures), where a decrease in FRET denotes an increase in IP$_3$ concentration. Individual data points and mean ± SEM (n = 4 cells) show the fluorescence emission ratio (FRET) (see Supplemental Experimental Procedures) measured 30 s after addition of α-thrombin to cells treated with control or EB3 siRNA. The results demonstrate that basal and α-thrombin-stimulated IP$_3$ levels were unaffected by loss of EB3. **, compared to basal level in the matched siRNA group, using one-way ANOVA.
(B) Sequence alignment of human IP$_3$Rs and EB-binding motif. Arrow designates T804 in IP$_3$R3.
(C) Western blot (using antibody to GFP) of GFP, STIM1-GFP and GFP-IP$_3$Rs in the lysates from HEK cells used for the pull-down experiments shown in Figures 2B and 2C.
(D) Pull-down of GFP, STIM1-GFP and GFP-IP$_3$R3 from lysates of HEK cells with (His6)-EB3, probed with an antibody to GFP. Lower panel shows Coomassie Brilliant Blue-stained gels loaded with 5% of the (His6)-EB3 used for the pull-down. Results (C and D) are typical of 2 experiments. M_r markers (kDa) are shown alongside each gel.
(E) An anti-EB3 antibody was used for immunoprecipitation (IP) of endogenous EB3 in lysates prepared from HPAECs before and 1 min after addition of α-thrombin. The western blot of the IP shows endogenous EB3 and IP$_3$R3. Results are typical of 2 experiments. M_r markers (kDa) are shown alongside the blot.

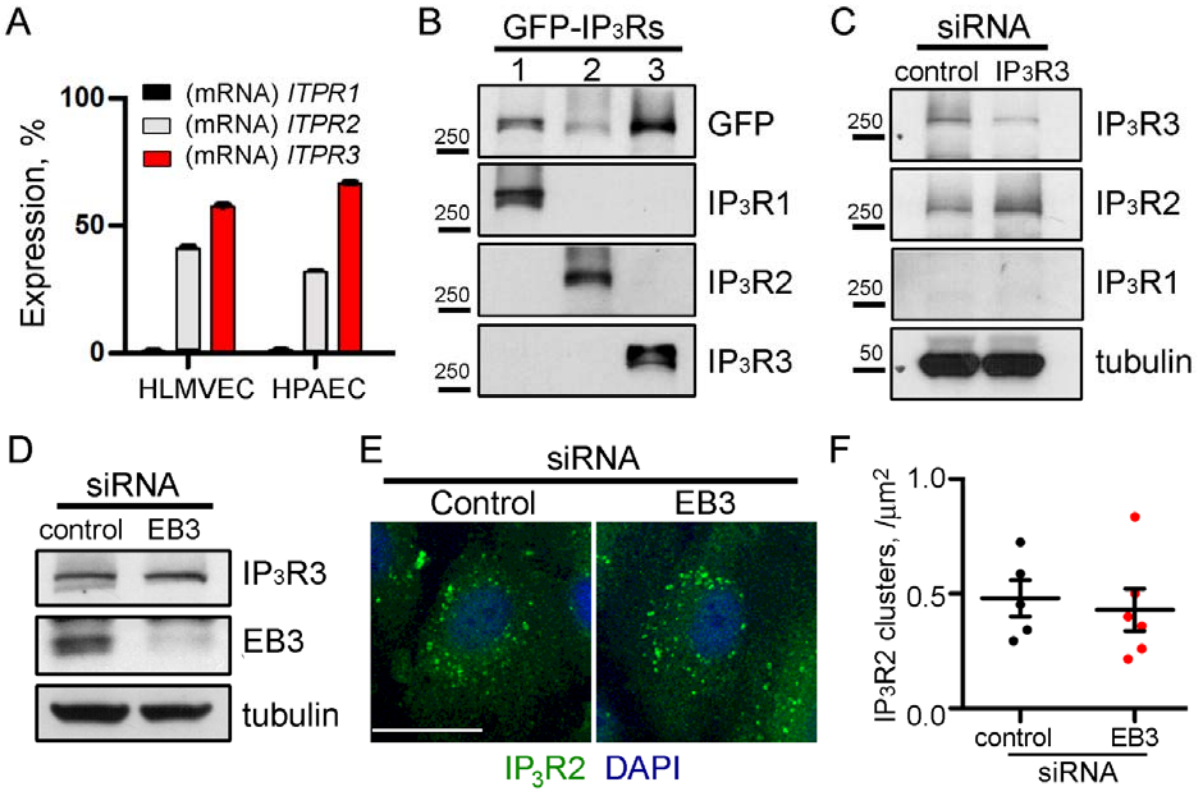

Figure S4. Loss of EB3 Does Not Affect Expression of IP$_3$R3 or Clustering of IP$_3$R2s. Related to Figure 3.

(A) Expression of IP$_3$R subtypes in human pulmonary artery endothelial cells (HPAECs) and HLMVECs determined by QPCR. Results (means ± SD, n = 2) show the percentage of mRNA for each IP$_3$R subtype.

(B) Characterization of antibodies to IP$_3$R1 and IP$_3$R2. Lysates of HEK cells overexpressing GFP-IP$_3$R1, GFP-IP$_3$R2 or GFP-IP$_3$R3 were used for western blots with rabbit polyclonal antibodies for IP$_3$R1 and IP$_3$R2, and mouse monoclonal antibody for IP$_3$R3 (see tables in Supplemental Experimental Procedures). The results (because the over-expressed GFP-IP$_3$Rs are much more abundant than endogenous IP$_3$Rs) confirm the subtype-specificity of the antibodies.

(C) Expression of IP$_3$R subtypes in HLMVECs assessed by western blot analysis. Only IP$_3$R2 and IP$_3$R3 were detected in HLMVECs. Depletion of IP$_3$R3 with siRNA did not cause upregulation of IP$_3$R1. Results are typical of 2 independent experiments. M_r markers (kDa) are shown alongside the blots.

(D) Loss of EB3 does not affect IP$_3$R3 expression. Western blot analysis of HPAECs treated with control or EB3 siRNA and probed for EB3 and IP$_3$R3; tubulin is a loading control. Typical results of 4 independent experiments.

(E) Distribution of endogenous IP$_3$R2 in HLMVECs treated with control and EB3 siRNA. Nuclei are stained with DAPI. Scale bar, 10 µm.

(F) Quantification of the numbers of IP$_3$R2 clusters demonstrates that loss of EB3 does not affect IP$_3$R2 clustering. Individual points with mean ± SEM are shown, n = 5-6 images per group.

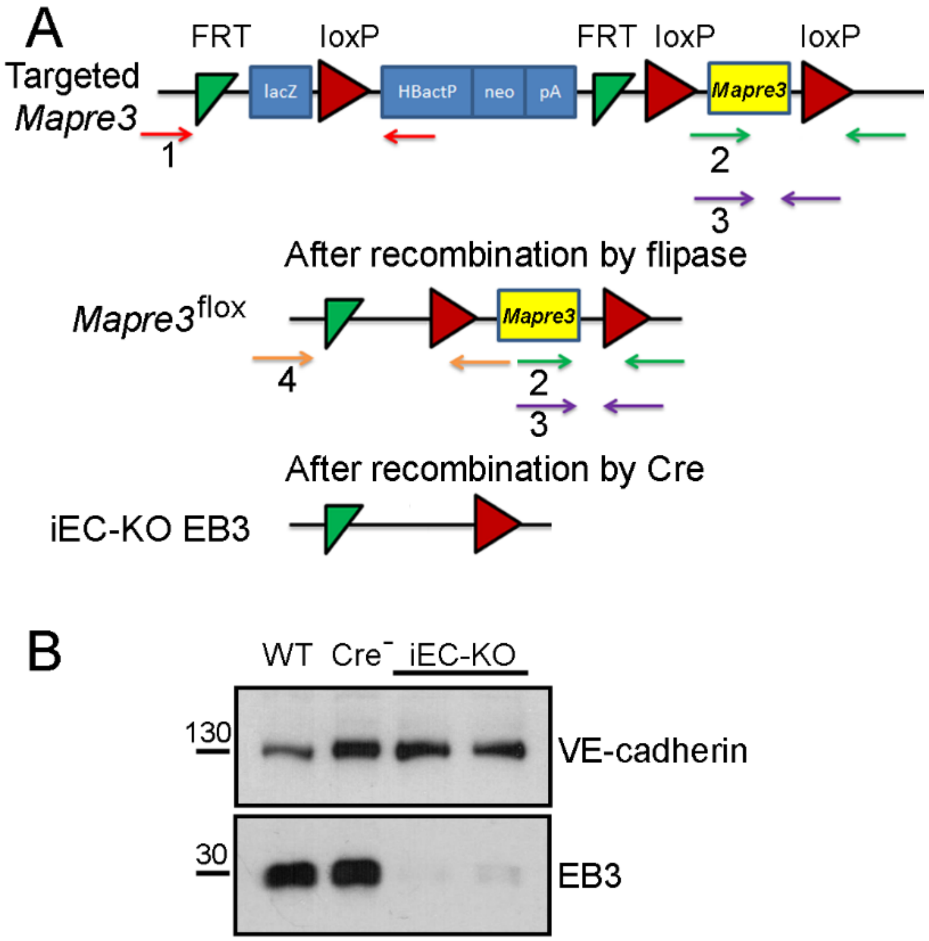

Figure S5. Generation of Mouse Model for Tamoxifen-Inducible Deletion of *mapre3* Gene in Endothelium. Related to Figure 6.

(A) Schematic representation of inserted transgenes and two-step protocol for generating inducible endothelium-specific (iEC*)* knockout (KO) of EB3. *Mapre3*-loxP-targeted transgenic mice were generated by flanking the *Mapre3* gene with loxP sites; a promoter-driving selection cassette (L1L2-Bact-P) was flanked by FRT sites and inserted upstream of the *Mapre3* critical exon. The L1L2-Bact-P cassette was removed by crossing mice with flipase-mediated recombination (Gt(ROSA)-26Sortm2(FLP*)Sor mice) generating *Mapre3*$^{flox/flox}$ mice. Ubiquitous deletion of EB3 in endothelium was achieved by crossing *Mapre3*$^{flox/flox}$ to *Tie2-Cre*ER^{T2} and upon stimulation of *Cre* recombinase with tamoxifen.

(B) Deletion of EB3 in endothelium was confirmed by probing lung endothelium-specific fractions (collected from lung after each $k_{f,c}$ trial, see Experimental Procedures) for VE-cadherin and EB3 on western blots. Wild-type (WT), C57/B6 control untreated mouse; *Cre*$^{-}$, *Mapre3*$^{flox/flox}$ mouse treated with tamoxifen; iECKO, *Mapre3*$^{flox/flox}$ -*Tie2-Cre*ER^{T2} mice treated with tamoxifen.

SUPPLEMENTAL EXPERIMENTAL PROCEDURES

Expression of EB Proteins and Pull-Down Assays

Preparation of (His6)-tagged EB1 and EB3-Ct was described previously (Komarova et al, 2009). (His6)-TEV-EB3 and (His6)-TEV-EB3ΔAc were generated by PCR and sub-cloned into the modified pRSF-Duet-1 vector (EMD Millipore) at BamH1 and HindIII sites. Details of these, and the other constructs used, are provided in the supplemental table S1 and S2, *Expression Constructs Used*.

(His6)-tagged proteins (Figure 2A) were expressed in *Escherichia coli* strain BL21 (DE3) (Stratagene). Bacteria were grown at 37°C in LB medium containing 50 µg/ml kanamycin. When the OD_{600} reached 0.6-0.7, protein synthesis was induced by addition of isopropyl 1-thio-β-D-galactopyranoside (IPTG) to a final concentration of 250 µM. After 4 h at 30°C, bacterial pellets were isolated and sonicated (4 x 1 min) in medium comprising 150 mM NaCl, 5 mM 2-mercaptoethanol, 2 mM $CaCl_2$, 10 mM imidazole, 2 mM PMSF, 25 mM Tris, pH 7.4.

(His6)-EB proteins were purified using Ni-NTA beads (Thermo Scientific) and then covalently attached to them. Ni-NTA beads (1 ml) in a 20 ml column (Bio-Rad) were equilibrated with 50 bed-volumes of binding buffer (25 mM Tris, pH 7.4, 300 mM NaCl, 5 mM 2-mercaptoethanol, 2 mM PMSF). Bacterial lysate (50 ml) was then added to the column, followed by washing (150 bed-volumes of wash buffer, ~75 ml). The protein-bound beads were washed with phosphate-buffered saline (PBS) supplemented with 2 mM $CaCl_2$ and protease inhibitor cocktail (Sigma) and stored in the same medium. A sample of the beads was heated in sample buffer at 95°C for 5 min and SDS-PAGE was used to determine the purity of the (His6)-EB proteins (typically 90%). The concentration of EB protein was determined from densitometric analysis of Coomassie Brilliant Blue staining with BSA as a standard. For cross-linking of (His6)-EB to the Ni-NTA beads, 40-50 µl of (His6)-tagged proteins bound to beads were incubated with N-hydroxysuccinimide (75 mM) and 1-ethyl-3-carbodiimide (50 mM) in 20 mM Hepes buffer, pH ~7.0 (1 ml for 1 h at 4°C). The beads were washed with PBS (2 x 1 ml) and incubated with PBS containing 5% BSA for 1 h to block non-specific binding before use for pull-downs.

For pull-downs analyses, HEK 293 cells expressing GFP-tagged proteins were lysed in medium comprising 150 mM NaCl, 50 mM Tris, pH 8.0, 1% NP40 (Sigma), and 1% protease inhibitor cocktail (Sigma). The lysates (~300 µl/sample) containing equal amounts of GFP-tagged proteins (Figure S3) were incubated with the (His6)-EB/Ni-NTA beads (50 µl) in the presence of 10 mM imidazole at 4°C for 2 h. Bound proteins were eluted by heating in sample buffer at 95°C for 5 min before SDS-PAGE using 4-20% tris-glycine gels (Life Technologies). After transfer to a nitrocellulose membrane (Bio-Rad), the blots were probed with anti-GFP antibodies. Details of the antibodies used for these, and all other experiments with antibodies, are provided in the supplemental tables S3 and S4, *Primary Antibodies Used*, and *Secondary Antibodies Used*.

QPCR Analysis of mRNA Expression

Total RNA was isolated using RNeasy mini Kit (Qiagen), quantified (absorbance at 260 nm) and reverse transcribed using iScript according to the manufacturer's instructions (BioRad). The cDNA was amplified by real-time PCR using SYBR® Green Master Mix gene expression assay (Integrated DNA Technologies) and the primers listed in the supplemental table S6, *Primers Used*.

For each QPCR, amplification efficiency (E) was calculated as 10^m, where m is the mean increase in fluorescence after the cycle threshold (C_T) as previously described (Govindan et al., 2010). Expression levels of *ITPR*s were calculated relative to glucose-6-phosphate dehydrogenase (G6PD), a housekeeping product:

$$\frac{E^{C_T(ITPR)}}{E^{C_T(G6PD)}}$$

Each QPCR reaction was performed in duplicate with samples from three different wells.

FRET Analysis of EB3 Interactions with IP_3Rs

FRET between GFP-IP_3R3 and EB3-mRFP or mRFP-CLIP-170 in CHO-K1 cells was assessed by measuring the recovery of donor (GFP) fluorescence after acceptor (mRFP) photo-bleaching. A selected region (~0.5 μm across) across the anticipated path of microtubule growth (predicted from the linear trajectory of the growing microtubule) was repeatedly photo-bleached (543 nm, 7 cycles of 500 ms at 100% laser power) resulting in 70-80% bleaching of the acceptor. The intensity of the GFP fluorescence before (I_D) and immediately after (I_{DA}) acceptor bleaching was used to calculate the fluorescence recovery: $(I_{DA}-I_D)/I_{DA}$ (Yamamura et al., 2012).

Live-Cell Imaging of GFP-IP_3R3 and Analyses of IP_3R Clustering

Time-lapse images of GFP-IP_3R3 and GFP-IP_3R3(T804A) in HLMVECs were acquired at 37ºC using a Nikon Eclipse TE-2000S microscope with an UltraView confocal head (PerkinElmer Life Sciences), ORCA-ER-1394 camera (Hamamatsu), Ar-ion 488-nm laser, a Plan Apo 100x 1.4 NA objective, and Volocity 5 software (Improvision). Images were acquired every 0.5 s before and for 5 min after stimulation with α-thrombin. To calculate the number of α-thrombin-evoked GFP-IP_3R3 clusters, uneven backgrounds were corrected in all images using the flatten background command (Metamorph). Fluorescence from basal IP_3R3 clusters was subtracted from each post-stimulation image using a projected image from 10 frames collected before stimulation with α-thrombin. Photo-bleaching during image acquisition was disregarded because it was negligible: the bleaching constant of GFP-IP_3R3 fluorescence, determined from exponential curve-fitting, was 0.0005 ± 0.0003 s^{-1}. All images of GFP- IP_3R3 (or immunostained endogenous IP_3Rs) were thresholded and the number of clusters was counted using the Intregated Morphometry Analysis tool (Metamorph).

Quantification of Microtubule Dynamics

Microtubule dynamics were determined from analysis of EB1-GFP tracks in HLMVECs treated with control or EB3 siRNA before and after challenge with α-thrombin. Images were acquired at 37°C at 3-s intervals using the 488-nm Ar-ion laser line of the Nikon confocal microscope described above. Detection, tracking and analysis were performed on the 50-60 frames before and after challenge with α-thrombin using PlustTipTracker software as described previously (Komarova et al., 2012). Microtubule growth rates were determined for microtubules at least 3 μm from the cell cortex. Rates were calculated from the histogram of instantaneous growth rates, that

is the displacement of GFP-EB1-positive microtubule tips between sequential frames. The catastrophe frequency was calculated from the number of shortening (catastrophe) events per min. Because backward gaps can represent pause or shrinkage, the PlustTipTracker software algorithm distinguished between these events based on the gap speed distributions (Applegate et al., 2011). If a microtubule plus end displacement yields a speed slower than the 95th percentile of the speed, the backward gap was assumed to be a pause with 95% confidence. The remaining backward gaps were classified as shrinkage events. The 'Quadrant Scatter Plot' tool, a component of the PlusTipTracker package, was used to determine the relationship between growth rate and growth lifetime (persistence) and to divide microtubules into four sub-populations based on deviation from mean growth speed and growth lifetime (Applegate et al., 2011) (see Figure S2A).

Measurement of Intracellular IP_3 Concentration

The cytosolic IP_3 concentration was measured in single cells with the FRET-based sensor LIBRAvIII (Tanimura et al., 2009). HLMVECs expressing LIBRAvIII were fixed in 4% paraformaldehyde before or after stimulation with α-thrombin (50 nM for 30 s). Using the Zeiss confocal microscope described above, ECFP (donor) fluorescence was excited (458 nm) while recording emission from ECFP with a band-pass BP500/20 nm filter and from the FRET acceptor EYFP with a long-pass LP530 nm filter. An EYFP image was also acquired (excitation at 514-nm; emission collected with the long-pass LP530 nm filter) and used to create an EYFP binary mask (i.e. an image in which each pixel has a value of 0 or 1). By multiplying the FRET signal in each pixel by this binary mask, the FRET analysis included only pixels with detectable fluorescence from the sensor. This modified FRET image was used to compute the relative IP_3 concentration in the cytoplasm for each cell from the ratio of the emission intensities for the acceptor/donor fluorophores when the donor was excited at 458 nm.

Dual-Channel Live-Cell Imaging of Fluorescent Proteins

Dual-channel simultaneous images of GFP-IP_3R3 and R-CEPIA1er, or EGFP-IP_3R3 and EB3-mRFP were acquired using the Zeiss confocal microscope described above. Cells were simultaneously excited with 488-nm and 561-nm lasers, and emitted light was collected by two gallium arsenide phosphide photomultiplier tubes after passing through band-pass filters (500-550 nm and 575-610 nm, respectively).

Generation of *Mapre3*$^{flox/flox}$- *Tie2-Cre*ER^{T2} Mice

All mice were maintained on a C57BL/6 genetic background. *Mapre3*-loxP-targeted transgenic mice, *Mapre3*$^{tm1a(EUCOMM)Wtsi}$, were purchased from the European Mutant Mouse Archive (EMMA). These mice were generated by inserting the promoter-driving selection L1L2-Bact-P cassette composed of an FRT site followed by lacZ sequence and a loxP site at position 30862104 of chromosome 5 upstream of the *Mapre3* critical exon. This first loxP site was followed by neomycin under the control of the human β-actin promoter, SV40 polyA, a second FRT site and a second loxP site. A third loxP site was inserted downstream of the targeted exon(s) at position 30862837 (Figure S5A).

To generate *Mapre3$^{flox/flox}$*, the Neo cassette was removed in all tissue types with flipase-mediated recombination. This was achieved by crossing *Mapre3*$^{\mathrm{tm1a(EUCOMM)Wtsi}}$ with (Gt(ROSA)-26Sortm2(FLP*)Sor mice (Jackson Laboratory), in which widespread expression of the mouse codon-optimized FLP recombinase (FLPo) variant was driven by the *GT(ROSA)26Sor* promoter in a constitutive fashion. Subsequent crossing of *Mapre3$^{flox/flox}$* to the *Tie2-Cre*ER$^{\mathrm{T2}}$ (Korhonen et al., 2009), in which high-fidelity endothelial expression (Forde et al., 2002) of the bacteriophage Cre/LoxP system was driven by regulatory elements from the *Tie2* promoter (Schlaeger et al., 1997) fused to a mutated estrogen receptor ligand-binding domain ERT2 (Feil et al., 1997). We confirmed that treatment with tamoxifen resulted in deletion of *Mapre3* gene in the pulmonary vascular endothelium (Figure S5B) although it is anticipated that Cre-mediated recombination and hence deletion of *Mapre3* gene should occur in the majority of endothelial cell populations (Forde et al., 2002). Genotyping was performed according to a protocol provided by EMMA using primers listed in the supplemental table S7, *DNA Primers Used (for Genotyping)*. No sex ratio disturbances were noted in the construction of strains or experimental progeny.

Four weeks old *Mapre3*$^{\mathrm{flox/flox}}$ (used as a control for the experiments herein) and *Mapre3*$^{\mathrm{flox/flox}}$-*Tie2-Cre*ER$^{\mathrm{T2}}$ mice were treated for 5 days with 75 mg/kg tamoxifen (Sigma-Aldrich) in corn oil administrated intraperitoneally. Mice were used for experiments on day 14 or later after *Cre*-induction.

Fractionation of Pulmonary Vascular Endothelium

Deletion of the EB3 gene in pulmonary endothelium was confirmed by western blot analyses of endothelium-specific fractions (Figure S5B). Fractions were collected after each $k_{f,c}$ trial from *ex vivo* lung preparations. The endothelial lysates were collected via a left atrial cannula by perfusing fractionation buffer (50 mM Tris-Cl, pH 7.8, 0.2% Triton X-100 and protease and phosphatase inhibitor cocktails). Fractions were collected every minute. Fractions 2 and 3, which were positive for VE-cadherin (endothelial marker) and negative for smooth muscle actin (marker of pericytes and smooth muscle cells), were used for assessment of EB3 expression.

Table S1. Expression Constructs Used (Mammalian) in This Study, Related to Figures 1-5.

Name	Residues	Description	Reference
EB3-GFP	FL, 1-281	EB3 with C-terminal EGFP tag	Stepanova et al., 2003
EB3-mRFP	FL, 1-281	mRFP-tagged EB3 in mRFP-N1 plasmid (R. Tsien, UCSD)	Komarova et al., 2009
GFP-EB3ΔAc	1-258	N-terminal EGFP linked (AQAGGSGGAGSGGEGAVDG) to EB3 lacking C-terminal	Komarova et al., 2009
EB3-Ct-mRFP	200-218	C-terminal residues of EB3 tagged with mRFP in mRFP-N1 plasmid (R. Tsien, UCSD)	
EB1-GFP	FL, 1-268	EB1 with C-terminal EGFP tag	Stepanova et al., 2003
mRFP-CLIP-170	FL, 1-1320	CLIP-170 N-terminally tagged with mRFP	Komarova et al., 2005
G-CEPIA1er		Green non-ratiometric Ca^{2+} sensor targeted to ER lumen.	Suzuki et al., 2014
R-CEPIA1er		Red non-ratiometric Ca^{2+} sensor targeted to ER lumen.	Suzuki et al., 2014
GEM-CEPIA1er		Ratiometric Ca^{2+} sensor targeted to ER lumen.	Suzuki et al., 2014
mCherry-er		mCherry targeted to ER lumen	Suzuki et al., 2014
LIBRAvIII		FRET-based IP_3 sensor	Tanimura et al., 2009
GFP-IP_3R1	FL	IP_3R1 with N-terminal EGFP tag	Pantazaka et al., 2011
GFP-IP_3R2	FL	IP_3R2 with N-terminal EGFP tag	Pantazaka et al., 2011
GFP-IP_3R3	FL	IP_3R3 with N-terminal EGFP tag	Wu et al., 2014
GFP-IP_3R3(T804A)	FL	EGFP-tagged IP_3R3 with mutation (T804A) within proposed EB-binding domain	
YFP-STIM1	FL	STIM1 with C-terminal EYFP tag	Zeng et al., 2008

Preparation of the EB3-Ct-mRFP and GFP-IP_3R3(T804A) constructs is described in Experimental Procedures. FL, full-length.

Table S2. Expression Constructs Used (Bacterial) in This Study, Related to Figure 2.

Name	Residues	Description	Reference
(His6)-EB1	FL, 1-281	Full length EB1, N-terminally tagged with His6	Komarova et al., 2009
(His6)-EB3	FL, 1-268	Full length EB3, N-terminally tagged with His6 tag and TEV cleavage site	Komarova et al., 2009
(His6)-EB3-Ct	200-281	C-terminal of EB3 with an N-terminal His6 tag	
(His6)-EB3-ΔAc	1-258	EB3 lacking C-terminal 23 residues, and with an N-terminal His6 tag and TEV cleavage site	

Preparation of the (His6)-EB3-Ct and (His6)-EB3-ΔAc constructs is described in Experimental Procedures.

Table S3. Primary Antibodies Used in This Study, Related to Figures 2, 3 and 6.

Antibody	Species	Source	Application
EB1	Rat	Absea (010811B11)	WB (1:3,000)<br>Figure S1A
EB3	Rat	Absea (010320D02)	WB (1:3,000)<br>Figures S1A, S3E, S4D, S5B
GFP	Mouse	Life Technologies (A11120, A11121)	WB (1:1,000)<br>Figures S3C, S3D
GFP	Rabbit	Life Technologies (A11122)	WB (1:1000)<br>Figures 2B, 2C, S4B
α-tubulin	Mouse	Sigma (T5168)	WB (1:1,000)<br>Figures 6A, 6B, S1A, S4C, S4D
IP_3R1	Rabbit	In house against CLLGHPPHMNVNPQQPA	WB (1:1000)<br>Figures S4B, S4C
IP_3R2	Rabbit	In house against CPDYRDAQNEGKTVRDGELP	WB (1:1,000)<br>Figures S4B, S4C
IP_3R2	Rabbit	Millipore (AB3000)	IC (1:100)<br>Figures S4E, S4F
IP_3R3	Rabbit	AbCam (ab78556)	IC (1:100)<br>Figures 3A, 3B
IP_3R3	Mouse	BD Biosciences (610312)	WB (1:1,000)<br>Figures S3E, S4B, S4C, S4F
VE-cadherin	Goat	Santa Cruz (sc-6458)	WB (1:1,000)<br>Figure S5B
p-MLC-II (Thr18/Ser19)	Rabbit	Cell Signaling (3674P)	WB (1:1,000)<br>Figures 6A, 6B

WB, western blot; IC, immunocytochemistry. The dilutions used for each application are shown.

Table S4. Secondary Antibodies Used in This Study, Related to Figures 2, 3 and 6.

Antibody	Species	Catalogue #	Application
HRP-conjugated anti-mouse	Donkey	715-035-150	WB (1:10,000)<br>Figures 6A, 6B, S1A, S3C, S3D S3E, S4B, S4C, S4D
HRP-conjugated anti-rat	Mouse	212-055-082	WB (1:10,000)<br>Figures S1A, S4D, S5B
HRP-conjugated anti-rabbit	Donkey	711-035-152	WB (1:10,000)<br>Figures 2B, 2C, 6A, 6B, S4B, S4C
HRP-conjugated anti-goat	Donkey	705-055-003	WB (1:10,000)<br>Figure S5B
HRP-conjugated anti-rat IgG, Fcγ fragment-specific	Goat	112-035-071	WB (1:10,000)<br>Figure S3E
Cy5-conjugated anti-rabbit	Donkey	711-495-152	IC (1:200)<br>Figures 3A, S4E, S4F

WB, western blot; IC, immunocytochemistry. HRP, horseradish peroxidase; FITC, fluorescein isothiocyanate; Cy5, Cyanine 5 dye
All secondary antibodies were from Jackson ImmunoResearch Laboratories.

Table S5. The siRNAs Used in This Study, Related to Figures 1, 3 and 6.

Name	Target	Sense sequence	Cat. #
MAPRE1 siRNA	Human EB1	AAACGACCCUGUAUUGCAG	4392420
MAPRE3 siRNA	Human EB3	GAGCAUGAAUACAUCCACAUU	4392420
ITPR3 siRNA	Human IP_3R3	GCAUGGAGCAGAUCGUGUU	4392420
Silencer Select negative control #1			4390843

All siRNAs were from Life Technologies.

Table S6. DNA Primers Used (for QPCR) in This Study, Related to Supplemental Data, Figure S4.

Target	Forward	Reverse
G6PD	TGCCCCCGACCGTCTAC	ATGCGGTTCCAGCCTATCTG
ITPR1 (IP_3R1)	CTGATTCACCCACGAAGGTT	TGCAAATCAGGTGCTTTCTG
ITPR2 (IP_3R2)	GCTCTTGTCCCTGACATTG	CCCATGTCTCCATTCTCATAGC
ITPR3 (IP_3R3)	AGTGAGAAGCAGAAGAAGG	CATCCGGGGGAACCAGTC

All primers were from IDT DNA Technologies. *G6PD*, glucose-6-phosphate dehydrogenase.

Table S7. DNA Primers Used (for Genotyping) in This Study, Related to Figure 6.

Reaction	Forward	Reverse
1	AGGCTGGACCAGGGATTGGCCC	CCAACAGCTTCCCCACAACGG
2	CCACCAGCCAGGGTGACAAGGC	CCCCACCAGGGACTCAGGGACA
3	AGGAACCTAGCCCACCAGCCGC	ACTGATGGCGAGCTCAGACCATCCA
4	AGGAACCTAGCCCACCAGCCGC	GGGCTACAGCAGAGAACAGGGAG

The table provides the list of forward and reverse primers used to validate the genotype of *Mapre3*-loxP-targeted transgenic mice. All primers were from IDT DNA Technologies.

SUPPLEMENTAL REFERENCES

Applegate, K.T., Besson, S., Matov, A., Bagonis, M.H., Jaqaman, K., and Danuser, G. (2011). plusTipTracker: Quantitative image analysis software for the measurement of microtubule dynamics. J. Struct. Biol. *176*, 168-184.

Feil, R., Wagner, J., Metzger, D., and Chambon, P. (1997). Regulation of Cre recombinase activity by mutated estrogen receptor ligand-binding domains. Biochem. Biophys. Res. Commun. *237*, 752-757.

Forde, A., Constien, R., Grone, H.J., Hammerling, G., and Arnold, B. (2002). Temporal Cre-mediated recombination exclusively in endothelial cells using Tie2 regulatory elements. Genesis *33*, 191-197.

Govindan, S., Taylor, E.J., and Taylor, C.W. (2010). Ca^{2+} signalling by P2Y receptors in cultured rat aortic smooth muscle cells. Br. J. Pharmacol. *160*, 1953-1962.

Korhonen, H., Fisslthaler, B., Moers, A., Wirth, A., Habermehl, D., Wieland, T., Schutz, G., Wettschureck, N., Fleming, I., and Offermanns, S. (2009). Anaphylactic shock depends on endothelial G_q/G_{11}. J. Exp. Med. *206*, 411-420.

Morita, T., Tanimura, A., Nezu, A., and Tojyo, Y. (2002). Visualization of inositol 1,4,5-trisphosphate receptor type III with green fluorescent protein in living cells. Cell Calcium *31*, 59-64.

Schlaeger, T.M., Bartunkova, S., Lawitts, J.A., Teichmann, G., Risau, W., Deutsch, U., and Sato, T.N. (1997). Uniform vascular-endothelial-cell-specific gene expression in both embryonic and adult transgenic mice. Proc. Natl. Acad. Sci. USA *94*, 3058-3063.

Stepanova, T., Slemmer, J., Hoogenraad, C.C., Lansbergen, G., Dortland, B., De Zeeuw, C.I., Grosveld, F., van Cappellen, G., Akhmanova, A., and Galjart, N. (2003). Visualization of microtubule growth in cultured neurons via the use of EB3-GFP (end-binding protein 3-green fluorescent protein). J. Neurosci. *23*, 2655-2664.

Yamamura, H., Ikeda, C., Suzuki, Y., Ohya, S., and Imaizumi, Y. (2012). Molecular assembly and dynamics of fluorescent protein-tagged single KCa1.1 channel in expression system and vascular smooth muscle cells. Am. J. Physiol. *302*, C1257-1268.

Zeng, W., Yuan, J.P., Kim, M.S., Choi, Y.J., Huang, G.N., Worley, P.F., and Muallem, S. (2008). STIM1 gates TRPC channels, but not Orai1, by electrostatic interaction. Mol. Cell *32*, 439-448.
